# Abstracts of the Cell Therapy Transplant Canada 2024 Annual Conference

**DOI:** 10.3390/curroncol33010009

**Published:** 2025-12-23

**Authors:** Stephanie A. Maier, Frédéric Barabé, Tobias Berg, Jonathan Bramson, Gwynivere A. Davies, Mahmoud Elsawy, Alejandro Garcia-Horton, Alix Lapworth, Christopher Lemieux, Kylie Lepic, Kristjan Paulson, Michael Radford, Mégane Tanguay, Ram Vasudevan Nampoothiri, Darrell White, Charles Yin, Jonas Mattsson

**Affiliations:** 1 Cell Therapy Transplant Canada, Winnipeg, MB R3P 2R8, Canada; 2 Department of Medicine, CHU de Québec—Université Laval, Quebec City, QC G1V 4G2, Canada; 3 Department of Oncology, McMaster University, Hamilton, ON L8N 3Z5, Canada; 4 Division of Hematology and Hematologic Oncology, McMaster University, Hamilton, ON L8N 3Z5, Canada; 5 Escarpment Cancer Research Institute, Hamilton Health Sciences Centre, Hamilton, ON L8V 5C2, Canada; 6 Division of Hematology and Hematologic Oncology, QEII Health Sciences Centre, Halifax, NS B3H 3A7, Canada; 7 Hamilton Health Sciences, Hamilton, ON L8N 3Z5, Canada; 8 Department of Internal Medicine, Max Rady College of Medicine, University of Manitoba, Winnipeg, MB R3E 0W2, Canada; 9 Department of Medicine, University of Montreal, Montreal, QC H3T 1J4, Canada; 10 Transplant & Cellular Therapy, The Ottawa Hospital, Ottawa, ON K1H 8L6, Canada; 11 Division of Hematological Pathology, University of Alberta, Edmonton, AB T6G 2B7, Canada; 12 Hans Messner Allogeneic Blood and Marrow Transplant Program, Princess Margaret Cancer Centre, Toronto, ON M5G 2C4, Canada

**Keywords:** CAR-T cell, cell therapy, conditioning regimen, graft-versus-host disease (GvHD), hematopoietic cell transplantation, hemoglobinopathy, leukemia, lymphoma, multiple myeloma, relapse-free survival

## Abstract

On behalf of Cell Therapy Transplant Canada (CTTC), we are pleased to present the Abstracts of the CTTC 2024 Annual Conference. The conference was held on 1–3 May 2024 in beautiful Victoria, British Columbia, at the Victoria Conference Centre, and attracted 293 in-person delegates and five virtual attendees. Several plenary sessions were held on topics such as gene therapy for hemoglobin disorders, optimizing donor selection, graft-versus-host disease (GvHD) strategies, collaborative care, survivorship, graft failure, and CAR-T therapy. Poster authors presented their work during a lively and engaging networking reception on Thursday, 2 May, and oral abstract authors were featured during the oral abstract session in the afternoon of Friday, 3 May 2024. Forty-nine (49) abstracts were selected for presentation as posters and six (6) as oral presentations. Abstracts were submitted within four categories: (1) Basic/Translational Sciences, (2) Clinical Trials/Observations, (3) Laboratory/Quality, and (4) Pharmacy/Nursing/Other Transplant Support. The top six (6) abstract authors were invited to give an oral presentation, and the top four (4) poster abstracts were selected to receive an award. All of these were marked as “Award Recipient” within the relevant category. Three abstracts were determined by the peer review panel to be inappropriate for this conference and were not invited to present at the conference, and two authors withdrew their abstract; therefore, five abstract numbers are missing from the list. We congratulate all the 2024 abstract presenters on their research and contributions to the field.

## Simple Summary

The abstracts submitted for the Cell Therapy Transplant Canada (CTTC) 2024 Annual Conference are contained within. The conference was held 1–3 May 2024, and abstracts were submitted for review under four different categories. The best abstracts, as judged by the CTTC abstract review committee, were invited to give a talk during one of the conference sessions, while the remaining abstracts were invited to be showcased as a poster during the conference networking session.

## Abstract 1 (Poster): Expansion and Characterization of Immune Suppressive CD56^bright^CD16^−^ Regulatory Natural Killer Cells for Chronic Graft-Versus-Host Disease (This Abstract Has Subsequently Been Published in Full)

Madeline Lauener ^1,2^, Erin Tanaka ^2^, Ao Mei ^3^, Sayeh Abdossamadi ^1,2^, Elena Ostroumov ^1,2^, Karen Sherwood ^3^, Subra Malarkannan ^4^, Ramon Klein Geltink ^2^ and Kirk R. Schultz ^1,2^

^1^  Michael Cuccione Childhood Cancer Research Program, British Columbia Children’s Hospital Research Institute, University of British Columbia, Vancouver, BC, Canada
^2^  British Columbia Children’s Hospital Research Institute, University of British Columbia, Vancouver, BC, Canada
^3^  Vancouver Coastal Health Authority, Vancouver, BC, Canada
^4^  Versiti Blood Research Institute, Milwaukee, WI, United States of America

**Background:** Chronic graft-versus-host-disease (cGvHD) is a major cause of morbidity after Hematopoietic Stem Cell Transplantation (HSCT). In large HSCT patient cohorts we identified increased numbers of regulatory natural killer (NK_reg_) cells associated with cGvHD suppression and hypothesized that NK_reg_ cells may be important in inducing immune tolerance, being a potential candidate for cGvHD cell therapy. However, NK_reg_ cells represent less than 10% of total NK cells, with total NK cells only making up 1–6% of leukocytes. Therefore, cell expansion is necessary for NK_reg_ cells to be applicable in the clinical setting.

**Purpose:** To expand NK_reg_ cells while maintaining regulatory phenotype and function.

**Methods:** Total NK cells were first expanded with ligands associated with immune tolerance: IL2/4/7/10/18/23, GPR183L, GMCSF. From this analysis, the optimal expansion cytokine (IL2) was combined with known ligands to prevent NK cell differentiation: rapamycin, TGFβ1, NECA, metformin, dexamethasone. The functional characteristics of the expanded cells were evaluated via T-cell suppression assays and the phenotype was measured via flow cytometry. The optimal expansion protocol was then compared in terms of phenotype, function, and metabolism (Seahorse Analyzer) for three different NK cell medias, and NK_reg_ cells from cord versus (vs.) peripheral blood. Further, expanded NK_reg_ protein and gene expression was characterized using the Olink Proximity Extension Assay and bulk RNAseq, respectively. Lastly, we investigated whether functional NK_reg_ cells may be derived from an alternate source, being differentiation from CD34^+^ HSPC’s.

**Results:** The expansion of total NK cells found IL2 to result in the greatest proliferation (up to 100-fold), and the combination of TGFβ1 and/or NECA with IL2 and pulsing of rapamycin prevented NK_reg_ differentiation (up to 200-fold). These expanded cells maintained similar phenotype, transcriptome, and T-cell suppression to fresh NK_reg_ cells. NK_reg_ expansion was greatest in the Immunocult media (up to 300-fold), and NK_reg_ cells from peripheral blood demonstrated significantly greater proliferation than cells from cord blood (65-fold). The metabolic profile of NK_reg_ cells and cytolytic NK cells was not significantly different, though rapamycin induced a lower OCR/ECAR. Additionally, expanded NK_reg_ compared to CD56^dim^ NK cells upregulate several proteins associated with regulatory function, such as TGFβ1, TRAIL, ADA, and IL-10RA (*p* < 0.01). Further, suppressive NK_reg_ cells may also be successfully differentiated and expanded from CD34^+^ cells.

**Conclusions:** Our studies have optimized and described two alternative expansion approaches for deriving functional NK_reg_ cells. Additionally, in evaluating the protein expression and metabolic characteristics of the NK_reg_ cells we have identified useful information regarding NK_reg_ function and differentiation. With further optimization we may work towards preparing these cells as a potential cell therapy for cGvHD.

## Abstract 2 (Poster): Short-Term Exposure of Umbilical Cord Blood CD34^+^ Cells to Human Platelet Lysate and Cytokines Enhances Engraftment (This Abstract Has Subsequently Been Published in Full)

Marie-Ève Rhéaume ^1^, Tony Tremblay ^1^, Isabelle Paré ^1^, Pascal Rouleau ^1^ and Lionel Loubaki ^1,2^

^1^  Medical Affairs and Innovation, Héma-Québec, Montreal, QC, Canada
^2^  Department of Biochemistry, Microbiology and Bioinformatics, Laval University, Quebec City, QC, Canada

**Background:** Umbilical cord blood (UCB) is a widely used source of stem cells in therapies of malignant and non-malignant hematological diseases and metabolic disorders. However, UCB grafts are limited by low numbers of total and stem cells that are associated with delayed hematopoietic and immunologic recovery. Intra-bone marrow (IBM) injection has been proposed as a strategy to bypass homing inefficiencies associated with intravenous (IV) hematopoietic stem cell (HSC) transplantation and to increase the number of HSC that engraft. Despite physical delivery into the BM cavity, many donor cells are rapidly redistributed by vascular perfusion, thus potentially compromising the efficacy of this approach.

**Purpose:** The objective of our study was to evaluate the ability of human platelet lysates (hPL) to improve HSC anchorage into the BM and consequently to improve engraftment.

**Methods:** HSC were isolated using the EasySep™ Human CD34-Positive Selection Kit (STEMCELL Technologies, Vancouver, Canada), and purity was assessed by flow cytometry. HSC were then seeded in the wells of a 24-well microplate and exposed to increasing concentrations of hPL with or without cytokines for 24 h at 37 °C, 5% CO_2_. Following culture, HSC cells chemotaxis to rhSDF-1 was determined *in vitro* using a Transwell and engraftment in NOD SCID gamma (NSG) mice was also evaluated.

**Results:** Short-term exposure of cord blood CD34^+^ cells to a combination of hPL and cytokines resulted in a significant increase (up to 3-fold) in the expression of CD34 antigen on HSC that was closely correlated with a significantly increased migration towards a gradient of rhSDF-1 (up to 7-fold). In addition, IBM injection of CD34^+^ cells previously exposed to hPL^+^ cytokines into NSG mice showed significantly increased engraftment, as measured by human platelet numbers (704.9 ± 230.3 vs. 3193.0 ± 1806.7 human platelets (hplt)/µL of blood; *p* = 0.005), human CD45 (13.2 ± 4.4% vs. 22.2 ± 1.4%) and human CD34^+^ cells (2.4 ± 1.2% vs. 6.6 ± 1.1%) for untreated and treated cells, respectively.

**Conclusions:** The use of hPL^+^ cytokines as a short-term priming treatment for UCB could be an attractive strategy to improve clinical outcomes following IBM injection.

## Abstract 3 (Poster): Dissecting the Oncogenic Role of the miR-106a-363 Cluster in Acute Myeloid Leukemia

Irina A. Maksakova ^1^, Nadine Sperb ^2^, Leo Escano ^1^, Liam MacPhee ^1^, Ariene Cabantog ^1^, Dexter Kim ^1^, Mansen Yu ^1^, Kathrin Krowiorz ^2^, Sarah Grasedieck ^2^, Nicole Pochert ^2^, Christoph Rueß ^2^, Tobias Mätzig ^3^, Reinhild Rösler ^4^, Enrico Calzia ^5^, Lars Palmqvist ^4,6^, Sebastian Wiese ^4^, Linda Fogelstrand ^4,6^, Arefeh Rouhi ^1^ and Florian Kuchenbauer ^1^

^1^  Terry Fox Laboratory, BC Cancer, Vancouver, BC, Canada
^2^  Department of Internal Medicine III, University Hospital Ulm, Ulm, Germany
^3^  Institute of Experimental Hematology, Hannover Medical School, Hannover, Germany
^4^  Core Unit Mass Spectrometry and Proteomics, Ulm University, Ulm, Germany Department of Clinical Chemistry and Transfusion Medicine, Institute of Biomedicine, Sahlgrenska Academy at University of Gothenburg, Gothenburg, Sweden
^5^  Institute of Anesthesiological Pathophysiology and Process Engineering, Ulm University Hospital, Ulm, Germany
^6^  Department of Clinical Chemistry, Sahlgrenska University Hospital, Gothenburg, Sweden

**Background:** High expression levels of miR-106a-3p, miR-106a-5p and miR-20b-3p in pediatric acute myeloid leukaemia (AML) are associated with dismal prognosis, high rate of relapse, low overall and event-free survival and resistance to standard induction chemotherapy. These miRNAs are part of the miR-106a-363 miRNA cluster, a paralogue of the oncogenic miR-17-92 polycistron.

**Purpose:** Multiple studies have linked high expression of the miR-106a-363 cluster to malignant transformation and tumor growth in solid tumors, but very little is known about its role in AML.

**Methods:** We performed extensive *in vitro* and *in vivo* analysis including proteomics, syngeneic transplantations and high resolution respirometry.

**Results:** Analysis of The Cancer Genome Atlas (TCGA) dataset shows that high levels of miRNAs of the miR-106a-363 cluster are associated with inferior overall survival in AML patients. Moreover, expression of most of the miRNAs in the miR-106a-363 cluster, with the exception of miR-18b, is elevated in adverse risk AML and is downregulated in patients in remission.

Forced overexpression of the miR-106a-363 cluster and individual miR-106a-5p, miR-18b-5p and miR-20b-5p accelerates leukaemogenesis in an *in vivo* mouse model of AML. Analyses of the bone marrow proteome of moribund mice show that cellular energetics and mitochondria are heavily involved in leukaemogenicity exhibited by the miR-106a-363 cluster. The proteomics analysis indicates that each of the 106a-363 miRNA cluster members, with the possible exception of miR-92a, provides active and cancer-directed contribution to AML initiation and progression. Some of these pathways, such as proteolysis and stress response, are deregulated by multiple miRNA members simultaneously, increasing the impact of each individual mRNA. In contrast, other pathways are directed by a single and individual miRNA, with miR-106a appearing to account for most of the protein deregulation resulting from the upregulation of the miR-106a-363 cluster.

High-resolution respirometry on bone marrow samples of moribund mice shows that miR-106a overexpression leads to higher routine respiration, leak respiration, maximum OxPhos capacity and Complex IV activity, suggesting its role in elevated metabolism in such cells.

**Conclusions:** In summary, we show that high expression of miR-106a, miR-18b, miR-19b, miR-20b and miR-363 correlates with worse patient survival. We highlight for the first time the oncogenic role of the miR-106a-363 cluster and its individual miRNAs, miR-106a, miR-20b, miR-18b, miR-363, in a primary AML transplantation model, describe deregulated pathways and demonstrate by high resolution respirometry that miR-106a overexpression leads to changes in oxidative phosphorylation.

## Abstract 4 (Poster): Enhancing ASCT Response Prediction in Multiple Myeloma Using an APOBEC Based Risk Score

Afsaneh Panahi ^1,2^, Sarah Grasedieck ^3^, Matthew C. Jarvis ^4^, Faezeh Borzooee1 ^5^, Reuben S. Harris ^4^, Mani Larijani ^5^, Kevin Song ^6^, Arefeh Rouhi ^1,2^ and Florian Kuchenbauer ^1,6^

^1^  Terry Fox Laboratory, British Columbia Cancer, Vancouver, BC, Canada
^2^  Department of Medicine, University of British Columbia, Vancouver, BC, Canada
^3^  Michael Smith Laboratories, Department of Microbiology and Immunology, University of British Columbia, Vancouver, BC, Canada
^4^  Department of Biochemistry, Molecular Biology and Biophysics, University of Minnesota, Minneapolis, MN, United States of America
^5^  Department of Molecular Biology and Biochemistry, Simon Fraser University, Burnaby, BC, Canada
^6^  Leukemia/Bone Marrow Transplant Program of British Columbia, Vancouver General Hospital, Vancouver, BC, Canada

**Background:** The current risk classifiers (ISS, R-ISS) face challenges in accurately predicting autologous stem cell transplantation (ASCT) treatment outcomes in newly diagnosed multiple myeloma (NDMM) patients. New findings related to APOBEC enzymes (DNA editor), and inflammation indicate their involvement in MM progression and their prognostic value for improving patient risk stratification.

**Purpose:** This study aims to develop a novel risk assessment method in MM by integrating APOBEC enzymes and inflammatory markers to improve predictions of ASCT responses and facilitate the design of more efficient therapies.

**Methods:** The Multiple Myeloma Research Foundation CoMMpass study genomics dataset, which integrates mRNA Seq and clinical data from over 700 patients, enabled the assessment of demographic and clinical parameters, cytogenetics, APOBEC family gene expression, and inflammation-modulating cytokines in MM patients. Hazard ratios and Kaplan-Meier survival estimates were computed for all identified features. Utilizing machine learning, we integrated clinically significant variables linked to progression-free survival (PFS) and overall survival (OS) to develop an accurate classification model for a new risk score. Specifically, we assessed this model’s prognostic potential among MM patients receiving CyBorD ± ASCT or VRD ± ASCT treatments, comparing its prognostic value with current staging systems (ISS and R-ISS) through Kaplan-Meier analysis.

**Results:** The machine learning models allowed us to develop a weighted OS/PFS risk score, termed the Editor (APOBEC)-Inflammation (EI) score, using expressions of APOBEC2, APOBEC3B, IL11, TGFB1, TGFB3, and ß-2-microglobulin and LDH serum levels. This novel score exhibited superior performance compared to ISS and R-ISS, enabling more accurate risk stratification among NDMM patients. This novel risk score demonstrated superior performance compared to ISS and R-ISS, enabling more accurate risk stratification among MM patients. Sub-analyses among CyBorD ± ASCT or VRD ± ASCT-treated patients consistently demonstrated the EI-score’s enhanced predictive power over ISS and R-ISS. Importantly, the EI-score identified specific patient subsets that did not benefit from ASCT despite receiving VRD or CyBorD therapies (Figure 1).

**Conclusions:** The superiority of the EI-score over current models like ISS and R-ISS in predicting ASCT response in MM indicates the potential of the molecular marker-based approach to make more precise ASCT decisions and shape tailored therapeutic strategies.

## Abstract 5 (Poster): *Timp2* Is a Positive Regulator of Hematopoietic Stem Cell Fitness

Sanjay Saw, Ronak Shetty, Hui Fang, Paul Waterhouse and Rama Khokha

Princess Margaret Cancer Centre, University Health Network, Toronto, ON, Canada

**Background:** Tissue inhibitor of metalloprotease-2 (TIMP2) is one of four members of the TIMP family, canonically known for endogenous metalloprotease inhibition. TIMP2 has been found to influence matrix stabilization, inflammation, invasion, and the rejuvenation of aging tissue. Recently, it has been shown that *Timp2* expression was remarkably high in acute myeloid leukemia compared to matched TCGA normal tissues and Genotype-Tissue Expression (GTEx) data, among 27 listed cancers [1]. Despite being involved in key biological processes and hematological malignancy, the role of TIMP2 has never been explored in hematopoiesis.

**Methods and Results:** To uncover the role of TIMP2 in hematopoiesis, we first examined the localization of this protein in the mouse bone marrow by in situ immunofluorescence staining. TIMP2 localized with key components of the hematopoietic stem cell (HSC) niche such as in sinusoidal endothelium and H-type endothelium. Importantly, exploration of publicly available data revealed a high expression of *Timp2* in HSCs, which declined in the differentiating progenitor population, indicating a role of *Timp2* in HSC function. In fact, flow cytometric analysis of 3-month-old whole body *Timp2*^–/–^ mouse blood showed reduced B-lymphocytes and lower frequency of their progenitors in the bone marrow. Also, there was a significant reduction of HSC frequency in the bone marrow of 8-month-old *Timp2*^–/–^, but not 3-month-old mice, suggesting the importance of *Timp2* in the maintenance of HSCs and their lineage populations. Following a competitive transplant of wildtype (WT) and *Timp2*^–/–^ bone marrow (50/50), a continuous decline of *Timp2*^–/–^ donor derived chimerism was noted in the recipients’ peripheral blood over a span of 5-months. At the 5-month timepoint, WT-HSC represented 75% (vs. 21% for *Timp2*^–/–^) of the reconstituted bone marrow. Moreover, a lower frequency of *Timp2*^–/–^ donor derived lymphoid cells were also observed in the recipient’s blood compared to WT-donor, demonstrating an impaired competence and lineage bias of *Timp2*^–/–^ HSC during bone marrow replenishment. To evaluate the regeneration capacity of these HSCs, *Timp2*^–/–^ mice, WT recipients transplanted with *Timp2*^–/–^ bone marrow, as well as WT-control mice were administrated weekly doses of fluorouracil (5-FU). Lower survival (*p* < 0.01) of mice with *Timp2*^–/–^ HSCs demonstrated that *Timp2*^–/–^ HSCs were less capable of tolerating chemotherapeutic insult (5-FU), in a cell-autonomous fashion, suggesting a compromised bone marrow regeneration capacity of HSCs lacking *Timp2.*

**Conclusions:** Overall our data suggests that *Timp2* is necessary for HSC maintenance and that its absence compromises the ability of HSCs to generate downstream effector cells and tolerate chemotherapeutic insults.

**Reference**

1. Wang, D.-D.; Xu, W.-X.; Chen, W.-Q.; Li, L.; Yang, S.-J.; Zhang, J.; Tang, J.-H. Identification of TIMP2 as a Prognostic Biomarker and Its Correlation with Tumor Immune Microenvironment: A Comprehensive Pan-Cancer Analysis. *J. Oncol.* **2022**, *2022*, 1–12. https://doi.org/10.1155/2022/9133636.

## Abstract 6 (Poster): Cost-Effectiveness of Brexucabtagene Autoleucel for the Treatment of Relapsed/Refractory B-Cell Acute Lymphoblastic Leukemia in Patients Aged 18 Years or Older in Canada

Andre C Schuh ^1^, Geoffrey GJ Reid ^2^, Tomas Spousta ^3^, Frank van Hees ^3^, Nate Smith ^3^ and Brett Doble ^2^

^1^  Princess Margaret Cancer Centre, Toronto, ON, Canada
^2^  Kite, a Gilead Company, Santa Monica, CA, United States of America
^3^  Maple Health Group LLC, New York, NY, United States of America

**Background:** Despite currently available treatments, the prognosis for adults with relapsed/refractory B-cell acute lymphoblastic leukemia (R/R B-ALL) remains poor, highlighting the need for new therapeutic strategies. Brexucabtagene autoleucel (BREXU-CEL) was approved for the treatment of R/R B-ALL by Health Canada in November 2022 and was recommended for reimbursement by the Canadian Agency for Drugs and Technologies in Health (CADTH) in April 2023 and by Institut National d’Excellence en Santé et Services Sociaux (INESSS) in August 2023.

**Purpose:** To estimate the cost-effectiveness from a Canadian payer perspective of BREXU-CEL versus blinatumomab (BLIN), inotuzumab ozogamicin (INO), and salvage chemotherapy (CHEMO), in patients aged 18 years or older with R/R B-ALL.

**Methods:** A partitioned-survival model comprising the health states ‘event-free survival’ (EFS), ‘progressed disease’ (PD) and ‘death’ was used to estimate health outcomes and costs over a lifetime horizon for each treatment considered. Efficacy and safety data were obtained from ZUMA-3 for BREXU-CEL (median follow-up 37.3 months), TOWER for BLIN, and INO-VATE for INO and CHEMO; a naïve comparison among studies was conducted. For BREXU-CEL we used data from patients aged 18 years or older in line with the product label. Patients in the BREXU-CEL arm not receiving infusion were assigned EFS and overall survival (OS) as observed for the comparator treatments. Standard parametric models were used to extrapolate EFS and OS for all treatments. Utilities for the EFS and PD health state were derived from ZUMA-3. Patients alive at 3 years were assigned general population mortality, to which a standardized mortality ratio of 1.09 was applied, and general population utilities. Unit costs were obtained from public databases or from the literature. List prices were used for all treatments. All monetary values are in 2022 Canadian dollars. Costs and health outcomes were discounted at 1.5% annually.

**Results:** Compared with BLIN, INO, and CHEMO, BREXU-CEL resulted in 11.63, 8.98, and 10.65 life-years gained, and 8.96, 7.12, and 8.35 quality-adjusted life-years (QALYs) gained per patient, respectively. The incremental costs of BREXU-CEL versus BLIN, INO, and CHEMO were $220,625, $172,004, and $441,643, respectively. BREXU-CEL’s incremental cost-effectiveness ratios were $24,615/QALY versus BLIN, $24,150/QALY versus INO, and $52,917/QALY versus CHEMO. Results were robust to changing model assumptions. At list price, BREXU-CEL had a 96% probability of being the most cost-effective intervention compared to BLIN, INO, and CHEMO at a willingness-to-pay threshold of $100,000 per QALY gained.

**Conclusions:** BREXU-CEL substantially improves the life-expectancy of patients with R/R B-ALL compared to BLIN, INO, and CHEMO and added life-years are spent in good health. Moreover, BREXU-CEL is cost-effective at list price versus BLIN, INO, and CHEMO in Canada at a willingness-to-pay threshold of $100,000/QALY.

## Abstract 7 (Poster): Elucidating the Effects of Donor-Specific Induced-Pluripotent Stem Cell Secretome on Recovery After Acute Myocardial Infraction

Elise Rody ^1^, Jeremy Zwaig ^2^, Ida Derish ^2,3^, Kashif Khan ^2^, Nadia Giannetti ^4^, Adel Schwertani ^4^ and Renzo Cecere ^1^

^1^  Department of Surgery, Division of Cardiac Surgery, McGill University Health Centre, Montreal, QC, Canada
^2^  Department of Medicine, McGill University, Montreal, QC, Canada
^3^  Department of Surgical Innovation and Interventional Sciences, McGill University, Montreal, QC, Canada
^4^  Department of Medicine, Division of Cardiology, McGill University Health Centre, Montreal, QC, Canada

**Background:** During a myocardial infarction, or “heart attack”, the heart muscle tissue (myocardium) can lose billions of cells. Patient biological response to heart attacks and the subsequent treatment is highly individualized and ill-understood. The focus of regenerative therapies has thus far been administration, via direct injection, peripheral injection, patch, or other methods, of various stem cell types in hopes of myocardial remuscularization. However, the efficacy of such treatments has proven unreliable in clinical settings and new approaches are being sought. Enter cell-free stem cell therapies. By leveraging the stem cell secretome via the collection of “conditioned media”, cell-free regenerative therapies utilize the beneficial signals emitted by stem cells encouraging recovery of injured myocardium.

**Purpose:** Secretome content has been found to be extremely variable depending on cell source. We hypothesized that induced pluripotent stem cell (iPSCs) secretome-derived from healthy donors would better protect cardiomyocytes from cell death and hypertrophy after acute myocardial injury than iPSC secretome-derived from dilated cardiomyopathy patients.

**Methods:** Peripheral blood mononuclear cells were collected from donors and cryopreserved until transfection. Once thawed, CD34^+^ were isolated and expanded over a 1-week period. Cells were then reprogrammed into iPSCs, and the best colonies were selected and passaged 6–10 times. Once cells were 80–90% confluent, media was replaced with serum-free media for 24 h. Media was then collected, filtered, and used to treat the AC16 immortalized cardiomyocyte cell line during reperfusion directly following a 24-h exposure to hypoxia.

**Results:** Proteomic analysis of the iPSC secretome revealed increased levels of proteins involved in stress response, hypoxia response, negative regulation of apoptotic process and detoxification of reactive oxygen species in healthy donor conditioned media. This supported findings that the iPSC secretome from healthy donors better protected cardiomyocytes from cell death after 5 and 24 h of reperfusion. Patient secretome also protected cell viability compared to non-treated cells. Per contra, only healthy donor secretome treatment reduced signs of maladaptive cardiac remodelling as seen by the analysis of hypertrophy.

**Conclusions:** Our results stand in contrast to the idea that patients could “make their own medicine” as our results support the idea that not all secretomes are equal. This research speaks to the feasibility of producing nearly infinite amounts of patient-derived cell-free therapies as a treatment for myocardial infarctions. Future studies are needed to carefully evaluate the specific disease mechanisms when deciding to prescribe secretome-based therapies.

## Abstract 8 (Poster): Determining Patient-Specific Sensitivity to Doxorubicin-Induced Cardiotoxicity

Ludovic Mouttet ^1^, Elise Rody ^2^, Ida Derish ^1,3^ and Renzo Cecere ^1,2^

^1^  Department of Surgical Innovation and Interventional Sciences, McGill University, Montreal, QC, Canada
^2^  Department of Surgery, Division of Cardiac Surgery, McGill University Health Centre, Montreal, QC, Canada
^3^  Department of Medicine, McGill University, Montreal, QC, Canada

**Background:** Doxorubicin (Dox) is the most common chemotherapy drug used to treat hematological and solid tumors in patients. However, Dox is well-known to promote doxorubicin-induced cardiotoxicity (DIC), posing the most significant risk for cancer survivors to develop irreversible cardiac dysfunction and heart failure after treatment. Unfortunately, various models of cardiotoxicity *in vitro* lack reproducibility, and the role of regulated cell death pathways remain unclear.

**Purpose:** To develop novel therapies, a more accurate cellular model and deeper understanding of the pathways involved is necessary to comprehend the mechanisms which lead to DIC. This project utilizes patient-specific induced pluripotent stem cell-derived cardiomyocytes (iPSC-CMs) to develop a robust Dox injury model to mimic what occurs *in vivo* and allow the elucidation of cellular processes contributing to Dox sensitivity in patients.

**Methods:** First, iPSC-CMs were generated from healthy, cardiomyopathic or DIC donor peripheral blood mononuclear cells (PBMCs). Thawed PBMCs were expanded for 7 days before undergoing transfection and, from only 16 mL of peripheral blood, donor-specific iPSCs were obtained. CMs were then obtained using established differentiation protocols. Second, we induced iPSC-CM proliferation following a novel protocol developed by Mass et al. in 2021. Finally, we validated an optimal Dox injury model by treating iPSC-CMs with increasing Dox concentrations (0, 0.1, 0.5 and 1 µM) over 24, 36 and 48 h. Pre- and post-Dox treated iPSC-CMs were compared using Crystal Violet viability assays and AlamarBlue to determine metabolic activity.

**Results:** Successful transfection of peripheral blood-derived iPSCs was confirmed by the staining and subsequent visualization of pluripotency markers: OCT-4, SSEA4, NANOG and TRA-1-60. We successfully reproduced expansion of the normally quiescent iPSC-CM and showed a significant increase in the number of iPSC-CMs after replating (2–4-fold expansion). When characterizing iPSC-CMs, we confirmed the expression of cardiac markers GATA4, SERCA2a, cTNNT2, and CX43. After incubating iPSC-CMs with Dox, we found significant decreases in viability and metabolic activity (*p* < 0.01), when comparing 36 h and 48 h incubations to 24 h, and increasing Dox concentrations, observing a concentration-dependent effect.

**Conclusions:** We defined the optimal conditions to induce DIC *in vitro*, as 0.5 μM Dox incubated for 48 h. Furthermore, we validated an approach for expanding iPSC-CMs allowing the generation of a high throughput DIC model, replicable in other types of iPSC-CM-based disease modelling. This model also has the potential to be used as a toxicity and efficacy assay for novel therapies. In the long term, this project paves the way for the study of patient-specific susceptibility to Dox injury, ultimately translating to a clinical impact and improving cancer and cardiovascular outcomes.

## Abstract 9 (Oral): Guided Lymphocyte Immunopeptide Derived Expansion (GLIDE) and Infusion: A Phase I Clinical Trial of Anti-Minor Histocompatibility Antigen Expanded T-Cells to Target Leukemia Relapsing After HLA-Matched Allogeneic Hematopoietic Stem Cell Transplantation (Award Recipient—Basic and Translational)

Sylvie Lachance ^1^, Jean-Sébastien Delisle ^1^, Brian Leber ^2^, Felix Couture ^3^, Stephanie Thiant ^4^, Natasha Kekre ^5^, Radia Sidi Boumedine ^4^, Cedric Carli ^4^, Ann Brasey ^4^, Imran Ahmad ^1^, Nadia Bambace ^1^, Lea Bernard ^1^, Sandra Cohen ^1^, Thomas Kiss ^1^, Jean Roy ^1^, Guy Sauvageau ^1^, Olivier Veilleux ^1^, Lambert Busque ^1^, Claude Perreault ^6^ and Denis Claude Roy ^1^

^1^  Institut Universitaire d’Hémato-oncologie, Greffe et Thérapie Cellulaire, Hôpital Maisonneuve-Rosemont, Montreal, QC, Canada
^2^  Department of Medicine, McMaster University, Hamilton, ON, Canada
^3^  Le Centre hospitalier universitaire de Québec, Quebec City, QC, Canada
^4^  Centre de recherche de l’Hôpital Maisonneuve-Rosemont, Montreal, QC, Canada
^5^  Blood and Marrow Transplant Program, The Ottawa Hospital, Ottawa, ON, Canada
^6^  Institute for Research in Immunology and Cancer, Department of Medicine, Université de Montreal, Montreal, QC, Canada

**Background:** Relapse remains the major impediment to successful hematopoietic stem cell transplantation (HSCT). Graft-versus-leukemia (GVL) immune reaction is protective against relapse. In the HLA-matched setting, the GVL effect is mediated by donor T-cell immune response against host minor histocompatibility antigens (MiHAs) found on the surface of leukemic cells. Donor lymphocyte infusions (DLI) have demonstrated efficacy to treat relapse after HSCT hampered by the risk of harmful graft-versus-host (GVH) reaction due to the lack of specificity of lymphocytes infused. To generate potent and selective anti-leukemia activity, we have developed a strategy for the *ex vivo* selection and expansion of T cells targeting MiHAs preferentially expressed on hematopoietic cells with the objective of augmenting GVL while limiting the risk of GVH.

**Methods:** In an open-label, multi-centre phase I clinical trial (National Clinical Trial (NCT) 03091933), patients with hematologic malignancies who relapsed after an HLA-matched HSCT were selected after identifying a targetable MiHA based on polymorphism analysis of the recipient and donor. After donor cell collection and manufacturing, recipients were lymphodepleted followed by infusion of GLIDE, consisting of donor T-lymphocytes expanded *ex vivo* following exposure to dendritic cells coated with immunogenic MiHAs peptides, at a single target dose of 4 × 10^7^ viable T-cells/m^2^. GLIDE cell characteristics were evaluated using multi-parameter flow cytometry and T-cell receptor (TCR) sequencing. Patients were followed up for one-year post-GLIDE infusion for toxicity and clinical outcome measures.

**Results:** Ten patients with hematologic malignancies, AML (7), NHL (2), and myelofibrosis (1), in relapse after HSCT, median age of 58, were treated with GLIDE. These patients had relapsed at a median of 12 months (range 5–126 months) after HSCT. GLIDE cells were personalized to target single MiHAs in 9 patients and two MiHAs in 1 patient. GLIDE cell products were directed at 9 different MiHAs on 6 different HLA-class 1 molecules (HLA-A02:01, A24:02, B07:02, B18:01, B44:02, and B44:03). Final GLIDE products consisted mainly of T-cells (median: 98.7%), with CD8 > CD4 > NKT > NK cells. Most CD8^+^ T cells were effector memory and central memory T cells (Figure 1). At the end of the expansion period, CD8^+^ T cells expressed activation markers (CD25^+^ 67%) and low levels of exhaustion (PD1^+^, PDL1^+^, CD57^+^, KLRG1^+^). GLIDE cells had a more restricted TCR β chain repertoire than initial lymphopheresis cells. There were no infusional toxicities. One patient developed thrombocytopenia and cGvHD. According to TCR β sequencing data, T cells present in the adoptively transferred anti-MiHA GLIDE product persisted in peripheral blood up to one year after infusion. Survival at one year was observed in 4 patients with 3 in sustained remission (Figure 2).

**Conclusions:** This pilot study demonstrates the feasibility and the safety of GLIDE production and administration to patients relapsing after allogeneic HSCT. The remissions and low occurrence of GvHD underline the clinical potential of this approach to target leukemia and hematologic malignancies.

## Abstract 10 (Poster): Defining the Optimal Cryoprotectant Agent Conditions for Cryopreservation of Regulatory T-Cells for Tolerogenic Therapy

Sarjana Alam ^1^, Rebecca Mercier ^2^, Lavinia Ionescu ^3^, Lori West ^1,3,4,5^, Jason P. Acker ^1,2^ and Esme Dijke ^1,4,5,6^

^1^  Department of Laboratory Medicine and Pathology, University of Alberta, Edmonton, AB, Canada
^2^  PanTHERA CryoSolutions Inc, Edmonton, AB, Canada
^3^  Department of Pediatrics, University of Alberta, Edmonton, AB, Canada
^4^  Alberta Transplant Institute, Edmonton, AB, Canada
^5^  Canadian Donation and Transplantation Research Program, Edmonton, AB, Canada
^6^  Alberta Precision Laboratories, Edmonton, AB, Canada

**Background:** Regulatory T cell (Treg) tolerogenic therapy is a promising innovation for the treatment of autoimmune diseases and transplant rejection, with early clinical trials underway. Defining the optimal Treg cryopreservation conditions is beneficial for successful clinical implementation. Conventional cryoprotectant agent (CPA) dimethyl sulfoxide (DMSO) is cytotoxic, contributing to poor Treg recovery post-thaw. CPAs such as small molecule carbohydrate-derived ice recrystallization inhibitors (IRIs) or dextran may offer lower toxicity and greater cryoprotection.

**Purpose:** To develop an optimized Treg cryopreservation protocol, we assessed toxicity and ability to improve post-thaw cell viability and function of various CPA conditions.

**Methods:** Peripheral blood mononuclear cells were isolated from whole blood of healthy volunteers (*n* = 3) using density gradient centrifugation. Tregs were isolated by a magnetic bead-based isolation protocol and expanded for 13 days using an optimized expansion protocol. Treg phenotype was assessed by flow cytometry. Expanded cells were suspended in (1) base solution, (2) 10% DMSO (standard protocol), (3) 5% DMSO, (4) 5% DMSO with 10 mM IRI, and (5) 5% DMSO with 5% dextran for 0, 2 and 4 h at various temperatures (4, 22, 37 °C). In addition, aliquots of each cell suspension were cryopreserved, thawed, resuspended in culture medium with and without interleukin-2 (IL-2) and incubated at 37 °C for 48 h. Treg recovery and viability were assessed by an automated cell counter.

**Results:** After isolation, 88–91% of cells had the CD4^+^CD25^+^FOXP3^+^ Treg phenotype. This phenotype was maintained during expansion, with 80–94% of cells being CD4^+^CD25^+^FOXP3^+^ on day 13. Cells suspended in 10% DMSO had lower recovery after 2 and 4 h at 37 °C only compared to the other conditions. No clear differences were observed in Treg recovery and viability when suspended in 5% DMSO alone, with IRI, and with dextran (*n* = 3).

Immediately post-thaw, cells cryopreserved in 5% DMSO with 5% Dextran showed the highest recovery and viability compared to the other conditions (*n* = 2). After 24 h in culture, however, Tregs cryopreserved in 5% DMSO only maintained the highest number of live cells. At 48 h, no clear differences in viability and cell numbers were observed between the different conditions, but Tregs cultured with IL-2 had increased growth compared to those cultured without.

**Conclusions:** Our findings suggest that decreased DMSO concentration may be key to optimizing Treg recovery after cryopreservation. Identifying the CPA condition best suited to Tregs will facilitate significant progress in the development of an optimized cryopreservation protocol for tolerogenic cellular therapy.

## Abstract 11 (Poster): Thymus-Derived Regulatory T-Cells as a Universal Off-the-Shelf Anti-Inflammatory Therapy: Technology Transfer in Preparation for a Phase I Clinical Trial (Award Recipient—Basic and Translational)

Sabine Ivison ^1^, Katie MacDonald ^1^, Lieke Sanderink ^1^, Macyn Leung ^1^, Majid Mojibian ^1^, Jessica Qing Huang ^1^, Vivian Fung ^1^, Lori West ^2^, Esme Dijke ^2^, Erin Bleker ^3^, Andrew Campbell ^3^, Mohammed Al Aklabi ^3^, Janna Kosuska ^4^, Gayle Piat ^4^, Kevin Hay ^5^ and Megan Levings ^1^

^1^  British Columbia Children’s Hospital Research Institute, University of British Columbia, Vancouver, BC, Canada
^2^  Department of Pediatrics, University of Alberta, Edmonton, AB, Canada
^3^  Children’s Heart Centre, British Columbia Children’s Hospital, Vancouver, BC, Canada
^4^  Alberta Cell Therapy Manufacturing, University of Alberta, Edmonton, AB, Canada
^5^  Cumming School of Medicine, University of Calgary, Calgary, AB, Canada

**Background:** CD4^+^ T regulatory cells (Tregs) are a subset of T cells characterized by high expression of CD25 and FOXP3. Their ability to suppress immune-based pathology and promote tissue repair make Tregs a promising universal anti-inflammatory cell therapy. Despite encouraging results in early-phase clinical trials, widespread testing is complicated by limited Treg availability and the high cost of autologous therapy. An abundant source of pure, allogeneic Tregs enabling an “off-the-shelf” therapy would facilitate widespread clinical testing. The thymus, routinely removed and discarded during pediatric heart surgery, is a rich source of naïve Tregs which can be isolated by CD25 expression with low likelihood of contamination by activated effector T cells. Thymic Tregs (tTregs) are potently suppressive even under inflammatory conditions and resistant to inflammation-induced lineage instability, a concern with blood-derived Treg therapy. This off-the-shelf tTreg product will be piloted in a phase I trial for prevention of graft vs. host disease (GvHD) post allogeneic hematopoietic stem cell transplant (HSCT).

**Purpose:** To transfer the manufacturing of thymus-derived Tregs from a research lab to a GMP facility to enable the collection of feasibility and quality data in preparation for a phase I clinical trial.

**Methods:** CD25^+^CD8^−^ thymocytes are isolated using magnetic beads (StemCell Technologies; Vancouver, BC, Canada), cryopreserved and shipped to the Alberta Cell Therapy Manufacturing (ACTM) GMP facility for expansion using Dynabeads Treg Xpander (Thermo Fisher Scientific; Waltham, MA, USA) before harvest, bead removal and freezing. Thawed cells are subjected to release and reference testing, including phenotyping for identity, purity and cytokine production, suppression assays, epigenetic testing and a flow cytometry-based residual bead assay. In addition, product efficacy and toxicology are tested using a xenogeneic (xeno) GvHD model in NOD SCID gamma mice.

**Results:** We expanded 3 tTreg batches in the ACTM process development lab and are currently expanding another 3 batches in ACTM cleanrooms. The current manufacturing process yields 3–5 × 10^9^ tTregs from 10–30 × 10^6^ CD25^+^CD8^−^ thymocytes in a 14-day expansion protocol. Phenotyping routinely shows that the thawed drug product has >70% apoptosis negative cells, is >80% FoxP3^+^ and contains <1% CD8^+^ CD4^−^ cells. Treg identity is confirmed using a GMP-compatible Treg-specific demethylated region (TSDR) assay (PureQuant, Thermo Fisher Scientific; Waltham, MA, USA) and a novel suppression assay standardized in our lab which enables quantification of both costimulatory molecule removal on B cells and inhibition of proliferation. The DynaCellect system (Thermo Fisher Scientific; Waltham, MA, USA) provides rapid and thorough removal of magnetic beads as quantified by a flow cytometry-based assay developed in our lab. Xeno GvHD and toxicology studies are ongoing.

**Conclusions:** This data will be presented to Health Canada in support of a clinical trial application for a phase I trial to reduce GvHD after allogeneic HSCT.

## Abstract 12 (Poster): Beyond CAR-T: Harnessing Natural Killer (NK) Cells for Improved Outcomes in High-Risk Neuroblastoma

Teesha C. Baker ^1,2^, Ramon Klein Geltink ^1,3^, Gregor Reid ^1,2^ and Kirk R. Schultz ^1,2^

^1^  British Columbia Children’s Hospital, Vancouver, BC, Canada
^2^  Department of Pediatrics, University of British Columbia, Vancouver, BC, Canada
^3^  Department of Pathology and Laboratory Medicine, University of British Columbia, Vancouver, BC, Canada

**Background:** Neuroblastoma is a pediatric malignancy with a high relapse rate and poor prognosis. Despite treatment advancements, survival rates for high-risk neuroblastoma remain disappointingly low. Innovative solutions like chimeric antigen receptor (CAR)-T therapy show promise by leveraging the body’s natural cancer-fighting abilities and achieving high remission rates. However, CAR-T therapy has significant drawbacks: (1) It often results in severe side-effects, (2) It is typically effective only for liquid tumours, and (3) Its use is limited to autologous cell infusions.

Natural killer (NK) cells provide a promising solution to these issues. CAR-NK cell therapy offers the following advantages: (1) Less toxicity, such as cytokine release syndrome or immune effector cell-associated neurotoxicity syndrome, and minimal to no graft-versus-host disease, (2) The ability to penetrate solid tumours, and (3) No MHC-restriction for antigen recognition, which allows the use of off-the-shelf NK cells, making the treatment available to more patients.

**Purpose:** We aim to establish the groundwork for a phase I clinical trial targeting children with neuroblastoma. This trial intends to explore novel treatments with the potential to improve survival rates and reduce treatment-associated morbidities.

**Methods:** To evaluate NK cell expansion rates in an *ex vivo* environment, we will isolate peripheral blood mononuclear cells (PBMCs) from healthy donors and further isolate NK cells using the NK Isolation kit (StemCell Technologies, Vancouver, BC, Canada). Once isolated, NK cells will be cultured with K562 cells in NK expansion media (NKEM). Cell counts and viability will be monitored, with feeder cells added every 7 days during the expansion process. After expansion, we will assess NK cell cytotoxicity to confirm their efficacy against cancer cells.

**Results:** Currently, we are testing different conditions for the *ex vivo* expansion of NK cells, both with and without the use of feeder cells. While using IL-2 alone results in a minimal 5–10-fold expansion over 14 days, feeder cells, such as K562 cells (with or without specific ligands or cytokines), are commonly used to support the expansion of NK cells in culture, achieving higher expansion rates. However, it is important to remove these feeder cells to avoid potential adverse effects when administering the expanded NK cells to patients. To address this, we plan to establish a unique transduced K562 cell line expressing the 4-1BBL ligand and membrane-bound IL-21 on the cell surface, which has previously shown a median 21,000-fold expansion in 21 days (https://dx.doi.org/10.3791/2540).

**Conclusions:** CAR-NK cell therapy demonstrates promising results in targeting solid tumors with reduced patient toxicity. Our study focuses on developing methods that can be used in Good Manufacturing Practice (GMP) clinical production for the treatment of neuroblastoma in children.

## Abstract 13 (Poster): Three-Year Analysis of ZUMA-12: A Phase II Study of Axicabtagene Ciloleucel (Axi-Cel) as First-Line Therapy in Patients with High-Risk Large B-Cell Lymphoma (LBCL) (This Abstract Has Subsequently Been Published in Full)

Julio Chavez ^1^, Michael Dickinson ^2^, Javier Munoz ^3^, Matthew Ulrickson ^4^, Catherine Thieblemont ^5^, Olalekan O. Oluwole ^6^, Alex F. Herrera ^7^, Chaitra S. Ujjani ^8^, Yi Lin ^9^, Peter A. Riedell ^10^, Natasha K. Kekre ^11^, Sven de Vos ^12^, Christine Lui ^13^, Jacob Wulff ^13^, Chad M. Williams ^13^, Weixin Peng ^13^, Ioana Kloos ^13^, Hairong Xu ^13^ and Sattva S. Neelapu ^14^

^1^  Moffitt Cancer Center, Tampa, FL, United States of America
^2^  Sir Peter MacCallum Department of Oncology, The University of Melbourne, Melbourne, VIC, Australia
^3^  Banner MD Anderson Cancer Center, Phoenix, AZ, United States of America
^4^  Banner MD Anderson Cancer Center, Gilbert, AZ, United States of America
^5^  Service d’hémato-oncologie, Hôpital Saint-Louis, Paris, France
^6^  Vanderbilt-Ingram Cancer Center, Nashville, TN, United States of America
^7^  City of Hope, Duarte, CA, United States of America
^8^  Clinical Research Division, Fred Hutch Cancer Center, Seattle, WA, United States of America
^9^  Mayo Clinic Cancer Center, Rochester, MN, United States of America
^10^  David and Etta Jonas Center for Cellular Therapy, University of Chicago, Chicago, IL, United States of America
^11^  Department of Medicine, The Ottawa Hospital, Ottawa, ON, Canada
^12^  David Geffen School of Medicine, University of California at Los Angeles, Los Angeles, CA, United States of America
^13^  Kite, a Gilead Company, Santa Monica, CA, United States of America
^14^  Department of Lymphoma and Myeloma, The University of Texas MD Anderson Cancer Center, Houston, TX, United States of America

**Background:** Axi-cel is an autologous anti-CD19 chimeric antigen receptor (CAR) T-cell therapy approved for the treatment of relapsed/refractory (R/R) LBCL. ZUMA-12 (NCT03761056) is a Phase II, multicentre, single-arm study of axi-cel as part of first-line treatment in patients (pts) with high-risk LBCL. We previously published our findings of the primary efficacy analysis [1]. Here, we report updated efficacy and safety outcomes from ZUMA-12 in all pts treated with axi-cel after a median follow-up of ≥40 months.

**Methods:** Adult pts met criteria for high-risk LBCL, double-/triple-hit histology per investigator or an International Prognostic Index (IPI) score ≥ 3, plus a positive interim positron emission tomography (PET) per Lugano Classification (Deauville score 4/5) after 2 cycles of an anti-CD20 and anthracycline–containing regimen. Pts underwent leukapheresis, followed by lymphodepleting chemotherapy on Days -5, -4, and -3, and a single axi-cel infusion (2 × 10^6^ CAR-T cells/kg) on Day 0. Non-chemotherapy bridging was allowed. Primary endpoint was complete remission (CR) rate (per investigator). Secondary endpoints were overall response rate (ORR), duration of response (DOR), event-free survival (EFS), progression-free survival (PFS), overall survival (OS), safety, and levels of CAR-T cells in blood and cytokines in serum.

**Results:** As of 3 May 2023, 42 pts were enrolled and 40 were treated with axi-cel, with a median follow-up of 40.9 months (range, 29.5–50.2). 37 pts were evaluable for response (centrally confirmed double-/triple-hit histology or IPI score ≥ 3). The CR rate was 86% (95% CI, 71–95), and the ORR was 92% (95% CI, 78–98). Pts with centrally confirmed double-/triple-hit histology (*n* = 10) had a CR rate of 90%. Responses were ongoing in 73% of response-evaluable pts at data cutoff. Medians for DOR, EFS, PFS, and OS were not reached. The 36-month estimates for DOR, EFS (Figure 1), PFS, and OS (Figure 2) were 82%, 73%, 75%, and 81%, respectively.

All treated pts (*n* = 40) experienced adverse events (AEs) of any grade and 88% of patients had grade ≥ 3 AEs. Similar to the primary analysis, the most common any-grade AEs were pyrexia (100%), headache (70%), and neutrophil count decreased (55%). No new cases of cytokine release syndrome (CRS) or neurologic events (NE) occurred since the prior data cut and all cases of CRS and NE reported were resolved by data cutoff. Any grade prolonged cytopenias occurred in 9 pts (*n* = 7 neutrophil count decreased) and were resolved by data cutoff. There were 8 deaths due to progressive disease (PD) (*n* = 5) and other causes not related to axi-cel (*n* = 3). Two of the 8 deaths (1 PD and 1 esophageal adenocarcinoma) occurred after the primary analysis.

**Conclusions:** In this updated analysis of ZUMA-12, axi-cel demonstrated a high rate of durable responses and no new safety signals. Axi-cel may benefit pts exposed to fewer prior therapies and those with high-risk LBCL, a population with high unmet need and poor outcomes after standard first-line chemoimmunotherapy.

**Reference**

1. Neelapu, S.S.; Dickinson, M.; Munoz, J.; Ulrickson, M.L.; Thieblemont, C.; Oluwole, O.O.; Herrera, A.F.; Ujjani, C.S.; Lin, Y.; Riedell, P.A.; et al. Axicabtagene ciloleucel as first-line therapy in high-risk large B-cell lymphoma: the phase 2 ZUMA-12 trial. *Nat. Med.* **2022**, *28*, 735–742. https://doi.org/10.1038/s41591-022-01731-4.

## Abstract 14 (Poster): Development of a Combined Risk Assessment Model for Venous Thromboembolism and Bleeding in Hematopoietic Stem Cell Transplantation Patients

Jodi Chiu ^1,2^, Uday Deotare ^1,2,3^, Anargyros Xenocostas ^1,2,3^ and Alejandro Lazo-Langner ^1,2,4^

^1^  Department of Medicine, Division of Hematology, Western University, London, ON, Canada
^2^  Schulich School of Medicine & Dentistry, Western University, London, ON, Canada
^3^  Blood and Marrow Transplant Program, London Health Sciences Centre, London, ON, Canada
^4^  Department of Epidemiology and Biostatistics, Western University, London, ON, Canada

**Background:** Venous thromboembolism (VTE) and bleeding are common complications in hematopoietic stem cell transplant (HSCT) patients. Balancing these risks is challenging due to concurrent pancytopenia and coagulopathy, with risk factors for these complications being understudied.

**Purpose:** We aimed to derive a combined bleeding and VTE risk assessment model (RAM) for the immediate 90-day post-transplant period.

**Methods:** We conducted a retrospective cohort study of adult patients who underwent HSCT between 1 January 2011, and 3 December 2021, at a Canadian tertiary care centre. Patients were followed from transplant until the event of interest, day 90 post-transplant, or death. Primary VTE outcome was confirmed thrombosis, including proximal deep vein thrombosis, pulmonary embolism, or thrombosis of unusual sites. Primary bleeding outcome was major or clinically significant non-major bleeding unrelated to anticoagulation, as per International Society of Thrombosis and Hemostasis definition. Group characteristics were compared using appropriate tests. Optimal cut-off points for continuous variables were estimated with receiver operating characteristic (ROC) curves. Predictors for VTE and bleeding were confirmed with multiple variable logistic regression. Final risk scores were derived based on weighted variables and compared using Cox regression with non-parametric bootstrapping for internal validation.

**Results:** A total of 476 patients were included. VTE occurred in 47 patients (9.8%), and bleeding unrelated to anticoagulation occurred in 32 patients (6.7%) within 90 days post-HSCT.

The VTE RAM included: second central venous catheter insertion (2 points), and previous cancer treatment with steroids (1 point). The high-risk VTE group (≥2 points) had a cumulative VTE incidence of 31.8% versus 7.6% in the low-risk group. The high-risk group was associated with higher 90-day mortality (15.9% vs. 3.2%, *p* < 0.001).

The bleeding RAM included: baseline (transplant day 0) platelet count of <90 × 10^3^/μL (1 point), and baseline hemoglobin of <96 g/L (1 point). The high-risk bleeding group (2 points) had a cumulative bleeding incidence of 17.2% versus 4.3% in the low-risk group. The high-risk group was associated with higher 90-day mortality (12.6% vs. 2.6%, *p* < 0.001).

Amongst the low-risk bleed group, VTE cumulative incidence was 10.03%, with 31.03% in the high-risk VTE group versus 8.33% in the low-risk VTE group (*p* < 0.001). The positive VTE group was associated with higher 90-day mortality (10.26% vs. 1.72%, *p* = 0.001).

**Conclusions:** We derived the VTE and Bleeding in Marrow Transplant (VBMT) model (Figure 1) for predicting VTE and bleeding within 90-days post-transplant, complementing existing criteria for bleeding and thrombosis. In low-risk bleeding patients, we recommend strong consideration of chemical VTE prophylaxis, particularly in the high-risk VTE group. This RAM facilitates bleeding and thrombosis risk stratification, guiding VTE prophylaxis and surveillance strategies for HSCT patients.

## Abstract 15 (Poster): Post-Transplant Cyclophosphamide Is Associated with Reduced Rate of Secondary Malignancies Post-Allogeneic Stem Cell Transplant

Nihar Desai ^1^, Mariana Pinto Pereira ^1^, Mats Remberger ^3^, Rajat Kumar ^1,2^, Dennis D. Kim ^1,2^, Auro Viswabandya ^1,2^, Arjun D. Law ^1,2^, Wilson Lam ^1,2^, Ivan Pasic ^1,2^, Armin Gerbitz ^1,2^, Igor Novitzky-Basso ^1,2^, Jonas Mattsson ^1,2^ and Fotios V. Michelis ^1,2^

^1^  Hans Messner Allogeneic Blood and Marrow Transplantation Program, Division of Medical Oncology and Hematology, Princess Margaret Cancer Centre, Toronto, ON, Canada
^2^  Department of Medicine, Section of Medical Oncology and Hematology, University of Toronto, Toronto, ON, Canada
^3^  Department of Medical Sciences, Clinical research and development unit, Uppsala University Hospital, Uppsala, Sweden

**Background:** Advancements in allogeneic hematopoietic cell transplantation (allo-HCT) over the past decade have significantly enhanced patient outcomes with more long-term survivors. However, these survivors face an increased risk of secondary malignancies (SM) post-transplant.

**Methods:** Patients aged 18–75 years received allo-HCT at our centre from October 2015 to May 2021. Baseline variables were summarized using descriptive statistics. Cumulative incidence (CumI) of SM was determined using the competing risk method with death as the competing event. Overall survival (OS) was estimated using the Kaplan Meier method. A *p*-value of <0.05 was considered statistically significant.

**Results:** Among 884 patients post allo-HCT, 73 (8.3%) developed SM, including 19 (26%) with non-metastatic squamous cell carcinoma (SCC) and basal cell carcinoma (BCC) of the skin (Table 1). The median age at diagnosis of SM was 62 years (range, 53–68). The median time from allo-HCT to development of SM was 19.3 months (range, 3.1–30.5), and 26.6 months (range, 11.6–40.7 months) after excluding patients with post-transplant lymphoproliferative disorder (PTLD). The median follow-up post-transplant was 32.9 months (range, 8.3–51.8).

The CumI of SM for the entire cohort was 6.8% at 3 years and 9.4% at 5 years. After excluding non-metastatic skin BCC and SCC, these figures were 5.4% at 3 years and 6.7% at 5 years. Most patients (76.3%) received reduced intensity conditioning (RIC), and 637 (72%) received dual T-cell depletion using anti-thymocyte globulin (ATG) with post-transplant cyclophosphamide (PTCy) as GvHD prophylaxis. 721 patients (81%) received total body irradiation, with 621 (70%) receiving ≤200 centigrays (cGy).

The use of PTCy was associated with a reduced risk of SM in both univariate (hazard ratio (HR) 0.56; *p* = 0.025) and multivariable analysis (HR 0.55; *p* = 0.02) (Figure 1a). Patients with any history of smoking before allo-HCT had a higher likelihood of developing SM on univariate (HR 1.89; *p* = 0.009) and multivariable analysis (HR 1.73; *p* = 0.02) (Figure 1b). The median follow-up after diagnosis of SM was 17 months (range, 6–47). The OS after diagnosis of SM was 53% (range, 37.8–66.2) at 3 years and 49.9% (range, 34.5–63.5) at 5 years for the 54 patients with SM excluding skin SCC/BCC (Figure 1c).

Ten patients with SM subsequently developed an additional SM at a median of 22.5 months (range, 8–44.7) after the first SM, and 39.1 months (range, 24.7–51.5) after allo-HCT. Half of them had PTLD as their first SM. Nine of these patients were alive at the time of last follow up.

**Conclusions:** The use of PTCy results in a reduced incidence of SM following allo-HCT. This may be related to the significant reduction in GvHD with PTCy use. Additionally, patients with a history of smoking pre-transplant have a higher likelihood of developing SM. These findings warrant confirmation in a larger cohort. Moreover, the survival of allo-HCT patients with SM is favorable, warranting routine long-term follow up.

## Abstract 16 (Poster): Eltrombopag in the Treatment of Post Allogeneic Hematopoietic Stem Cell Transplantation Cytopenia: Efficacy, Response Durability and Potential Cost Benefit of Early Drug Tapering

Rawan Al-Omari ^1^, Ram Vasudevan Nampoothiri ^1^, Eshetu G. Atenafu ^2^, Ali Sakhdari ^3^, Jeffrey H. Lipton ^1^, Dennis D. Kim ^1^, Wilson Lam ^1^, Arjun Law ^1^, Armin Gerbitz ^1^, Ivan Pasic ^1^, Igor Novitzky-Basso ^1^, Auro Viswabandya ^1^, Fotios V. Michelis ^1^, Jonas Mattsson ^1^, and Rajat Kumar ^1^

^1^  Hans Messner Allogeneic Transplant Program, Princess Margaret Cancer Centre, Toronto, ON, Canada
^2^  Department of Biostatistics, Princess Margaret Cancer Centre, Toronto, ON, Canada
^3^  Department of Pathology, Princess Margaret Cancer Centre, Toronto, ON, Canada

**Background and Purpose:** The utilization of Eltrombopag (EPAG) in the management of post-allogenic hematopoietic stem cell transplant (HSCT) cytopenia has exhibited promising outcomes. Isolated thrombocytopenia and poor graft function (PGF) are factors that adversely influence transplant outcomes and patient well-being. This study aims to assess EPAG efficacy in this context as a primary outcome and evaluate early EPAG tapering post complete response (CR) for cost-efficiency and response durability.

**Methods:** In this retrospective study, we enrolled 39 patients (69.2% male) with a median age at transplant of 61.0 years (Range: 23–73 years) who underwent allogenic HSCT. Among these, 89.7% (*n* = 35) received EPAG for PGF, while 10.3% (*n* = 4) were treated for isolated thrombocytopenia (primary or secondary). The median treatment duration was 16.3 weeks (Range: 4.4–120.1 weeks). EPAG was initiated at 50 mg daily dose and escalated to a maximum of 150 mg daily. The overall response rate (ORR) was evaluated after 4 weeks of EPAG maximum dose. CR was defined as a sustained platelet count ≥ 50 × 10^9^/L, hemoglobin (Hb) ≥ 100 g/L, and absolute neutrophil count (ANC) ≥ 1.5 × 10^9^/L without transfusions or growth factor support. Partial response (PR) was defined as platelet count 20–50 × 10^9^/L or Hb 7–10 g/L or ANC 0.5–1.5 × 10^9^/L. Early EPAG tapering was defined as start of tapering after 4 weeks of achieving CR. The pre-EPAG cost estimate included supportive care expenses (transfusion costs and hospital admissions due to cytopenia-related complications), Post-EPAG costs included the drug cost (50 mg bill cost: 130 CAD) plus supportive care expenses. All patient characteristics and costs are presented in Table 1.

**Results:** With a median follow-up time of 12.4 months (Range 2.4–46.9) the ORR after 4 weeks of EPAG treatment was 71.8% (*n* = 28), with 48.7% (*n* = 19) achieving CR, and 23.1% (*n* = 9) PR (Figure 1). The median response for platelets, hemoglobin and neutrophils were 2.71, 2.65, and 2.00 weeks, respectively. Median duration between CR to EPAG tapering was 1.00 week (Range 0–18.3) with 10 of 19 responders being tapered earlier than 4 weeks of CR, and all patients had durable responses after stopping the drug with no significant side effects. Median adjusted post-EPAG cost in early tapering was significantly lower than actual post-EPAG cost (*p* = 0.01). There was no significant difference among median adjusted post-EPAG cost in early tapering compared to total cost pre-EPAG (*p* = 0.80). A limitation that we did not calculate is the values of patient quality of life and health system costs.

**Conclusions:** EPAG demonstrates promise as a potential safe treatment for PGF and isolated thrombocytopenia post-allogeneic HSCT. Implementing early tapering of EPAG could be a safe and cost beneficial approach given the high drug cost and durable responses after stopping the drug.

## Abstract 17 (Poster): CAR-T Therapy for the Treatment of Relapsed/Refractory CD19-Positive Large B-Cell Lymphoma: Ottawa Real-World Experience

Xiu Xia Sherry Tan ^1^, Melissa Maltez ^1^, Ranjeeta Mallick ^2^, Linda Hamelin ^1^, Sheryl McDiarmid ^1^, Michael Kennah ^1^, Harold Atkins ^1^, and Natasha K. Kekre ^1^

^1^  Department of Medicine, The Ottawa Hospital, Ottawa, ON, Canada
^2^  Ottawa Hospital Research Institute, Ottawa, ON, Canada

**Background:** Chimeric antigen receptor T-cell (CAR-T) therapies have shown remarkable efficacy in relapsed/refractory large B-cell lymphoma (LBCL) in clinical trials. However, real-world studies assessing the feasibility, efficacy, and safety of CAR-T therapy are needed. Canada’s limited number of CAR-T centres and widespread patient population may pose challenges in effectively implementing CAR-T therapy. Therefore, we aimed to examine early CAR-T use in LBCL patients at our institution, where CAR-T referrals come from across the country, to identify Canadian specific outcomes and guide future care.

**Methods:** We conducted a retrospective review of patients treated with commercial CAR-T products, axicabtagene ciloleucel (axi-cel) or tisagenlecleucel (tisa-cel), at The Ottawa Hospital between January 2020 to July 2022.

**Results:** Fifty-one patients (30 male, 59%), with median age of 62 (range, 24–82), received CAR-T products: axi-cel (*n* = 27, 53%) or tisa-cel (*n* = 24, 47%). The indications for therapy included diffuse large B-cell lymphoma (*n* = 33, 65%), transformed follicular lymphoma (*n* = 17, 33%) and primary mediastinal large B-cell lymphoma (*n* = 1, 2%). Patients presented with relapsed (*n* = 30, 59%) or refractory (*n* = 21, 41%) disease and received a median of 2 (range, 2–6) previous lines of therapy.

Patients were referred from 13 centres across Canada and travelled a median distance of 655 km (range 3–3659) to receive treatment in Ottawa (Figure 1). The median time from last progression to referral was 15 days (range 0–200), with significant delays in referring patients in the out-of-province cohort (34 versus 9 days, *p* < 0.0001). The median time from referral to consult (7 versus 8 days) and referral to CAR-T infusion (64 versus 65 days) was not different for in-versus out-of-province patients. The median time from apheresis to CAR-T infusion was 36 days (range 26–81), with tisagenlecleucel being significantly longer (*p* < 0.001).

Out of 49 evaluable patients, the overall response rate was 57%. With a median follow-up of 219 days (95% CI: 6–722), the median progression free and overall survival was 257 days (95% CI: 92–not evaluable (NE)) and 422 days (95% CI: 106–NE) respectively. There was a trend towards longer progression free survival for in-province (280 days, 95% CI: 142–NE) versus out-of-province (115 days, 95% CI: 91–NE) patients. Forty-seven patients experienced cytokine release syndrome (grade ≥ 3 in 3, 6%), and 20 experienced neurotoxicity (grade ≥ 3 in 6, 12%).

**Conclusions:** Our results confirm that Ottawa can effectively provide CAR-T treatments in a timely fashion for patients nationwide. This study demonstrates that distance to Ottawa is not a barrier to CAR-T administration, but there is a delay in referring centres sending timely CAR-T referrals at time of progression. As CAR-T therapy gains prevalence, comprehensive real-world evidence considering country size, patient transportation, geography, and logistics will be crucial for understanding CAR-T therapy distribution and accessibility.

## Abstract 18 (Poster): The Use of Ganciclovir or Valganciclovir Does Not Increase Relapse Risk Following Allogeneic Stem Cell Transplantation in Patients with Acute Myeloid Leukemia or Myelodysplastic Syndrome

Nihar Desai ^1^, Carol Chen ^1^, Ivan Pasic ^1,2^, Wilson Lam ^1,2^, Arjun Law ^1,2^, Armin Gerbitz ^1,2^, Auro Viswabandya ^1,2^, Fotios Michelis ^1,2^, Rajat Kumar ^1,2^, Jonas Mattsson ^1,2^, Igor Novitzky-Basso ^1,2^, and Dennis Dong Hwan Kim ^1,2^

^1^  Hans Messner Allogeneic Transplant Program, Division of Medical Oncology and Hematology, Princess Margaret Hospital, Toronto, ON, Canada
^2^  Department of Medicine, University of Toronto, Toronto, ON, Canada

**Background:** Leukemia relapse following allogeneic hematopoietic cell transplantation (HCT) in acute myeloid leukemia (AML), or myelodysplastic syndrome (MDS) remains a significant challenge. Loss of heterozygosity (LOH) in major histocompatibility complex (MHC) genes or down-expression of MHC genes have been identified as a potential mechanism leading to relapse post-HCT. Recent work [1] (Toffalori et al., ASH 2023 abstract 362) suggested the genotoxicity of ganciclovir as one of the mechanisms of LOH-MHC, leading to post-HCT relapse. It has never been replicated whether ganciclovir or valganciclovir are associated with an increased risk of relapse. We attempted to replicate the hypothesis that the use of (val)ganciclovir is associated with increased relapse incidence in a large single-centre cohort.

**Methods:** Patients with AML/MDS who underwent HCT at Princess Margaret Cancer Centre between January 2015 and March 2023 were evaluated. Cumulative incidence of relapse (CIR) and non-relapse mortality (NRM) was determined considering competing events, respectively. Overall survival (OS) was determined using the Kaplan-Meier method. A *p*-value of <0.05 was considered statistically significant.

**Results:** A total of 727 patients with AML/MDS underwent HCT, of whom 624 (85.8%) were cytomegalovirus (CMV) seropositive, constituting the primary group for analysis. Baseline characteristics are summarized in Table 1. The median follow-up duration was 24.9 months (range, 8–54.5) among survivors, and 146 (23.4%) patients relapsed at a median of 6 months (range, 2.8–10.2) post-HCT.

Of the 350 episodes (56.1%) of CMV viremia, 317 were treated with (val)ganciclovir. Patients receiving (val)ganciclovir exhibited a lower CIR at 24 months, 19.6% (95% confidence interval: 15.4–24.2) compared to controls, 25.8% (20.8–31.1, *p* = 0.03, Figure 1A). Regarding specific donor types, CIR for mismatched unrelated donor (MMUD) transplants was 33% (20.5–46.1) for patients who received (val)ganciclovir compared to 35.6% (16.1–55.7) in controls (*p* = 0.95); the corresponding numbers for haploidentical transplants were 21.5% (12.7–31.9) and 24.6% (11–41), respectively (*p* = 0.68).

The OS at 24 months was 56.4% (50.7–61.7) for patients who received (val)ganciclovir and 68% (62.1–73.1) for controls (*p* = 0.006, Figure 1B), whereas the NRM rate at 2 years was 28.1% (23.2–33.2) in those who received (val)ganciclovir and 14.6% (10.8–18.9) in the control group (*p* < 0.001, Figure 1C).

On univariate analysis, exposure to (val)ganciclovir (HR: 0.60; *p* = 0.003), and the use of myeloablative conditioning (MAC) (HR: 0.49; *p* = 0.004) were associated with decreased incidence of relapse. Conversely, a high–very high disease risk index (HR: 2.52; *p* < 0.001) and the use of MMUD (HR: 1.72; *p* = 0.01) increased the likelihood of relapse. Multivariable analysis confirmed that the exposure to (val)ganciclovir does not increase the risk of relapse, rather reducing the relapse risk (Table 1).

**Conclusions:** Treatment with (val)ganciclovir is not associated with an increased risk of relapse post allo-HCT in patients with AML/MDS, including MMUD and haploidentical transplant recipients.

**Reference**

1. Toffalori, C.; Bergamin, R.; Antonello, A.; Tortorelli, A.F.; Orofino, G.; Cristante, M.; Della Volpe, L.; Milite, S.; Calonaci, N.; Gandolfi, G.; et al. Time of Origin and Environmental Drivers of Leukemia Immune Escape after Transplantation. *Blood* **2023**, *142*, 362–363. https://doi.org/10.1182/blood-2023-188623.

## Abstract 19 (Poster): One-Day Conditioning Regimen for Allogeneic Stem Cell Transplantation in Patients with Graft Failure: A Single-Centre Experience

Nihar Desai ^1^, Carol Chen ^1^, Igor Novitzky-Basso ^1,2^, Ivan Pasic ^1,2^, Wilson Lam ^1,2^, Arjun Law ^1,2^, Armin Gerbitz ^1,2^, Auro Viswabandya ^1,2^, Fotios V. Michelis ^1,2^, Dennis D. Kim ^1,2^, Jonas Mattsson ^1,2^, and Rajat Kumar ^1,2^

^1^  Hans Messner Allogeneic Transplant Program, Division of Medical Oncology and Hematology, Princess Margaret Hospital, Toronto, ON, Canada
^2^  Department of Medicine, University of Toronto, Toronto, ON, Canada

**Background:** Graft failure (GF) after allogeneic hematopoietic stem cell transplant (HCT) is a life-threatening complication necessitating a rescue HCT. Conventional conditioning regimens are typically administered over four to five days and may not be well tolerated in these patients with GF and associated complications. Exploring shortened, less intensive preparative regimens shows promise in mitigating complications and improving patient outcomes.

**Purpose:** To evaluate the outcomes of patients receiving a one-day conditioning regimen for graft failure.

**Methods:** We conducted a retrospective analysis of all patients experiencing GF (primary and secondary) at Princess Margaret Cancer Centre between February 2019 & June 2023. We report the outcomes of patients receiving the one-day conditioning regimen (Fludarabine 30 mg/m^2^, cyclophosphamide 2 g/m^2^, alemtuzumab 20 mg (fixed dose), and 200 cGy total body irradiation [1]. The graft-versus-host disease (GVHD) prophylaxis was variable, with one patient receiving post-transplant cyclophosphamide (PTCy). The stem cell dose between first and rescue transplant was compared using the paired *t*-test, and overall survival (OS) was estimated using the Kaplan-Meier method. A *p*-value of <0.05 was considered statistically significant.

**Results:** Ten patients (8 primary GF; 2 secondary GF) underwent a second HCT using the one-day conditioning regimen (Table 1). The median age was 60.3 years (56.4–64.7), seven were male. The interval between the two HCTs was 67 days (55–83.5). Seven patients received grafts from a different donor. The median CD34^+^ stem cell dose was 8.1 × 10^6^/kg (5.7–9.6) for the second HCT, compared with 5.4 × 10^6^/kg (4.8–5.8) for the first HCT (*p* = 0.01). All patients successfully engrafted neutrophils, eight engrafted platelets. The median time to neutrophil and platelet engraftment was 21 days (16.5–22.5) and 20 days (17.3–21), respectively. T-cell chimerism at day 30 after second HCT showed complete donor chimerism (>95%) in seven patients. Notably, none of the patients developed grade III/IV acute graft-versus-host disease. At a median follow-up of 9.7 months (range, 5.4–24.4) after the second HCT, seven patients were alive and were in remission (Figure 1).

**Conclusions:** The one-day conditioning regimen is well tolerated by these vulnerable patients with cytopenias and associated complications. The regimen has a high success rate of engraftment with low rates of GVHD. Further comparison of this regimen with more conventional protocols is underway.

**Reference**

1. Kanda, J.; Horwitz, M.E.; Long, G.D.; Gasparetto, C.; Sullivan, K.M.; Chute, J.P.; Morris, A.; Hennig, T.; Li, Z.; Chao, N.J.; et al. Outcomes of a 1-day nonmyeloablative salvage regimen for patients with primary graft failure after allogeneic hematopoietic cell transplantation. *Bone Marrow Transplant.* **2011**, *47*, 700–705. https://doi.org/10.1038/bmt.2011.158.

## Abstract 20 (Poster): Outcomes of Allogeneic Hematopoietic Stem Cell Transplantation in Adults Sixty-Five and Older

Veronica Ramirez ^1^, Gizelle Popradi ^2^ and Jonathan How ^2^

^1^  Internal Medicine Residency Training Program, McGill University, Montreal, QC, Canada
^2^  Division of Hematology, McGill University Health Centre, Montreal, QC, Canada

**Background:** Allogeneic hematopoietic stem cell transplant (HCT) is increasingly used in older populations, with patients over 60 comprising more than 20% of Canadian recipients in the last decade. However, there is no consensus on upper age limit across institutions. Since 2018, our centre moved from a hard age cutoff of 70 to a performance-based transplant eligibility evaluation. Better understanding of outcomes in the older cohort may inform practice in the Canadian setting.

**Purpose:** Determine transplant-related outcomes in HCT patients 65 and over and evaluate the impact of patient age and other characteristics on overall survival (OS) and relapse-free survival (RFS).

**Methods:** We performed a retrospective study of a cohort of patients 65 and older who underwent HCT from January 2012 to August 2023 at the Royal Victoria Hospital. OS and RFS were estimated using Kaplan-Meier analysis and compared with logrank tests. Cumulative incidences of non-relapse mortality (NRM) and acute graft-versus-host disease (aGVHD) were compared by chi-squared tests.

**Results:** We identified 79 patients aged 65 to 74, with median Karnofsky Performance Status of 100% (range 60–100%) and HCT comorbidity index (HCT-CI) of 1 (range 0–6). Patient and disease characteristics are outlined in Table 1.

Median OS and RFS were 9.9 months, with 40% OS at one year and 32% at two years. There was no significant difference by age (65–69 vs. ≥70) or donor type (Figure 1). High-risk HCT-CI patients had significantly lower OS (*p* = 0.01) and RFS (*p* = 0.02) compared to low/intermediate groups (Figure 2). Patients transplanted since 2018 had two-year OS of 40%, compared to 20% between 2012–2017, suggesting a non-significant trend towards improvement driven by a decrease in NRM (45% vs. 72%, *p* = 0.3).

NRM for the cohort was 17.7% at day 100 and 44.6% at one year, without significant difference by age or donor type. Leading causes of one-year NRM were infection (66%) and aGVHD (12%).

Relapse rate was 8.9% at day 100 and 16.2% at one year, without significant difference by age, HCT-CI or donor type. Graft failure rate was 16.5% and significantly higher in those aged ≥70 than 65–69 (*p* = 0.02).

Grade II-IV aGVHD was 5.1% at day 100 and 32.4% at one year. Matched-related patients, of whom 19% received T-cell depletion, had significantly higher one-year rate of grade II–IV aGVHD compared to matched and mismatched unrelated patients (*p* = 0.001), who all received alemtuzumab with conditioning.

**Conclusions:** At our Canadian institution, HCT patients 70 and older had similar prognosis to those aged 65–69. This was potentially confounded by a non-significant trend to improved NRM and OS over time, as patients 70 and older were more commonly transplanted in the latter half of the study period. In this cohort, high HCT-CI predicted for lower OS. Our results suggest patients over 70 can be transplanted just as successfully as patients 65–69 but that careful consideration should be given to selecting those with low/intermediate comorbid risk.

## Abstract 21 (Poster): A Dream or Reality: Consideration of ‘Bloodless’ Hematopoietic Stem Cell Transplants for Jehovah’s Witness Patients (This Abstract Has Subsequently Been Published in Full)

Michael MacNeill ^1^, Adrienne Fulford ^2^, Deanna Caldwell ^3^, and Uday Deotare ^1,2,3,4,5^

^1^  Department of Medicine, Schulich School of Medicine and Dentistry, Western University, London, ON, Canada
^2^  London Regional Cancer Program, London Health Sciences Centre, London, ON, Canada
^3^  Victoria Hospital, London Health Sciences Centre, London, ON, Canada
^4^  Division of Hematology, Department of Medicine, Schulich School of Medicine and Dentistry, Western University, London, ON, Canada
^5^  The Centre for Quality, Innovation and Safety, Department of Medicine, Schulich School of Medicine and Dentistry, Western University, London, ON, Canada

**Background:** Hematopoietic stem cell transplantation (HSCT) is an important part of treatment for many hematologic conditions. The high-dose chemotherapy used in HSCTs puts patients at risk of significant cytopenias that often necessitate hematopoietic support via blood product transfusions. Certain populations, including Jehovah’s Witnesses, are unable to receive blood product transfusions during their transplant and thus, in the past, have been seen as not suitable candidates for HSCTs. However, there has been growing evidence for so-called ‘bloodless’ HSCT protocols, attained through pre-transplant optimization, non-transfusion hematopoiesis support, and minimalization of blood loss, can be safe and effective in these populations.

**Purpose:** Describing the implementation of a ‘bloodless’ HSCT protocol at a Canadian centre for Jehovah’s Witness patients. This regimen was utilized for three different Jehovah’s Witness inpatients, who did not consent to most blood-derived products, at London Health Sciences Centre (LHSC) since 2021.

**Methods:** Assessing the most recent and relevant literature, LHSC’s Hematology Division constructed a ‘bloodless’ HSCT regimen. Key components involved pre-transplant hematopoietic optimization and post-transplant management with prophylactic hematopoietic support, bleeding prevention, minimizing unnecessary blood loss, as well as reactive management for severe cytopenias and significant bleeding This regimen was utilized for three different Jehovah’s Witness inpatients, who did not consent to most blood-derived products, at LHSC since 2021. Two of the patients underwent salvage autologous HSCTs in the setting of relapsed/recurrent primary central nervous system diffuse large B-cell lymphoma while the other patient received tandem (two) HSCTs for high-risk multiple myeloma.

**Results:** None of the patients had a significant bleeding event. Minor bleeding events, predominantly mucositis, resolved with site-specific management. No patients developed a hemoglobin nadir below 80 g/L. All patients did have significant thrombocytopenias and neutropenias with thrombopoietin receptor agonists (TPO-RAs) and granulocyte colony-stimulating factor (G-CSF) administration. All the patients’ cell lines had normalized by the time of discharge and all patients were hospitalized for less than 30 days, similar to centre-specific experience with ‘regular’ autologous HSCTs. All patients are doing well clinically with the two lymphoma patients having an ongoing complete metabolic response.

**Conclusions:** Successful implementation of a ‘bloodless’ HSCT regimen at a Canadian centre with findings of feasibility and safety are in line with previous experiences in other centres through specialized and carefully planned protocols. Prospective trials would be beneficial to further elucidate the risks or disadvantages associated with the ‘bloodless’ protocols. Additionally, aspects of the ‘bloodless’ regimen could be implemented into ‘regular’ HSCTs to reduce blood product requirements.

## Abstract 22 (Poster): Hematopoietic Cell Transplantation Trends and Outcomes in Canada: A Registry-Based Cohort Study (This Abstract Has Subsequently Been Published in Full)

Matthew D. Seftel ^1,2^, Ivan Pasic ^3,4^, Gaganvir Parmar ^1,4^, Oliver Bucher ^5^, David S. Allan ^1,6^, Sita Bhella ^3,4^, Kevin Anthony Hay ^2,7,8^, Oluwaseun Ikuomola ^5^, Grace Musto ^5^, Anca Prica ^3,4^, Erin Richardson ^5^, Tony H. Truong ^9^ and Kristjan Paulson ^10,11^

^1^  Canadian Blood Services, Vancouver, BC, Canada
^2^  Division of Hematology, Department of Medicine, University of British Columbia, Vancouver, BC, Canada
^3^  Hans Messner Allogeneic Blood and Marrow Transplantation Program, Division of Medical Oncology and Hematology, Princess Margaret Cancer Centre, University Health Network, Toronto, ON Canada
^4^  Department of Medicine, University of Toronto, Toronto, ON, Canada
^5^  Department of Epidemiology, CancerCare Manitoba, Winnipeg, MB, Canada
^6^  Department of Medicine and Biochemistry, Microbiology & Immunology, University of Ottawa, Ottawa, ON, Canada
^7^  Division of Hematology, Department of Medicine, University of Calgary, Calgary, AB, Canada
^8^  Terry Fox Laboratory, British Columbia Cancer Research Institute, Vancouver, BC, Canada
^9^  Division of Pediatric Hematology/Oncology, Department of Pediatrics, University of Calgary, Calgary, AB, Canada
^10^  Cell Therapy Transplant Canada, Winnipeg, MB, Canada
^11^  Department of Medical Oncology and Haematology, CancerCare Manitoba, Winnipeg, MB, Canada

**Background:** Hematopoietic cell transplantation (HCT) is an established therapy for hematologic malignancies and serious non-malignant blood disorders. Despite its curative potential, HCT is associated with substantial toxicity and health resource utilization. Effective delivery of HCT requires complex hospital-based care, which limits the number of HCT centres in Canada. In Canada, the quantity, indications, temporal trends, and outcomes of patients receiving HCT are not known.

**Methods:** This was a retrospective cohort study of the first transplants reported to the Cell Therapy Transplant Canada (CTTC) registry between 2000 and 2019. We determined overall survival (OS) and non-relapse mortality (NRM), categorizing the cohort into early (2000–2009) and later (2010–2019) eras to investigate temporal changes.

**Results:** Of 18,046 transplants, 7571 were allogeneic and 10,475 were autologous. Comparing the two eras, allogeneic transplants increased in number by 22.3%, with greater use of matched unrelated donors in the later era. Autologous transplants increased by 10.9%. Temporal improvements in NRM were observed in children and adults. OS improved in pediatric patients and in adults receiving autologous HCT. In adults receiving allogeneic HCT, OS was stable despite the substantially older age of patients in the later era.

**Conclusions:** HCT is an increasingly frequent procedure in Canada which has expanded to serve older adults. Noted temporal improvements in NRM and OS reflect progress in patient and donor selection, preparation for transplant, and post-transplant supportive care. In allogeneic HCT, unrelated donors have become the most frequent donor source, highlighting the importance of the continued growth of volunteer donor registries. These results serve as a baseline measure for quality improvement and health services planning in Canada.

## Abstract 23 (Poster): Letermovir for Secondary Prophylaxis of Cytomegalovirus Infection After Allogeneic Stem Cell Transplantation: A Single Centre Experience (This Abstract Has Subsequently Been Published in Full)

Nihar Desai ^1^, Carol Chen ^1^, Ivan Pasic ^1,2^, Wilson Lam ^1,2^, Arjun Law ^1,2^, Armin Gerbitz ^1,2^, Auro Viswabandya ^1,2^, Dennis D. Kim ^1,2^, Rajat Kumar ^1,2^, Jonas Mattsson ^1,2^, Igor Novitzky-Basso ^1,2^ and Fotios V. Michelis ^1,2^

^1^  Hans Messner Allogeneic Transplant Program, Division of Medical Oncology and Hematology, Princess Margaret Cancer Centre, Toronto, ON, Canada
^2^  Department of Medicine, University of Toronto, Toronto, ON, Canada

**Background:** Letermovir (LMV) is a novel anti-cytomegalovirus (CMV) agent targeting the viral terminase complex. Primary prophylaxis using LMV effectively reduces clinically significant CMV infection after allogeneic hematopoietic cell transplant (allo-HCT). However, there is a paucity of data regarding its efficacy as secondary prophylaxis (SP).

**Purpose:** To report the outcomes of allo-HCT patients receiving LMV as SP at our centre.

**Methods:** Patients receiving LMV as SP at Princess Margaret Cancer Centre between April 2020 and May 2023 were identified from electronic medical records. SP was defined as initiation of LMV at any time after at least one treated episode of CMV post allo-HCT. CMV was monitored weekly, and results were expressed as copies/mL. LMV dose was 480 mg once daily or 240 mg once daily when co-administered with cyclosporine. Baseline variables were summarized using descriptive statistics. Median and range were used for continuous variables, and absolute and percentage frequency were used for categorical variables.

**Results:** Thirty-nine patients received LMV as SP (Table 1). The majority (95%) were CMV seropositive before allo-HCT, while 54% of donors were CMV seronegative. Thirty-two patients (82%) received anti-thymocyte globulin (ATG)-based graft-versus-host disease (GVHD) prophylaxis, with 27 (69%) receiving ATG and post-transplant cyclophosphamide (PTCy).

By definition, all patients had previously received treatment for CMV infection (36 with valganciclovir; six with ganciclovir; two with foscarnet). None had detectable CMV viremia at the time of LMV SP initiation. Eighteen patients (46%) had GVHD at the time of LMV initiation and were receiving immunosuppression.

The median time from allo-HCT to initiation of LMV SP was 47 days (41–56), and the median duration of LMV SP use was 77 days (46–90). A single breakthrough CMV infection occurred and received treatment with valganciclovir. Four CMV infections occurred after LMV SP discontinuation. Reasons for discontinuation of LMV SP included planned completion (77%), CMV infection necessitating treatment (2.6%), and death (17.9%). None of the patients stopped LMV due to adverse events. The median follow-up after discontinuation of LMV SP was 287 days (29–757).

Fifteen patients died during the study period (five relapse; six GVHD; one CMV infection; three sepsis). Seven patients died while they were receiving LMV SP but none of these were linked to LMV.

**Conclusions:** LMV used as SP is effective and well tolerated in this high-risk patient population. Prospective studies on a larger cohort should be performed to validate these findings.

## Abstract 24 (Poster): Early Experience of the First Seven Patients with Sickle Cell Disease Using Non-Myeloablative Conditioning Protocols for HLA-Matched Allogeneic Stem Cell Transplantation

Majed Altareb ^1^, Carol Chen ^1^, Armin Gerbitz ^1,2^, Igor Novitzky-Basso ^1,2^, Arjun Datt Law ^1,2^, Wilson Lam ^1,2^, Ivan Pasic ^1,2^, Fotios V. Michelis ^1,2^, Dennis Dong Hwan Kim ^1,2^, Auro Viswabandya ^1,2^, Jonas Mattsson ^1,2^ and Rajat Kumar ^1,2^

^1^  Hans Messner Allogeneic Transplant Program, Division of Medical Oncology and Hematology, Princess Margaret Hospital, Toronto, ON, Canada
^2^  Department of Medicine, University of Toronto, Toronto, ON, Canada

**Background and Purpose:** Sickle cell disease (SCD) is a hereditary hemoglobinopathy characterized by chronic pain, organ damage, and decreased life expectancy. Allogeneic stem cell transplantation (allo-SCT) has emerged as a curative therapy for SCD. However, most of the experience is centred around pediatric patients and myeloablative conditioning regimens are often recommended in this population with excellent overall survival rates exceeding 90%. Conversely, for adults with SCD, outcomes are not as favorable, and experience is limited. Myeloablative conditioning is not a suitable option for adults due the presence of established organ damage. A novel approach to allo-SCT is non-myeloablative (NMA) conditioning in both children and adults with SCD. However, comprehensive data on NMA conditioning outcomes in adult SCD patients are scarce, especially from Canada. In this study, we explore the feasibility and outcomes employing the chemotherapy-free non-myeloablative conditioning regimen, that included seven adult patients with SCD, aiming to improve safety and reduce treatment-related complications.

**Methods:** We analyzed transplant outcomes among the first seven patients who received HLA-matched allo-SCT for SCD at Princess Margaret Cancer Centre between October 2020 and August 2023.

**Results:** Patient characteristics are shown in Table 1. Seven patients (six males [85.7%]) received HLA-matched allo-SCT for SCD, with median age of 30 years (range 20–48). All patients received frozen peripheral stem cells. The median infused CD34 cell dose was 8.90 × 10^6^ per kg of recipient weight (range 7.81–11.27). All received NMA conditioning with alemtuzumab (0.03 mg/kg on day-7, 0.1 mg/kg on day-6, and 0.3 mg/kg/day on days-5 to -3), and 300 cGy of total body irradiation on Day-2. Sirolimus was started on Day-1, to keep trough levels at 10–15 mcg/L for 3 months, then between 5–10 mcg/L. It was tapered after one year if chimerism was >50%. Platelets were transfused to keep levels > 50 × 10^9^/L and packed red blood cells were transfused to keep Hb > 90 g/L.

With median follow up of 23.7 (range 3–38.03) months, overall survival was 100%. Median neutrophil and platelet engraftment occurred on day 22 (14–24) and 11 (11–23) respectively. Platelet transfusion was not required for four (57%) patients. Median hospitalization period was 33 days (range 27–41). There was low incidence in both acute and chronic GVHD. Only one patient developed grade 1 skin GVHD on day +120 that was treated with topical corticosteroids. There was no chronic GVHD (Figure 1). CMV reactivation necessitating treatment was noted in two patients and four exhibited EBV reactivation but did not require treatment. T-cell and myeloid lineage chimerism is presented in Table 2.

**Conclusions:** Our limited experience shows that this non-myeloablative conditioning protocols is a viable alternative for allo-SCT in SCD. Collaborative efforts across institutions will be instrumental in establishing NMA conditioning as a standard of care in the allo-SCT for adult SCD patients.

## Abstract 25 (Poster): Impact of Co-Administered Acetaminophen on Busulfan Pharmacokinetics

Rutvij A. Khanolkar ^1,2^, Shahbal B. Kangarloo ^1,2^ and Jan Storek ^1,2^

^1^  Cumming School of Medicine, University of Calgary, Calgary, AB, Canada
^2^  Alberta Health Services, Calgary, AB, Canada

**Background:** Although the interaction between acetaminophen and busulfan has been theorized due to overlap in metabolic pathways, no supporting empirical evidence has been reported to date.

**Purpose:** To determine if co-administration of acetaminophen and busulfan will result in an increase busulfan area under the curve (AUC).

**Methods:** We performed a secondary analysis of an open-label, phase II clinical trial (NCT#03456817) of high-dose anti-thymocyte globulin (ATG, Thymoglobulin, 10 mg/kg starting on day-4) compared to standard low-dose ATG controls (4.5 mg/kg ATG starting on day-2). In both high-dose and control patients, other myeloablative conditioning was identical and consisted of busulfan given from days-5 to -2. Prophylaxis of ATG-induced fever was with acetaminophen, which overlapped with busulfan on days-4 to -2 for high-dose ATG patients, but on only day-2 for controls. The study included 56 allogeneic hematopoietic cell transplant (HCT) recipients. On day-8, a test dose of busulfan was administered and pharmacokinetics (PK) determined, based on which the daily busulfan dose for days-5 to -2 was set (targeting total busulfan AUC of 15,000 µM·min). Busulfan daily AUC was determined on days-5 and -2. The primary hypothesis was that the increase from day-5 AUC to day-2 AUC (ΔAUC) would be higher in high-dose ATG patients compared to low-dose ATG controls. Statistical comparison of ΔAUC between groups was conducted using the Mann-Whitney test.

**Results:** The median ΔAUC was significantly higher in high-dose ATG patients (*n* = 9) compared to controls (*n* = 32) (25% vs. 3%, *p* = 0.001). Based on this finding, the trial protocol was amended to give ibuprofen for fever prophylaxis to avoid supratherapeutic busulfan dosing. This resulted in a significantly decrease in ΔAUC for subsequent high-dose ATG patients (Group C; *n* = 15) when compared to the initial nine high-dose ATG patients that received acetaminophen (12% vs. 25%, *p* = 0.034).

**Conclusions:** Acetaminophen likely increases busulfan AUC. This should be taken into consideration when acetaminophen is given to patients undergoing conditioning with busulfan.

## Abstract 26 (Poster): Pharmacokinetic-Targeting and Dose-Adjustment of Intravenous Busulfan for Myeloablative Conditioning in Allogeneic Hematopoietic Cell Transplantation

Rutvij A. Khanolkar ^1,2^, Shahbal B. Kangarloo ^2^, Na Li ^3^, Faisal M. Khan ^4,5^ and Jan Storek ^1,2^

^1^  Cumming School of Medicine, University of Calgary, Calgary, AB, Canada
^2^  Alberta Health Services, Calgary, AB, Canada
^3^  Department of Community Health Sciences, University of Calgary, Calgary, AB, Canada
^4^  Department of Laboratory Medicine & Pathology, University of Calgary, Calgary, AB, Canada
^5^  Alberta Precision Laboratories, Calgary, AB, Canada

**Background:** Pharmacokinetically (PK)-targeting intravenous busulfan improves outcomes following allogeneic hematopoietic cell transplantation (HCT). However, the optimal busulfan area under the curve (AUC) likely differs based on the specific conditioning regimen, and the target busulfan exposure is unknown when combined with fludarabine and low-dose total body irradiation (TBI).

**Purpose:** To determine the optimal busulfan AUC associated with the highest relapse-free survival (RFS) and overall survival (OS).

**Methods:** This study included 1019 adult HCT recipients that received myeloablative conditioning including fludarabine, busulfan, anti-thymocyte globulin, and low-dose (4 cGy) TBI. BU was administered as a total dose of ~3.2 mg/kg given equally from days-5 to -2 pre-transplant. Total AUC was estimated using measurements of serial serum samples. Multivariate Cox and Fine-Gray regression were used for comparison of AUC subgroups.

**Results:** Median AUC was 62.3 mg∙hr/L (range: 39.4–128.0 mg∙hr/L). Total AUC exposure of 49.3–57.5 mg∙hr/L was associated with greater RFS (67% vs. 47%, HR = 1.82, *p* = 0.014) and OS (71% vs. 46%, HR = 1.99, *p* = 0.008) compared to patients with higher AUCs of 57.5–73.9 mg∙hr/L. Although very low (<49.3 mg∙hr/L) or very high (>73.9 mg∙hr/L) AUCs trended towards worse RFS and OS compared to 49.3–57.5 mg∙hr/L, this did not reach statistical significance, possibly due to the limited number of patients with extreme AUCs. Except potentially for patients with a high/very high HCT disease risk index (DRI), 49.3–57.5 mg∙hr/L appeared to be the optimal AUC regardless of patient sex, age, or primary disease.

**Conclusions:** Within the evaluated AUC range, 49.3–57.5 mg∙hr/L appeared to be associated with the most favourable survival. Pharmacokinetic targeting to this range may improve outcomes.

## Abstract 27 (Poster): Proinflammatory Cytokine Release and Infusional Side-Effects After ATG Serotherapy for GVHD Prophylaxis in Allogeneic Hematopoietic Cell Transplantation

Rutvij A. Khanolkar ^1,2^, Stephanie Dookie ^1^, Riley Ngo ^1^, Na Li ^3^, Faisal M. Khan ^4,5^ and Jan Storek ^1,2^

^1^  Cumming School of Medicine, University of Calgary, Calgary, AB, Canada
^2^  Alberta Health Services, Calgary, AB, Canada
^3^  Department of Community Health Sciences, University of Calgary, AB, Canada
^4^  Department of Laboratory Medicine & Pathology, University of Calgary, Calgary, AB, Canada
^5^  Alberta Precision Laboratories, Calgary, AB, Canada

**Background:** Anti-thymocyte globulin (ATG) serotherapy for graft-versus-host disease (GVHD) prophylaxis in allogeneic hematopoietic cell transplantation (HCT) is frequently associated with infusional side-effects (ISEs) including fever, tachycardia, respiratory distress, and hypotension/shock. Although this constellation closely resembles cytokine release syndrome, the mechanism behind ATG ISEs is poorly understood.

**Purpose:** To characterize the frequency and severity of ATG ISEs and determine the association of ISEs with cytokine levels and clinical outcomes.

**Methods:** This study included 190 adult HCT recipients receiving myeloablative conditioning including fludarabine, busulfan, low-dose total body irradiation, and 4.5 mg/kg ATG (0.5 mg/kg on day -2 and 2 mg/kg on days -1 and 0). Patient electronic medical records were retrospectively reviewed for clinical data collection. ISE were classified as thermal (fever/rigors), respiratory (tachypnea/hypoxemia/increased O_2_ supplementation), tachycardia, or circulatory (hypotension/fluid resuscitation requirement). ISEs were further characterized into mild or severe (e.g., mild tachycardia = 105–125 bpm, severe > 125 bpm). Serum levels of 31 cytokines were determined pre- and post-1st ATG infusion (and up to 13 timepoints in select patients) using Luminex (Thermo Fisher Scientific, Waltham, MA, USA). Cox and Fine-Gray regression were used to determine the association of ISEs with clinical outcomes including grade 2–4 acute GVHD (aGVHD), moderate-severe chronic GVHD (cGVHD), cumulative incidence of relapse (CIR), and overall survival (OS).

**Results:** At least one ISE occurred in 79% (151/190) of patients, with a maximum severity of mild in 100 (53%) and severe in 51 (27%). Patients experienced an average of 1.4 types of ISEs (median 1), most commonly tachycardia (57%) and thermal (50%), while respiratory (26%) and circulatory (7%) ISEs were less frequent. ISE frequency was 62%, 60%, and 35% after the 1st–3rd ATG infusions, respectively, with the median time from infusion to first ISE being ~4.5 h. Compared to pre-infusion levels, there was a >1-fold change in 11/34 cytokines (all *p* < 0.001). Of greatest interest was IL-6, which demonstrated a progressive increase in median levels between patients with no vs. mild vs. severe ISEs (*p* < 0.05). There was no association of ISEs with clinical outcomes. Patient age, sex, primary disease, or pre-infusion peripheral blood cell counts (leukocytes, lymphocytes, neutrophils, and monocytes) did not appear to influence ISE frequency or severity.

**Conclusions:** ISEs occur in the majority of ATG recipients, with one-quarter experiencing severe ISEs. The concomitant rise in serum cytokine levels (particularly IL-6) suggests that anti-cytokine antibodies (e.g., tocilizumab) may assist in the prevention or treatment of ISEs without negatively impacting clinical outcomes.

## Abstract 28 (Poster): Outcomes of Allogeneic Stem Cell Transplant in Patients with Multi-Line Relapsed/Refractory Hodgkin Lymphoma

Alejandro Garcia-Horton ^1^, Ravi Bhindi ^2^, Michael Radford ^1^, Tobias Berg ^1,3,4^, Dina Khalaf ^1^, Brian Leber ^5^, Irwin Walker ^5^ and Kylie Lepic ^1^

^1^  Department of Oncology, McMaster University, Hamilton, ON, Canada
^2^  Michael G. DeGroote School of Medicine, Waterloo Regional Campus, McMaster University, Hamilton, ON, Canada
^3^  Centre for Discovery in Cancer Research, McMaster University, Hamilton, ON, Canada
^4^  Escarpment Cancer Research Institute, Hamilton Health Sciences, Hamilton, ON, Canada
^5^  Department of Medicine, McMaster University, Hamilton, ON, Canada

**Background:** Most patients with Hodgkin Lymphoma (HL) are cured with primary therapy with a 5-year overall survival (OS) of 96–99% [1]. However, up to 30% of patients experience treatment failure or disease relapse [2], with historically poor outcomes after autologous stem cell transplant (ASCT). Allogeneic stem cell transplant (alloSCT) is generally reserved as last line of therapy in relapsed/refractory (R/R) HL patients. A CBMTG study from 2010 showed a 2-year OS of 35–65% in Canadian patients [3]. With the introduction of brentuximab and checkpoint inhibitors (nivolumab, pembrolizumab), debate exists as to the benefit of alloSCT in multiple R/R HL patients. Concurrently, alloSCT supportive care and transplant methods have improved, continuing to offer a viable therapy for these patients.

**Purpose:** To investigate outcomes of alloSCT in R/R HL in a heavily pre-treated cohort of patients.

**Methods:** We performed a single-centre, retrospective analysis of R/R HL patients that received an alloSCT between 1 January 2016 and 30 June 2022. Primary endpoint was overall survival (OS). Secondary endpoints included progression-free survival (PFS), non-relapse mortality (NRM), and graft-versus-host disease/relapse-free survival (GRFS). Baseline characteristics and outcomes were extracted. The Kaplan-Meier method was used for survival analysis.

**Results:** Sixteen patients were included, with a median follow-up time of 3.8 years. 50% of the cohort was female and median age was 35 years (range 20–66). All patients received previous ASCT and had a median of four prior lines of therapy (range 3–6). 50% of them were previously exposed to checkpoint inhibitors. 44% of the cohort had an HCT-CI of 1–2 and 25% had a score of 3–4. Six patients had a KPS less than 90%. Haploidentical donor was most common (*n* = 13; 81%) graft source, with three (19%) patients receiving a fully matched unrelated donor transplant. At time of transplantation, 11 patients had obtained a partial response and five had a complete response. 15 patients received conditioning with fludarabine/cyclophosphamide and total body irradiation (Flu/Cy/TBI200) and one with fludarabine and melphalan.

2-year and 5-year OS were 75% and 60%, respectively (Figure 1A). PFS at 2-years was 63% and 5-years 48% (Figure 1B). 1-year and 5-year NRM were 13% and 25%, respectively (Figure 1C). 1-year, 2-year, and 5-year GRFS were 50%, 38%, and 30%, respectively (Figure 1D).

The cohort’s relapse incidence rate was 8.4 per 100 person-years. Grade III–IV acute GVHD occurred in 25% and extensive chronic GVHD in 31% of patients. Six deaths occurred: two were secondary to disease relapse, three from infectious causes (one early death within 100 days of alloSCT), and one from aGVHD.

**Conclusions:** Our outcome data of a single centre, heavily pre-treated cohort of Canadian patients are similar to other recently published cohorts [4]. This data compares favourably to previously published Canadian data [3] and demonstrates that in the era of post-transplant cyclophosphamide containing conditioning, alloSCT is a safe and feasible treatment option for patients with multi-line R/R HL. Prospective studies are required to investigate the role and optimal timing of alloSCT in HL in the era of checkpoint inhibitor use.

**References**

1. Klimm, B.; Goergen, H.; Fuchs, M.; von Tresckow, B.; Böll, B.; Meissner, J.; Glunz, A.; Diehl, V.; Eich, H.T.; Engert, A.; et al. Impact of risk factors on outcomes in early-stage Hodgkin’s lymphoma: an analysis of international staging definitions. *Ann. Oncol.* **2013**, *24*, 3070–3076. https://doi.org/10.1093/annonc/mdt413.
2. Mohty, R.; Dulery, R.; Bazarbachi, A.H.; Savani, M.; Hamed, R.A.; Bazarbachi, A.; Mohty, M. Latest advances in the management of classical Hodgkin lymphoma: The era of novel therapies. *Blood Cancer J*. **2021**, *11*, 126. https://doi.org/10.1038/s41408-021-00518-z.
3. Kuruvilla, J.; Pintilie, M.; Stewart, D.; Lachance, S.; Power, M.; Couture, F.; Xenocostas, A.; Voralia, M.; Couban, S.; Foley, R. Outcomes of reduced-intensity conditioning allo-SCT for Hodgkin’s lymphoma: a national review by the Canadian Blood and Marrow Transplant Group. *Bone Marrow Transplant.* **2009**, *45*, 1253–1255. https://doi.org/10.1038/bmt.2009.321.
4. Montoro, J.; Boumendil, A.; Finel, H.; Bramanti, S.; Castagna, L.; Blaise, D.; Dominietto, A.; Kulagin, A.; Yakoub-Agha, I.; Tbakhi, A.; et al. Post-Transplant Cyclophosphamide-Based Graft-Versus-Host Disease Prophylaxis in HLA-Matched and Haploidentical Donor Transplants for Patients with Hodgkin disease: A Comparative Study of the LWP EBMT. *Blood* **2023**, *142*, 4975–4977. https://doi.org/10.1182/blood-2023-179829.

## Abstract 29 (Poster): Post-Transplant Tyrosine Kinase Inhibitor (TKI) Maintenance in Ph-Positive Acute Lymphoblastic Leukemia Delivers Clinical Benefit Toward Improved Overall, Relapse-Free, GVHD/Relapse-Free Survival and Decreased Non-Relapse Mortality Following Allogeneic Hematopoietic Stem Cell Transplantation

Eshrak Al-Shaiban ^1^, Carol Chen ^1^, Igor Novitzky-Basso ^1,2^, Ivan Pasic ^1,2^, Auro Viswabandya ^1,2^, Rajat Kumar ^1,2^, Wilson Lam ^1,2^, Arjun Law ^1,2^, Armin Gerbitz ^1,2^, Jonas Mattsson ^1,2^, Fotios V. Michelis ^1,2^ and Dennis D.H. Kim ^1,2^

^1^  Hans Messner Allogeneic Transplant Program, Division of Medical Oncology and Hematology, Princess Margaret Hospital, Toronto, ON, Canada
^2^  Department of Medicine, University of Toronto, Toronto, ON, Canada

**Introduction:** Post-allogenic hematopoietic cell transplantation (HCT) maintenance therapy with tyrosine kinase inhibitor (TKI) in Philadelphia-positive acute lymphoblastic leukemia (Ph^+^ ALL) is known to reduce relapse risk, but its clinical benefit remains debated. Herein, we report outcomes of patients (pts) who received post-HCT TKIs maintenance (PTM) versus not in Ph^+^ ALL.

**Methods:** We retrospectively reviewed 80 pts who underwent first-HCT in complete-remission at Princess Margaret Cancer Centre from 2000–2022. Relapse was defined as ≥5% blasts in bone marrow or presence of extramedullary disease. The overall (OS), relapse-free survival (RFS), and graft-versus-host disease (GVHD)/relapse-free survival (GRFS) were calculated by Kaplan-Meier, analyzed by log-rank test. The cumulative-incidence of relapse (CIR), non-relapse mortality (NRM) and chronic GVHD (cGVHD) were calculated using the Fine-Gray model.

**Results:** Patients’ characteristics are summarized in Table 1. After a median follow-up of 26 months among survivors, 16 pts (20%) had progressed post-HCT. Fifty-eight pts (72%) did not receive PTM while 22 pts (28%) received PTM; imatinib (*n* = 15), dasatinib (*n* = 5) or ponatinib (*n* = 4). The median time to start PTM was 5.3 (range; 2.6–9.2) months. ABL1 kinase-domain mutation (KDM) was detected prior-HCT in eight pts, including: T315I (*n* = 2), F317L (*n* = 3), others (*n* = 3).

The analysis for HCT-outcomes showed improved OS, RFS and GRFS and reduced incidence of NRM and cGVHD at 2 years toward the use of PTM, but not on CIR: OS, 81% vs. 43.4%, *p* = 0.01; GRFS, 40% vs. 5.3%, *p* = 0.003; RFS, 81% vs. 43.4% *p* = 0.02; NRM, 4.8% vs. 45.8%, *p* = 0.003; cGVHD 27.7% vs. 41.3%, *p* = 0.54; CIR, 14.3% vs. 10.8%, *p* = 0.19. (Figure 1A–F). Detectable ABL1 KDM pre-HCT and detectable BCR/ABL at day 60 post-HCT increased the CIR (HR: 2.63 [14.28–1.45], *p* = 0.0005). Univariate-analysis showed PTM improved OS (HR; 0.39 [0.19–0.85], *p* = 0.02), RFS (HR: 0.54 [0.29–0.99], *p* = 0.03), GRFS (HR: 0.41 [0.23–0.75], *p* = 0.004) and NRM (HR: 0.045 [0.003–0.20], *p* = 0.003). However, it was not significantly associated with the relapse risk (HR: 0.143 [0.03–0.33], *p* = 0.19). Additionally, age > 40 was associated with improved OS (HR: 2.08 [1.11–3.9], *p* = 0.02), RFS (HR: 1.85 [1.01–3.37], *p* = 0.05) and NRM (HR: 2.44 [3.7–1.81], *p* = 0.04). Time-dependent analysis confirmed that cGVHD associates with improved OS (HR: 0.36 [0.16–0.78], *p* = 0.009), RFS (HR: 0.31 [0.14–0.68], *p* = 0.003) and CIR (HR: 0.1522 [0.04–0.54], *p* = 0.004). Detectable post-HCT BCR/ABL transcript level is associated with increased CIR (HR: 4.17 [12.05–1.45], *p* = 0.009). Multivariable-analysis confirmed that PTM improved OS (HR: 0.28 [0.13–0.62], *p* = 0.02], RFS (HR: 0.32 [0.15–0.68], *p* = 0.003), GRFS (HR: 0.41 [0.23–0.75], *p* = 0.004) and NRM (HR: 0.18 [0.06–0.57], *p* = 0.003).

**Conclusions:** PTM and cGVHD can improve outcomes while detectable BCR/ABL post-HCT is an adverse risk for post-HCT outcomes. PTM was not confirmed to reduce the relapse risk.

## Abstract 30 (Poster): Frailty Impact on Outcomes of Patients Undergoing Chimeric Antigen Receptor T-Cell (CAR-T) Therapy at Princess Margaret Cancer Centre (PMCC): A Prospective Pilot Study

Tiana Coley ^1^, Anca Prica ^1^, Rachel Aitken ^1^, Abi Vijenthira ^1^, Manjula Maganti ^2^, Samantha Mayo ^1^, John Kuruvilla ^1^, Michael Crump ^1^, Sita Bhella ^1^, Robert Kridel ^1^, Vishal Kukreti ^1^, Chloe Yang ^1^, Shabbir Alibhai ^1^ and Christine Chen ^1^

^1^  Division of Medical Oncology and Hematology, Princess Margaret Cancer Centre–University Health Network, Toronto, ON, Canada
^2^  Department of Biostatistics, Princess Margaret Cancer Centre–University Health Network, Toronto, ON, Canada

**Background:** PMCC is one of three adult centres in Ontario, Canada, providing CAR-T therapy as standard of care (SOC) for patients with relapsed and refractory B-cell lymphomas. There is no upper age limit for eligibility, and frailty may be an important factor in assessing fitness for treatment.

**Purpose:** This study aims to determine if frailty assessments pre-CAR T-cell therapy can predict those at higher risk for acute toxicities, progression-free survival (PFS), and overall survival (OS), as well as evaluate changes in frailty over time.

**Methods:** We performed a cohort study of consecutive patients with lymphoma undergoing CAR-T therapy at our institution, from April 2021 to February 2024. Frailty was evaluated using the clinical frailty scale (CFS), grip strength, gait speed, Mini-Cog, Edmonton Symptom Assessment System (ESAS), and Patient Health Questionnaire (PHQ)-2 and PHQ-9 at five time points: baseline and 1, 3, 6 and 12 months (mo) post-CAR T. At baseline, Vulnerable Elders Survey (VES-13), HCT comorbidity index (HCT-CI) and Cumulative Illness Rating Scale (CIRS) were also completed to characterize frailty.

**Results:** Fifty-two patients were included in this analysis. Mean age was 57.9 ± 12.7 years and 54% were male. 50% had *de novo* DLBCL and 24% had transformation from follicular lymphoma. Most patients (83%) received axicabtagene ciloleucel (axi-cel). Median follow-up was 3.80 months (Interquartile range 0.56–24.7 mo). Completed assessments included: 52 at baseline, 44 at 1 mo., 25 at 3 mo., 12 at 6 mo. and 11 at 12 mo. There were clinically significant changes observed in CFS over time (*p* ≤ 0.001), yet no significant changes were observed between timepoints for the physical assessments of grip strength and gait speed. Sixteen patients (31%) experienced ICANS (15% Grade 1) and forty-nine patients (94%) experienced CRS (2% Grade 3, 65% Grade 2) during the 30 days post-cell infusion. The median in-hospital length of stay was 12.5 days (7.0, 99.0) with 7 patients (13%) going to ICU.

There were 21 progression events and 18 deaths (6 without progression); 9 patients remained alive post-progression. On univariable analysis of baseline data, ECOG (2/3), LDH, and VES-13 were predictive of PFS, while ECOG (2/3), LDH, CRP, VES-13, and CFS were predictive of OS. On multivariable analysis of PFS, baseline VES-13 remained significant.

Analyses incorporating repeated measures were performed, and on univariable analysis, CFS, gait speed (>0.8 m/s), LDH, and VES-13 were significantly associated with PFS and the same variables and CRP were associated with OS (Table 1). On multivariable repeated measurements analyses, only the CFS (>3) was clinically significant for PFS and OS, with VES-13 (≥3) also being clinically significant for OS (Table 1).

**Conclusions:** Conducting serial frailty assessments in patients undergoing CAR-T therapy is feasible. CFS is a longitudinal measurement found to significantly change over time, suggesting an element of reversible functional impairment related to patients’ lymphoma. Within the limits of our sample size, baseline measures of frailty were not predictive of development or grade of CRS or ICANS; the relationship of CFS change over time with PFS and OS may be indicative of lymphoma response. Enrollment will be ongoing.

## Abstract 31 (Poster): Outcomes of Allogenic Stem Cell Transplantation on Relapsed/Refractory Classical Hodgkin Lymphoma in the Era of Checkpoint Inhibitors: Insights from a Single-Centre Study

Hadel El-Haddad ^1^, Maryse Power ^1^, Hannah Cherniawsky ^1^, Judith Rodrigo ^1^, Kerry Savage ^2^, Alina Gerrie ^2^, Kevin Song ^1^ and Shanee Chung ^1^

^1^  Leukemia/BMT program of British Columbia, Vancouver General Hospital, Vancouver, BC, Canada
^2^  British Columbia Cancer Centre for Lymphoid Cancer, Vancouver, BC, Canada

**Background:** Patients with relapsed or refractory classical Hodgkin lymphoma (rCHL) who relapse after autologous stem cell transplant (auto-SCT) face a dismal prognosis. Novel agents such as checkpoint inhibitors offer additional treatment strategies in rCHL but are not considered curative. Allogeneic stem cell transplant (allo-SCT) is a potentially curative option; however, its optimal timing in rCHL is unclear. Depth of response pre allo-SCT is a strong predictor of post-transplant outcomes as are patient selection and preparative regimen.

**Purpose:** We sought to evaluate outcomes of patients with rCHL treated with allo-SCT at our centre in the era of salvage immunotherapy, specifically PD-1 inhibitors.

**Methods:** We retrospectively reviewed all patients with rCHL treated with PD1 inhibitors and allo-SCT between May 2021 and November 2021. These dates reflect an institutional policy change to offer allo-SCT to rCHL. Patient, disease and transplant related details were recorded.

**Results:** Five male patients (median age: 37 years, range: 23–49) underwent allo-SCT from May 2021 to November 2021. Four had multiply relapsed CHL and one had primary refractory disease. Patients received a median of 8 (5–11) lines of treatment (Table 1). The four relapsed patients had undergone auto-SCT. Time to relapse post auto-SCT ranged between 2 and 21 months. Prior to allo-SCT, all patients received brentuximab and PD-1 inhibitors (pembrolizumab/nivolumab), and four had received radiation therapy. The best response by PET scan pre-allo-SCT was partial response in four patients and progressive disease in one. The stem cell donors were varied—one matched sibling, two matched unrelated, one mismatched unrelated, and one haploidentical. All patients received a bone marrow graft and reduced-intensity conditioning (RIC; busulfan 3.2 mg/kg daily for two doses, fludarabine 30 mg/m^2^ daily for six doses). Graft-versus-host disease (GVHD) prophylaxis included post-transplant cyclophosphamide (PTCy) and tacrolimus (plus mycophenolate in the haploidentical allo-SCT).

Two patients developed acute GVHD, and one experienced *de novo* chronic GVHD (grade 1 liver involvement). No episode of hyperacute GVHD was noted. All patients relapsed within 1.5 years post allo-SCT (range 7.6 to 17.2 months). Three patients died from disease progression at 8, 12, and 24 months post allo-SCT, two of whom died before post relapse therapy could be administered. Two of five patients remain alive on therapy. Treatment at relapse post-allo-SCT included brentuximab/cyclophosphamide, everolimus, and radiation.

**Conclusions:** Allo-SCT for rCHL after PD1 inhibition with RIC and PTCy was well tolerated in our cohort of five patients, with no patient experiencing non-relapse-mortality or severe GVHD, despite prior exposure to check point inhibitors. However, all relapsed within 18 months of allo-SCT. The poor disease related outcomes in our cohort underscores the challenges associated with patient selection and timing for allo-SCT.

**Abbreviations:** allo-SCT; allogeneic stem cell transplant, auto-SCT; autologous stem cell transplant, DOR; duration of response, GI; gastrointestinal, GVHD; graft-versus-host disease, OS; overall survival from time of allo-SCT.

## Abstract 32 (Poster): Gemtuzumab Ozogamicin in Favorable and Intermediate Risk Acute Myeloid Leukemia: A Single-Centre Experience

Jia Li Liu ^1^, Nushin Sadeghi ^2^, Owen Dan Luo ^1^ and Gizelle Popradi ^3^

^1^  Department of Internal Medicine, McGill University, Montreal, QC, Canada
^2^  Department of Pharmacy, McGill University Health Centre, Montreal, QC, Canada
^3^  Department of Medicine, Division of Hematology, McGill University Health Centre, Montreal, QC, Canada

**Background:** Acute myeloid leukemia (AML) is a common form of leukemia. Gemtuzumab ozogamicin (GO), an anti-CD33 antibody linked to a chemotherapy molecule, demonstrated improved event-free survival (EFS) and overall survival (OS) in favorable and intermediate risk AML when given with standard induction chemotherapy [1–5].

**Purpose:** This retrospective study examined outcomes of AML patients at the McGill University Health Centre (MUHC) who received GO with standard induction chemotherapy compared to induction chemotherapy alone.

**Methods:** We selected favorable and intermediate risk AML patients at the MUHC who received GO with induction chemotherapy from 1 January 2015 to 23 November 2023, with matched controls. Selection was based on the European Leukemia Net AML classification [6], noting that the ALFA study [2] selected favorable patients based on both cytogenetics and NPM1 or CEBPA mutations. We extracted age, sex, length of hospital stays, complete remission (CR), time to neutrophil (absolute neutrophil count (ANC) > 1.0) and platelet (>50 and 100 days) recovery, hepatic toxicity (bilirubin >1.5× upper limit of normal (ULN), transaminases >3× ULN, or veno-occlusive disease (VOD)), stem cell transplantation (SCT), relapse rate, and survival. We performed statistical analysis to obtain hazard ratios (HR) and odds ratios (OR).

**Results:** Our cohort had 16 GO patients and 16 controls (Table 1). There was no increase in hepatic toxicity or longer platelet and neutrophil recovery with GO. Other parameters, including CR and relapse, did not have statistically significant differences (Table 2).

**Conclusions:** GO was well-tolerated with no increase in delayed cell count recovery or hepatic toxicity, in contrast to the ALFA study. There were no significant differences in CR or relapse rates, and there was a trend towards decreased SCT with GO, which was attributed to more non-transplant candidates in the intermediate risk group being selected to receive GO, with the consideration that they would benefit the most. The absence of significant differences may also be due to the small cohort size and short follow-up time compared to ALFA (which had median follow-ups of 24 and 47.6 months). Further evaluation of this cohort will continue clarifying these outcomes.

**Abbreviations:** ANC; absolute neutrophil count, CI; confidence interval, CR; complete remission, GO; gemtuzumab ozogamicin, HR; hazard ratio, LOS; length of stay, OR; odds ratio, Plt; platelet, SCT; stem cell transplantation.

**References**

1. Hills, R.K.; Castaigne, S.; Appelbaum, F.R.; Delaunay, J.; Petersdorf, S.; Othus, M.; Estey, E.H.; Dombret, H.; Chevret, S.; Ifrah, N.; et al. Addition of gemtuzumab ozogamicin to induction chemotherapy in adult patients with acute myeloid leukaemia: a meta-analysis of individual patient data from randomised controlled trials. *Lancet Oncol.* **2014**, *15*, 986–996. https://doi.org/10.1016/s1470-2045(14)70281-5.
2. Lambert, J.; Pautas, C.; Terré, C.; Raffoux, E.; Turlure, P.; Caillot, D.; Legrand, O.; Thomas, X.; Gardin, C.; Gogat-Marchant, K.; et al. Gemtuzumab ozogamicin for *de novo* acute myeloid leukemia: final efficacy and safety updates from the open-label, phase III ALFA-0701 trial. *Haematologica* **2018**, *104*, 113–119. https://doi.org/10.3324/haematol.2018.188888.
3. Castaigne, S.; Pautas, C.; Terré, C.; Raffoux, E.; Bordessoule, D.; Bastie, J.-N.; Legrand, O.; Thomas, X.; Turlure, P.; Reman, O.; et al. Effect of gemtuzumab ozogamicin on survival of adult patients with de-novo acute myeloid leukaemia (ALFA-0701): a randomised, open-label, phase 3 study. *Lancet* **2012**, *379*, 1508–1516. https://doi.org/10.1016/s0140-6736(12)60485-1.
4. Xu, Q.; He, S.; Yu, L. Clinical Benefits and Safety of Gemtuzumab Ozogamicin in Treating Acute Myeloid Leukemia in Various Subgroups: An Updated Systematic Review, Meta-Analysis, and Network Meta-Analysis. *Front. Immunol.* **2021**, *12*, 683595. https://doi.org/10.3389/fimmu.2021.683595.
5. Guo, Y.; Deng, L.; Qiao, Y.; Liu, B. Efficacy and safety of adding gemtuzumab ozogamicin to conventional chemotherapy for adult acute myeloid leukemia: a systematic review and meta-analysis. *Hematology* **2021**, *27*, 53–64. https://doi.org/10.1080/16078454.2021.2013410.
6. Döhner, H.; Wei, A.H.; Appelbaum, F.R.; Craddock, C.; DiNardo, C.D.; Dombret, H.; Ebert, B.L.; Fenaux, P.; Godley, L.A.; Hasserjian, R.P.; et al. Diagnosis and management of AML in adults: 2022 recommendations from an international expert panel on behalf of the ELN. *Blood* **2022**, *140*, 1345–1377. https://doi.org/10.1182/blood.2022016867.

## Abstract 33 (Poster): Organizing Pneumonia as Manifestation of Pulmonary Graft-Versus-Host Disease (GvHD) Responds Better to Treatment than Bronchiolitis Obliterans: A Single-Centre Analysis

Mohammed Abufarhaneh ^1^, Shamim Mortuza ^1^, Marco Mura ^4^, Maurizio Zompatori ^2^, Anargyros Xenocostas ^1,3^ and Uday Deotare ^1,3^

^1^  Division of Hematology, Department of Medicine, University of Western Ontario, London, ON, Canada
^2^  Radiologia, MultiMedica Group, Istituto di Ricovero e Cura a Carattere Scientifico, San Giuseppe Hospital, Milan, Italy
^3^  Blood and Marrow Transplant Program, London Health Sciences Centre, London, ON, Canada
^4^  Division of Respirology, Department of Medicine, University of Western Ontario, London, ON, Canada

**Background:** Allogeneic hematopoietic cell transplant (allo-HCT) is potentially curative for haematological malignant and non-malignant diseases. Survival after allo-HCT has improved over the last few years due to availability of conditioning regimens with variable intensities, better donor and recipient selection, and improved patient care during and after transplant. However, complications can still occur and affect the prognosis. Chronic graft-versus-host disease (GvHD) is a major cause of morbidity and mortality in transplant recipients. Pulmonary GvHD can present as obstructive or restrictive lung disease and has a poor prognosis. The disease can present with variable presentations and response rates.

**Purpose:** To evaluate the serial pulmonary function tests (PFTs) in patients with pulmonary GvHD, evaluate radiological findings and assess treatment response in patients with diagnosed pulmonary GvHD based on phenotypic presentation.

**Methods:** We conducted a retrospective single-centre study for patients who underwent allo-HCT at our centre. We included 22 adult transplant recipients who developed pulmonary GvHD as a part of chronic GvHD. We studied patient’s demographics, transplant characteristics, serial PFTs, radiological findings such as computerised tomography (CT) scans, follow up parameters and mortality. Obstructive disease was defined as ratio of forced expiratory volume in one second (FEV1) to forced vital capacity (FVC) < 0.7. Restrictive lung disease was defined as either FEV1/FVC ratio of >0.7 and FVC < 80% or diffusing capacity of the lungs for carbon monoxide (DLCO) < 70%. Lung disease type was phenotypically characterized based on patient’s PFTs and CT findings. The three categories which were distinctly studied were bronchiolitis obliterans syndrome (BOS), organizing pneumonia (OP), and an intermediate mixed/discordant pattern between CT scan findings and PFTs. We then subsequently assessed the PFTs every 4 months to evaluate treatment response over a period of 48–60 months.

**Results:** Of the 22 patients, median age was 60 years and female patients were 12 (55%). Most patients had acute myeloid leukemia as their primary disease. Patients received variable induction regimens, conditioning chemotherapy and GvHD prophylaxis. Median time from transplant to development of GvHD was 16 months. Of 22 patients, 12 patients (55%) were diagnosed with BOS, 6 patients (27%) with OP and 4 patients (18%) with mixed pattern. Based on the serial PFTs in the first 48 months after transplant, the disease was classified based on mean FEV1 and mean FVC by phenotype. Our results over time indicated that patients with OP tended to response better to treatment and had better lung function as compared to patients with BOS (Figure 1). However, patients with BOS showed initial decline and then stability over time. The survival probability was not statistically significant between the three phenotypically variable groups (Figure 2).

**Conclusions:** This retrospective study assessed serial PFTs and radiological findings in patients with pulmonary GvHD, categorizing into three distinct groups. BOS was more common than OP, but OP tended to respond better over time with treatment as compared to BOS, with more sustainable improvement in their lung function over time.

## Abstract 34 (Poster): Using Routine Asymptomatic C. Difficile Testing to Identify Patients at High Risk of Developing C. Difficile Infection During Hematopoietic Cell Transplantation

Nicole Janusz ^1,2^, Leanne Mortimer ^3^, Tamara Leite ^4^, Amanda Carroll ^4^, Natasha Kekre ^1,2,4^, Michael Kennah ^1,2^, Austin Yan ^1,2^, Jaxon Senechal ^2^, *C. Arianne Buchan ^1,2,4^, and *Derek MacFadden ^1,2,3^

*Co-PIs

^1^  The Ottawa Hospital, Ottawa, ON, Canada
^2^  Department of Medicine, University of Ottawa, Ottawa, ON, Canada
^3^  The Eastern Ontario Laboratory Association, Ottawa, ON, Canada
^4^  Ottawa Hospital Research Institute, Ottawa, ON, Canada

**Background:** Colonization with *C. difficile* is associated with subsequent *C. difficile* infection (CDI), particularly among patients undergoing hematopoietic cell transplant (HCT), leading to increased morbidity and mortality. Universal use of oral vancomycin in HCT patients as CDI prophylaxis is not endorsed by current guidelines, however targeted prophylaxis may be of benefit to those patients identified at high risk of developing CDI.

**Purpose:** To evaluate the utility of existing *C. difficile* stool detection methods used in pre-transplant planning for identifying individuals at high risk for developing CDI.

**Methods:** We performed a prospective cohort study on patients undergoing HCT. Stool samples were collected at various stages during the transplant process, including a baseline sample used to evaluate for *C. difficile* colonization using a routine 2-step glutamine dehydrogenase (GDH) enzyme immunoassay screen, followed by a toxin B gene (*tcdB*) PCR on GDH^+^ samples. We performed chart review spanning from pre-transplant assessment to three months post-transplant. We calculated test characteristics associated with using baseline *C. difficile* colonization as a screening test for predicting subsequent CDI.

**Results:** The prevalence of *C. difficile* colonization among patients undergoing HCT was 10% (6/60). None of the colonized patients reported symptoms of *C. difficile* at baseline. A total of 10 patients developed CDI within 3 months of transplant. Of these 10 patients, 50% were colonized at baseline. Of all colonized patients, 83% (5/6) developed CDI over the course of their follow up period. Of the 10 patients who developed CDI, all had new onset diarrhea during their admission to hospital. All patients who developed CDI were treated with oral vancomycin for at least 10 days, and all reported complete resolution of their symptoms following treatment. The overall sensitivity and specificity of using *C. difficile* colonization (GDH positive +/− *tcdB* PCR positive) as a screening test for identifying patients at risk of developing CDI was 50% (95% CI, 19–81%) and 98% (95% CI, 89–100%). The positive and negative predictive values were 83% (95% CI, 36–100%) and 91% (95% CI, 80–97%).

**Conclusions:** We identified a subgroup of HCT patients at high risk for development of *C. difficile* infection during their peri-transplant period. This approach, using existing clinical tools, could support implementation of targeted CDI prophylaxis in patients undergoing HCT.

## Abstract 35 (Poster): Efficacy and Safety of Brexucabtagene Autoleucel CAR T-Cell Therapy with BTK Inhibitors in Relapsed Mantle Cell Lymphoma with Central Nervous System Involvement (This Abstract Has Subsequently Been Published in Full)

Anath C. Lionel ^1^, Ashwath Gurumurthi ^1^, Ahmed Fetooh ^1^, Rami Eldaya ^2^, Sairah Ahmed ^1^, Swaminathan P. Iyer ^1^, Loretta J. Nastoupil ^1^, Jason Westin ^1^, Ranjit Nair ^1^, Luis Fayad ^1^, Luis Malpica ^1^, Sudhakar Tummala **^3^**, Christopher Flowers ^1^, Sattva S. Neelapu ^1^, Michael L. Wang ^1^ and Preetesh Jain ^1^

^1^  Department of Lymphoma and Myeloma, The University of Texas MD Anderson Cancer Center, Houston, TX, United States of America
^2^  Department of Neuroradiology, The University of Texas MD Anderson Cancer Center, Houston, TX, United States of America
^3^  Department of Neuro-oncology, The University of Texas MD Anderson Cancer Center, Houston, TX, United States of America

**Background:** Brexucabtagene autoleucel (brexu-cel) is an anti-CD19 CAR T-cell (CAR-T) therapy approved for use in relapsed or refractory mantle cell lymphoma (RR-MCL) after the pivotal ZUMA-2 trial. Central nervous system (CNS) involvement of MCL is infrequent (<5% of cases) and typically has a poor prognosis. Although ZUMA-2 excluded patients with CNS relapse of MCL, there is emerging evidence for the efficacy of brexu-cel in such patients. While findings from the TARMAC trial suggest that BTK inhibitors (BTKi) may improve efficacy of CAR-T in MCL, the impact of concurrent treatment with BTKi and brexu-cel in patients with CNS relapse of MCL is unclear.

**Purpose:** This study examined the efficacy and safety of the use of BTK inhibitors as bridging therapy prior to brexu-cel and as maintenance treatment following brexu-cel.

**Methods:** We describe four patients with RR-MCL and secondary CNS disease. Each patient had one or more markers of high-risk disease such as complex karyotype, TP53 mutation or Ki-67 index > 30%. All patients had been heavily pre-treated, with median prior lines of treatment of four, and had disease that was either refractory to first-line therapy or progressed despite prior BTKi therapy with ibrutinib or acalabrutinib. Patients were assessed for CNS and systemic disease with CSF testing and PET/CT scans prior to brexu-cel infusion and for response to therapy at days +30 and +90. Toxicities were graded using the ASTCT criteria.

**Results:** All four patients had remission of CNS disease at day +30 assessment. The overall complete remission (CR) rate was 75% with one of the four patients experiencing systemic relapse at day +30; the other 3 cases continued to be in durable remission, with median follow-up time of 10 months. These outcomes compare favorably with reported median overall survival of less than 5 months for patients with CNS relapse of MCL, in the pre-brexu-cel era. Our observation of complete resolution of CNS disease in all patients at day +30 compared favorably with findings from the recent case series by Ryan et al. [1] in which only 2 of 7 patients had complete CNS response at day +30 after use of brexu-cel alone without BTKi.

In the 4 patients in this case series, there were no therapy related deaths, infectious complications or severe CRS ≥ grade 3. Two of the four patients had ICU admissions related to grade 3 neurotoxicity, which responded to corticosteroid therapy. These toxicity findings are in line with frequencies of ≥grade 3 CRS and neurotoxicity of 8% and 32% respectively in real-world brexu-cel data. Case 1 had an episode of atrial fibrillation during ICU admission for ICANS; there were no reported arrhythmias in the other three cases while on BTKi maintenance following brexu-cel.

**Conclusions:** In summary, our findings suggest that in patients with RR-MCL and CNS disease, the use of BTK inhibitors as bridging therapy prior to brexu-cel, and as maintenance treatment following brexu-cel, can be efficacious and safe without excess toxicities.

**Reference**

1. Ryan, C.E.; Zon, R.L.; Redd, R.; Fisher, D.C.; Shouval, R.; Kumar, A.; Crombie, J.L.; Sadrzadeh, H.; Kim, A.I.; Nayak, L.; et al. Clinical efficacy and safety of chimeric antigen receptor T-cell therapy for mantle cell lymphoma with secondary central nervous system involvement. *Br. J. Haematol.* **2023**, *203*, 774–780. https://doi.org/10.1111/bjh.19037.

## Abstract 36 (Oral): Exagamglogene Autotemcel for Transfusion-Dependent β-Thalassemia and Severe Sickle Cell Disease (Award Recipient—Clinical Trials/Observations)

Amanda M. Li ^1^, Haydar Frangoul ^2^, Franco Locatelli ^3^, Roland Meisel ^4^, Akshay Sharma ^5^, Monica Bhatia ^6^, Markus Mapara ^7^, Lyndsay Molinari ^8^, Donna Wall ^9^, Robert I. Liem ^10^, Paul Telfer ^11^, Ami J. Shah ^12^, Selim Corbacioglu ^13^, Peter Lang ^14^, Josu de la Fuente ^15^, Marina Cavazzana ^16^, Ben Carpenter ^17^, Janet L. Kwiatkowski ^18^, Maria Domenica Cappellini ^19^, Mattia Algeri ^3^, Antonis Kattamis ^20^, Sujit Sheth ^21^, Damiano Rondelli ^22^, Laurence Dedeken ^23^, Stephan Lobitz ^24^, Mariane de Montalembert ^25^, Martin Steinberg ^26^, Mark C. Walters ^27^, Christopher Simard ^28^, Fengjuan Xuan ^28^, Phuong Khanh Morrow ^29^, Bill Hobbs ^28^ and Stephan A. Grupp ^30^

^1^  British Columbia Children’s Hospital, Vancouver, BC, Canada
^2^  Sarah Cannon Center Research Institute at The Children’s Hospital at TriStar Centennial, Nashville, TN, United States
^3^  Istituto di Ricovero e Cura a Carattere Scientifico, Ospedale Pediatrico Bambino Gesù Rome, Catholic University of the Sacred Heart, Rome, Italy
^4^  Division of Pediatric Stem Cell Therapy, Department of Pediatric Oncology, Hematology and Clinical Immunology, Heinrich-Heine-University, Duesseldorf, Germany
^5^  Bone Marrow Transplantation and Cellular Therapy, St. Jude Children’s Research Hospital, Memphis, TN, United States
^6^  Department of Pediatrics, Columbia University Irving Medical Center, New York, NY, United States of America
^7^  Department of Medicine, Division of Hematology/Oncology, Columbia University, New York, NY, United States of America
^8^  Sarah Cannon Pediatric Transplant and Cellular Therapy Program at Methodist Children’s Hospital, San Antonio, TX, United States of America
^9^  The Hospital for Sick Children, Toronto, Canada
^10^  Ann & Robert H. Lurie Children’s Hospital of Chicago, Chicago, IL, United States of America
^11^  Royal London Hospital, Barts Health National Health Service Trust, London, United Kingdom
^12^  Stanford University, Palo Alto, CA, United States of America
^13^  Department of Pediatric Hematology, Oncology, and Stem Cell Transplantation, University of Regensburg, Regensburg, Germany
^14^  Department of General Paediatrics, University of Tübingen, Tübingen, Germany
^15^  Imperial College Healthcare NHS Trust, St Mary’s Hospital, London, United Kingdom
^16^  Necker-Enfants Malades Hospital, Assistance Publique-Hôpitaux de Paris (AP-HP), University of Paris, Paris, France
^17^  University College London Hospitals National Health Service Foundation Trust, London, United Kingdom
^18^  Perelman School of Medicine, University of Pennsylvania, Philadelphia, PA, United States of America
^19^  Department of Internal Medicine, University of Milan, Milan, Italy
^20^  National and Kapodistrian University of Athens, Athens, Greece
^21^  Joan and Sanford I Weill Medical College of Cornell University, New York, NY, United States of America
^22^  Division of Hematology and Oncology, University of Illinois at Chicago, Chicago, IL, United States of America
^23^  Hopital Universitaire des Enfants Reine Fabiola, Brussels, Belgium
^24^  Gemeinschaftsklinikum Mittelrhein, Koblenz, Germany
^25^  Necker-Enfants Malades Hospital, Assistance Publique-Hôpitaux de Paris, University of Paris-Cité, Paris, France
^26^  Chobanian & Avedisian School of Medicine, Boston University, Boston, MA, United States of America
^27^  UCSF Benioff Children’s Hospital, Oakland, CA, United States of America
^28^  Vertex Pharmaceuticals Incorporated, Boston, MA, United States of America
^29^  CRISPR Therapeutics, Cambridge, MA, United States of America
^30^  Children’s Hospital of Philadelphia, Philadelphia, PA, United States of America

**Background:** Exagamglogene autotemcel (exa-cel) is a non-viral cell therapy designed to reactivate fetal hemoglobin synthesis via *ex vivo* CRISPR-Cas9 gene-editing of autologous CD34+ hematopoietic stem and progenitor cells.

**Methods:** CLIMB THAL-111 and CLIMB SCD-121 are ongoing, 24-month, phase III trials of exa-cel in patients (pts) aged 12–35 with: (i) transfusion-dependent β-thalassemia (TDT) (CLIMB THAL-111; primary efficacy endpoint proportion of pts maintaining weighted average hemoglobin (Hb) ≥ 9 g/dL without red blood cell (RBC) transfusion for ≥12 consecutive months [TI12]) or (ii) severe sickle cell disease (SCD) (CLIMB SCD-121; primary efficacy endpoint proportion of pts free of severe vaso-occlusive crises (VOCs) for ≥12 consecutive months [VF12]).

**Results:** 52 pts with TDT (data cut 16 January 2023) and 44 pts with SCD (data cut 14 June 2023) received exa-cel. Following infusion, all pts engrafted neutrophils and platelets (median 29 and 44 days [TDT] and 27 and 35 days [SCD], respectively). 32/35 (91.4%) pts with TDT evaluable for primary endpoint achieved TI12 (95% confidence interval (CI) width: 76.9–98.2%; *p* < 0.0001); pts achieving TI12 stopped transfusions 35.2 (+/− 18.5) days after infusion and remained transfusion independent for 22.5 (range 13.3–45.1) months. For all pts with TDT, mean total Hb was 11.4 g/dL at month 3 (≥12 g/dL at month 6 onward) and mean HbF was 7.7 g/dL at month 3 (≥10 g/dL at month 6 onward) with pancellular distribution (≥94% RBCs expressing HbF month 6 onward). 29/30 (96.7%) pts with SCD evaluable for primary endpoint achieved VF12 (95% CI, 82.8–99.9%; *p* < 0.0001); mean VOC-free duration 22.4 (range 14.8–45.5) months. For all pts with SCD, mean total Hb was 11.9 g/dL at month 3 and was maintained at normal or near normal levels; mean HbF was 36.9% at month 3 and generally ≥40.0% from month 6, with pancellular distribution. In both trials, proportions of edited *BCL11A* alleles were stable in bone marrow CD34^+^ and peripheral blood nucleated cells. All pts had adverse events (AEs), most Grade 1 or 2; 46 (88.5%) of pts with TDT and 42 (95.5%) with SCD also had AEs of Grade 3 or 4 severity. Most AEs and serious AEs occurred within the first six months and were generally consistent with myeloablative busulfan conditioning and autologous transplantation. One pt with SCD died from respiratory failure due to COVID-19 unrelated to exa-cel. There were no malignancies.

**Conclusions:** These results suggest exa-cel has the potential to deliver a one-time functional cure to pts with TDT or severe SCD.

## Abstract 37 (Poster): Real-World Use of Tafasitamab in the Management of Relapsed or Refractory Diffuse Large B-Cell Lymphoma in a Canadian Patient Support Program

Winson Cheung ^1^, Philip Ding ^1^, Karen Turpin ^2^ and Caroline Koch ^2^

^1^  Oncology Outcomes, University of Calgary, Calgary, AB, Canada
^2^  Incyte Biosciences Canada, Pointe-Claire, QC, Canada

**Background:** Approximately 40% of patients with diffuse large B-cell lymphoma (DLBCL) are refractory to or will relapse (r/r) after first line therapy. Of these patients, about 50% are eligible to receive the standard of care, autologous stem cell transplant (ASCT). For those who are ineligible for ASCT, there is no current standard of care for second line (2L) therapy in Canada. Treatments used in the 2L+ setting report median overall survival durations of 6–10 months. For subsequent treatment options, effective shared decision making is critical and is best informed by real world data together with emerging clinical trial findings. Tafasitamab (tafa), a CD19-targeting immunotherapy, received accelerated approval by the Food and Drug Administration and Health Canada Notice of Compliance with conditions approval and National Comprehensive Cancer Network endorsement for use in combination with lenalidomide for adult patients with r/r DLBCL who are not eligible for ASCT.

**Purpose:** We sought to describe the real-world use of tafa in the management of patients with r/r DLBCL in a Canadian patient support program (PSP).

**Methods:** We conducted a retrospective cohort study of eligible patients with r/r DLBCL who enrolled in the IncyteSolutions PSP to receive tafa between November 2021 and July 2023. Study data were collected from program enrollment and follow-up, which consisted of demographic, clinical, and treatment information, among those patients who consented to use of their data for research purposes. Treatment duration was estimated using the Kaplan-Meier method.

**Results:** 96 patients enrolled in the PSP, consented, and received one or more doses of tafa by the July 2023 data cut-off. They were enrolled by 73 physicians across 43 healthcare institutions. The median age was 76 years (range 37–93) and 44% were female. Similar proportions of patients received tafa as 2L, 3L, and 4L+ therapy (34%, 31%, and 34%, respectively). These patients spanned eight provinces, but most resided in either Ontario (48%) or Québec (32%). Median follow-up time was 2.1 months (Interquartile range 0.7–4.9). Median treatment duration of 2L, 3L, and 4L+ tafa was 4.8 months (95% CI, 2.6–NR), 2.8 months (95% CI, 2.1–11.8), and 1.6 months (95% CI, 0.9–3.3) respectively. Among the 64 patients who discontinued tafa, the most common reasons were physician decision (28%), death (22%), adverse event (19%), and disease progression (16%). At data cut-off, 32 patients remained on tafa treatment, of whom 15 (47%) were in 2L (Figure 1).

**Conclusions:** Tafa for r/r DLBCL management facilitated by this Canadian PSP was used across Canada. While the phase II L-MIND trial reported superior outcomes from tafa use in 2L versus 3/4L, 34% of this cohort received tafa as 2L and many patients remain on treatment. Further Canadian research is underway to examine the treatment patterns and clinical outcomes of tafasitamab/lenalidomide combination therapy in r/r DLBCL.

## Abstract 38 (Oral): Axatilimab for Chronic Graft-Versus-Host Disease: Responses in Fibrosis-Dominant Organs in AGAVE-201 (Award Recipient—Clinical Trials/Observations)

Jennifer White ^1^, Corey Cutler ^2^, Zachariah DeFilipp ^3^, Stephanie J. Lee ^4^, Wendy Ingram ^5^, Helene Schoemans ^6,7^, Laetitia Souchet ^8^, Daniel Wolff ^9^, Carrie L. Kitko ^10^, Simona Sica ^11,12^, Avichai Shimoni ^13^, Britnie Thomas ^14^, Vedran Radojcic ^15^, Chuan Tian ^14^, Amandeep Salhotra ^16^ and Jose A. Perez-Simon ^17^

^1^  British Columbia Cancer Agency, Vancouver General Hospital, Vancouver, BC, Canada
^2^  Dana-Farber Cancer Institute, Boston, MA, United States of America
^3^  Massachusetts General Hospital, Boston, MA, United States of America
^4^  Fred Hutchinson Cancer Center, Seattle, WA, United States of America
^5^  University Hospital of Wales, Cardiff, United Kingdom
^6^  Department of Hematology, University Hospitals Leuven, Leuven, Belgium
^7^  Department of Public Health and Primary Care, Katholieke Universiteit Leuven, Leuven, Belgium
^8^  Clinical Hematology Unit, Groupe Hospitalier Pitié-Salpêtrière, Hôpitaux de Paris Sorbonne Université, Paris, France
^9^  University Hospital of Regensburg, Regensburg, Germany
^10^  Vanderbilt University Medical Center, Nashville, TN, United States of America
^11^  Dipartimento di Diagnostica per Immagini, Radioterapia Oncologica ed Ematologia, Istituto di Ricovero e Cura a Carattere Scientifico, Rome, Italy
^12^  Università Cattolica Sacro Cuore, Rome, Italy
^13^  Sackler Medical School, Tel-Aviv University, Tel-Aviv, Israel
^14^  Incyte Corporation, Wilmington, DE, United States of America
^15^  Syndax Pharmaceuticals, Inc, Waltham, MA, United States of America
^16^  City of Hope Medical Center, Duarte, CA, United States of America
^17^  Consejo Superior de Investigaciones Científicas, Hospital Universitario Virgen del Rocío Instituto de Biomedicina de Sevilla, Seville, Spain

**Background:** Chronic graft-versus-host disease (cGVHD) is a major cause of morbidity after allogeneic hematopoietic stem cell transplantation. Colony-stimulating factor 1 receptor (CSF-1R)-dependent monocytes and macrophages potentiate inflammation and fibrosis, key processes leading to multiorgan damage. Axatilimab (anti–CSF-1R monoclonal antibody) was evaluated in a pivotal, phase II, open-label, randomized study (AGAVE-201; NCT04710576).

**Purpose:** To evaluate fibrosis-dominant organ-specific responses and related changes in patient (pt)-reported symptom burden. Safety was also examined, with a focus on infections.

**Methods:** Study details were reported previously. Organ-specific responses were measured using 2014 National Institutes of Health cGVHD consensus criteria. Modified Lee Symptom Scale organ subdomains were analyzed to identify changes in fibrosis-associated symptom burden.

**Results:** Across all cohorts (*n* = 241), 193 pts (80%) had skin involvement (93% of whom had sclerotic skin), 162 (67%) had joint/fascia involvement, 108 (45%) had lung involvement, and 61 (25%) had esophageal involvement. Responses were seen in all involved organs, including complete responses (CRs). Notable efficacy was documented in fibrosis-dominant organs across doses with highest responses in the 0.3 mg/kg every 2 weeks (Q2W) cohort (Table 1). Median (range) time to response for fibrosis-dominant organs was concordant with time to overall response, except for slight prolongation for skin (0.3-mg/kg cohort, 3.7 [1.0–8.4] mo.) and lungs (0.3-mg/kg cohort, 2.9 [1.0–7.2] mo.); median (range) time to response for joints/fascia and esophagus in the 0.3-mg/kg cohort were 1.9 (1.0–9.5) and 1.9 (0.9–10.5) mo., respectively. Lung responses (47% in 0.3-mg/kg cohort), including CR, were based on both forced expiratory volume in 1 s and symptom score improvement. Skin responses were accompanied by reductions in sclerotic body surface area in almost half of pts (46% overall), and clinician-reported skin and joint tightening improvement was documented in 61% of overall pts. A ≥ 2-point improvement in photographic range of motion score was recorded in all cohorts and was highest with the 0.3-mg/kg dose (55%). Most pts with thickened skin symptoms reported improvement (72% overall). Overall, adverse events were mostly low grade, reversible, and increased with higher doses, with no new safety signals. No cytomegalovirus or Epstein–Barr virus infections (including reactivations) or invasive fungal infections occurred in the 0.3-mg/kg cohort; each of these events was infrequent with higher doses (1–4 pts per cohort). COVID-19 occurred in 44 pts overall (18%), with frequency similar between cohorts and no fatal cases.

**Conclusions:** In AGAVE-201, clinical activity in fibrosis-dominant organs is supported by clinician-reported changes in most pts and pt-reported reductions in organ-specific symptom burden. Axatilimab was generally well tolerated; opportunistic infections were infrequent.

## Abstract 39 (Poster): Real World Impact of the Addition of Anti-Thymocyte Globulin to Standard Graft-Versus-Host Disease (GVHD) Prophylaxis on Myeloablative Unrelated Donor Transplants: Important Reductions in Acute and Chronic GVHD and Trade-Off of GVHD-Related Mortality for Relapse Mortality

Ni Bai ^1^, Wasithep Limvorapitak ^2^, Rob Henderson ^1^, Yasser Abou Mourad ^1^, Shanee Chung ^1^, Donna Forrest ^1^, Kevin Hay ^1^, Florian Kuchenbauer ^1^, Stephen Nantel ^1^, Sujaatha Narayanan ^1^, Thomas Nevill ^1^, Maryse Power ^1^, Judith Rodrigo ^1^, Claudie Roy ^1^, David Sanford ^1^, Kevin Song ^1^, Ryan Stubbins ^1^, Heather Sutherland ^1^, Cynthia Toze ^1^ and Jennifer White ^1^

^1^  Department of Medicine, Leukemia Bone Marrow Transplant Program of British Columbia, University of British Columbia, Vancouver, BC, Canada
^2^  Department of Internal Medicine, Division of Hematology, Thammasat University, Rangsit Campus, Pathumthani, Thailand

**Background:** Graft-versus-host disease (GVHD) is the leading cause of non-relapse mortality (NRM) for HSCT recipients. Several randomized controlled trials have demonstrated a reduction in the incidence of both acute and chronic GVHD when adding anti-thymocyte globulin (ATG) as GVHD prophylaxis regimen. However, it remains controversial whether these gains are offset by an increase in relapse.

**Purpose:** We conducted a real-world retrospective historical comparison study to examine the effects of ATG on the rate of acute and chronic GVHD, cumulative incidence of relapse, mortality, and survival outcomes, in a uniform setting of clinical practice.

**Methods:** Patients who underwent myeloablative allogeneic hematopoietic stem cell transplantation (HSCT) in our program during 1 January 2014 to 31 December 2020 were included. The ATG group was composed of patients who had HSCT from 1 January 2016 to 31 December 2020, and was compared to patients who had transplant from 1 January 2014 to 31 December 2015 (control). All subjects had a minimum of 2-year follow-up.

**Results:** 210 HSCT recipients were included in the study. 140 patients received ATG + cyclosporine and methotrexate (CSA/MTX), and the comparison group (70 patients) received CSA/MTX only. The incidence of all grade acute GVHD was significantly lower in the ATG group than the control (51.4% vs. 70.0%, *p* = 0.010). The number of patients with grade 3 or 4 acute GVHD was also lower with ATG (10.7% vs. 24.3%, *p* = 0.010). The incidence of all grade chronic GVHD was significantly lower in the ATG group than the control at both 1-year (36.4% vs. 62.9%, *p* < 0.001) and 2-years (40.0% vs. 65.7%, *p* < 0.001) post-HSCT. NIH moderate or severe chronic GVHD occurred less frequently with ATG, compared with control (22.8% vs. 55.7%, *p* < 0.001).

The cumulative incidence of relapse at 2-years post-HSCT was higher in the ATG group though this result was not statistically significant (31.4% vs. 17.1%, *p* = 0.216). However, there was a proportional increase in relapse-related deaths in the ATG group. The cumulative incidence of non-relapse mortality was higher in the control than the ATG group (22.9% vs. 11.4%, *p* = 0.066), and the mortality due to GVHD was significantly higher in the control than the ATG group (18.5% vs. 4.3%; *p* = 0.024). However, the 2-year severe GVHD-relapse-free survival was significantly higher in the ATG group than the control (36.4% vs. 12.9%; *p* < 0.001). Nevertheless, the 2-year overall survival was similar between the two groups (ATG: 65.0% vs. control: 64.3%).

**Conclusions:** Our results further confirm the effectiveness of ATG in prevention of acute and chronic GVHD in the real-world setting. An important result from our study is the equalization of overall survival between the ATG and control groups at one and two years. The implication suggested by this study is that GVHD-associated mortality which tends to occur earlier, is offset by later relapse mortality producing similar overall survival over time.

## Abstract 40 (Poster): Real-World Experience of FLT3-Inhibitor Post-Transplant Maintenance with Sorafenib Demonstrating Superior Overall- and Relapse Free Survival Following Allogeneic Hematopoietic Stem Cell Transplantation in Acute Myeloid Leukemia with FLT3-ITD

Yomna Eissa, Eshrak Al-Shaibani, Igor Novitzky-Basso, Ivan Pasic, Wilson Lam, Arjun D. Law, Fotios Michelis, Auro Viswabandya, Armin Gerbitz, Jose-Mario Capo-chichi, Rajat Kumar, Jonas Mattsson and Dennis D. H. Kim

Princess Margaret Cancer Centre, Toronto, ON, Canada

**Background:** Acute myeloid leukemia (AML) with FLT3-internal tandem duplication (ITD) is known to have a significantly high risk of relapse even with allogeneic stem cell transplantation (HCT), for which post-transplant maintenance therapy with FLT3 inhibitors (FLT3i) has been applied. Although there is a randomized trial comparing sorafenib maintenance vs. no treatment, real-world experience is still limited, and the optimal dose and duration of post-transplant sorafenib maintenance remains uncertain.

**Methods:** We conducted a retrospective study including 69 patients with FLT3-ITD positive AML, who received an allogeneic HCT from 2019 to 2023. Of those, 24 patients (35%) received sorafenib maintenance post-HCT.

The primary endpoint was relapse-free survival (RFS). Kaplan-Meier (KM) and Mantel-Byar tests (MBT) were conducted to compare outcomes between the sorafenib maintenance group vs. others. FLT3-ITD allele frequency (AF) was assessed by molecular PCR test, and the cutoff AF of 54.6% was detected.

**Results:** Sorafenib was started in 24 patients at a median of 95 days post-HCT, at a dose of 200 mg every other day (*n* = 2), 200 mg once daily (OD) (*n* = 17) or 200 mg twice daily (BID) (*n* = 5). The maintenance doses used were 200 mg OD (*n* = 9), 200 mg BID (*n* = 6), 200 mg every other day (*n* = 5), and 400 mg BID (*n* = 1). The median sorafenib maintenance duration was 15 months at the last follow-up. Sorafenib was discontinued in 6 patients, of which 2 relapsed, 1 died of an unrelated cause, 1 developed GVHD, 1 pneumonitis and 1 recurrent hypoglycemia. Adverse events requiring sorafenib dose reduction included, GI intolerance (*n* = 7), skin rash (*n* = 7), thrombocytopenia (*n* = 4), transaminitis (*n* = 3), neuropathy (*n* = 3), GVHD (*n* = 2) and pneumonitis (*n* = 2).

The median follow-up duration for all was 27 months following HCT, or 13 months following Sorafenib maintenance. Using the KM test to compare patients who received Sorafenib maintenance vs. no Sorafenib, the 2-year RFS was 84.5% vs. 40.5% (*p* = 0.0005), the 2-year overall survival (OS) was 88.2% vs. 53.9% (*p* = 0.004), and the cumulative incidence of relapse (CIR) was 9.8% vs. 39.9% (*p* = 0.006), respectively, with no increase in non-relapse mortality (NMR). In the MBT, Sorafenib maintenance reduced the mortality risk by 62.5% (HR 0.375, *p* = 0.120) and reduced the relapse/mortality risk by 71.2% (HR 0.288, *p* = 0.047).

When evaluating RFS and OS with combined risk incorporating the use of Sorafenib and FLT3-ITD AF, we have 4 subgroups. In patients with a lower AF, the RFS was 87.7% vs. 56.8% in the Sorafenib vs. no Sorafenib group, respectively. In patients with a higher AF, the RFS was 66.7% vs. 0.0% in the Sorafenib, vs. no Sorafenib group (*p* = 0.001), respectively (Figure 1). The RFS benefit was confirmed in a multivariate analysis.

**Conclusions:** Sorafenib maintenance post-HCT in FLT3-ITD positive AML patients significantly improves the OS and RFS, particularly in patients with higher FLT3-ITD AF at initial diagnosis, defined by ≥54.6% using PCR. Reduced doses of sorafenib improve tolerability, with no significant impact on increased risk of relapse.

## Abstract 41 (Poster): Improvements in Health-Related Quality of Life After Exagamglogene Autotemcel in Patients with Transfusion-Dependent Beta-Thalassemia and Severe Sickle Cell Disease

Amanda M. Li ^1^, Akshay Sharma ^2^, Franco Locatelli ^3^, Markus Marpara ^4^, Josu de la Fuente ^5^, Peter Lang ^6^, Selim Corbacioglu ^7^, Donna Wall ^8^, Puja Kohli ^9^, Siyu Zhang ^9^, Lanju Zhang ^9^, Suzan Imren ^9^, Nanxin Li ^9^, Tina Liu ^9^, Jaime Rubin ^9^, Daoyuan Shi ^9^, Bill Hobbs ^9^, Stephan Grupp ^10^ and Haydar Frangoul ^11^

^1^  British Columbia Children’s Hospital, University of British Columbia, Vancouver, BC, Canada
^2^  Bone Marrow Transplantation and Cellular Therapy, St. Jude Children’s Research Hospital, Memphis, TN, United States of America
^3^  Istituto di Ricovero e Cura a Carattere Scientifico, Ospedale Pediatrico Bambino Gesù Rome, Catholic University of the Sacred Heart, Rome, Italy
^4^  Department of Medicine, Division of Hematology/Oncology, Columbia University, New York, NY, United States of America
^5^  Imperial College Healthcare National Health Service Trust, St. Mary’s Hospital, London, UK
^6^  Department of General Paediatrics, University of Tübingen, Tübingen, Germany
^7^  Department of Pediatric Hematology, Oncology, and Stem Cell Transplantation, University of Regensburg, Regensburg, Germany
^8^  The Hospital for Sick Children, Toronto, ON, Canada
^9^  Vertex Pharmaceuticals Incorporated, Boston, MA, United States of America
^10^  Division of Oncology, Children’s Hospital of Philadelphia, Philadelphia, PA, United States of America
^11^  Sarah Cannon Research Institute at The Children’s Hospital at TriStar Centennial, Nashville, TN, United States of America

**Background:** Exagamglogene autotemcel (exa-cel) is a one-time *ex vivo* CRISPR/Cas9 gene-edited cell therapy shown to eliminate the need for red blood cell (RBC) transfusions in patients (pts) with transfusion-dependent β-thalassemia (TDT) and eliminate vaso-occlusive crises (VOCs) in pts with severe sickle cell disease (SCD). We report changes in health-related quality of life (HRQoL) following exa-cel infusion.

**Methods:** CLIMB THAL-111 and CLIMB SCD-121 are ongoing 24-mo, phase III trials of exa-cel in pts age 12–35 years (y) with TDT and SCD, respectively. Changes in patient reported outcome measures; EuroQol 5 Dimensions 5 Levels of severity (EQ-5D-5L, including descriptive system and visual analog scale [VAS]), Functional Assessment of Cancer Therapy Bone Marrow Transplant (FACT-BMT including FACT-General [FACT-G] and bone marrow transplant subscale [BMTS]), Adult Sickle Cell Quality of Life Measurement Information System (ASCQ-Me; SCD), and 11-point pain Numerical Rating Scale (NRS; SCD) for adults, and EuroQol Quality of Life Scale 5 dimensions youth (EQ-5D-Y) and Pediatric Quality of Life Inventory (PedsQL) for adolescents, were assessed as secondary endpoints.

**Results:** As of 16 April 2023, 29 adults (≥18–≤35 y) and 13 adolescents (≥12–<18 y) with TDT followed for ≥16 months in CLIMB THAL-111 were evaluated. At baseline, mean [SD] EQ-5D-5L health utility US index score (0.87 [0.17]; *n* = 29) was near the general population norm and in line with baseline scores reported for adults with TDT. By month 24, improvements were seen in: EQ-5D-5L health utility US index and EQ VAS scores (mean [SD] change 0.06 [0.28] and 10.7 [18.6]; minimal clinically important difference (MCID) 0.078 and 7 to 10, respectively; *n* = 19), FACT-G total score (8.3 [16.9]; MCID 3 to 7; *n* = 19) and BMTS score (5.6 [5.6]; MCID 2 to 3; *n* = 19). For adolescents, EQ VAS improved through month 12 (7.9 [18.7]; *n* = 13); PedsQL score improved through month 18 (11.5 [12.4]; MCID 4.36; *n* = 10).

As of 14 June 2023, 24 adults (18–35 y) with SCD followed for ≥16 months in CLIMB SCD-121 were evaluated. At baseline, mean (SD) EQ-5D-5L health utility US index (0.78 [0.23]; *n* = 23) and EQ VAS (68.8 [22.7]; *n* = 24) scores were lower than the US general population norm and similar to baseline scores reported for adults with SCD with recurrent VOCs. By month 24, improvements were seen in EQ-5D-5L health utility US index score (mean [SD] change 0.13 [0.19]; MCID 0.078; *n* = 17), EQ VAS score (26.9 [22.6]; MCID 7 to 10; *n* = 17), FACT-G total score (21.0 [18.1]; MCID 3 to 7; *n* = 17), BMTS score (3.9 [5.3]; MCID 2 to 3; *n* = 17) and most ASCQ-Me subscales, including emotional (10.3 [10.9]), social (16.4 [11.0]), and stiffness impacts (6.6 [10.5]; MCID 5 for all). For ASCQ-Me pain-related subscales, the largest improvement was in pain episode frequency (−21.0 [7.7]; MCID −5; *n* = 17) and pain NRS also improved (−1.7 [2.5]; MCID −1; *n* = 17).

**Conclusions:** Participants infused with exa-cel reported sustained and clinically meaningful improvements in HRQoL, demonstrating the broad clinical benefits of exa-cel in pts with TDT and SCD.

## Abstract 42 (Poster): Outcomes of Allogeneic Hematopoietic Cell Transplant (HCT) in Elderly Patients 70 Years and Older with Comparison of Two Cohorts: A Canadian Experience

Yomna Eissa, Eshrak Al-Shaibani, Carol Chen, Shiyi Chen, Igor Novitzky-Basso, Arjun D. Law, Wilson Lam, Fotios Michelis, Auro Viswabandya, Armin Gerbitz, Ivan Pasic, Jeffrey H. Lipton, Dennis D. H. Kim, Jonas Mattsson and Rajat Kumar

University Health Network and Princess Margaret Cancer Centre (PMCC), Toronto, ON, Canada

**Background:** Allogeneic hematopoietic cell transplant (alloHCT) in elderly patients is a challenge, mainly because of the higher incidence of co-morbidities and pre-frail status. In our recent study on alloHCT in patients ≥60 years (y), by Al-Shaibani E, et al. [1], patients who were ≥70 y had worse outcome and high 2-y non-relapse mortality (NRM) of 53%. We therefore made changes in the recent years to try and improve the outcomes of alloHCT in elderly patients. In this study, we will focus on analyzing alloHCT outcomes in patients ≥70 y.

**Methods:** A retrospective analysis of all patients ≥70 y who had an alloHCT from Jan 2015–Dec 2022 at PMCC (*n* = 84) was performed. We then compared those who had an alloHCT from 2015–2019 (Cohort A, *n* = 44) with those who had a transplant from 2020–2022 (Cohort B, *n* = 40).

The median age was 71 y (70–76). The underlying diagnosis was mainly a myeloid malignancy (*n* = 82). Stages of disease at transplant were complete remission (CR) 1 (*n* = 51), CR2 (*n* = 4) or stable disease (*n* = 15, non-AML pts). All patients received reduced-intensity conditioning (RIC) regimens for conditioning including Flu4/Bu2/TBI200 (*n* = 73), Flu/Treo30 (*n* = 7), and Flu/Treo42 (*n* = 3). The graft-versus-host disease (GVHD) prophylaxis regimens used included ATG2/PTCy/CSA (*n* = 32), ATG4.5/PTCy/CSA (*n* = 35), ATG4.5/CSA/MTX (*n* = 7), and ATG2/CSA/MTX (*n* = 3). Different types of donors were used.

The overall survival (OS), event free survival (EFS), NRM, cumulative incidence of relapse (CIR), incidence of acute and chronic GVHD and the length of transplant hospitalization were assessed. The Kaplan Meier curves were plotted for OS and EFS for the overall sample, as well as stratified by the two eras (cohort A and B) and the differences assessed using log-rank tests.

**Results:** Patient and transplant variable were summarized in Table 1. For the whole cohort, the 1-year OS was 53%, the 1-year EFS was 45%, the 1-year NRM was 35%, the 1-year CIR was 18%, and the median number of transplant hospitalization days was 33.5 days (16–116). The incidence of grade I–II acute GVHD was 32%, and grade III–IV acute GVHD was 9.5%; mild to moderate chronic GVHD was 14% and severe chronic GVHD was 2%. When comparing the two cohorts A and B, the 1-year OS was 45% vs. 62% (*p* = 0.10); the 1-year EFS was 37% vs. 58% (*p* = 0.07) and the 1-year NRM was 45% vs. 23% (*p* = 0.04), respectively. The incidence of GVHD and CIR were similar in both cohorts, despite reducing the ATG dose in cohort B. Cumulative incidence of NRM is shown in Figure 1. 

**Conclusions:** There is a statistically significant improvement in NRM and a trend towards improvement in OS and EFS in the patients transplanted during 2020–2022, compared to those transplanted from 2015–2019. This improvement may be due to the changes implemented in the recent years, which include (a) frailty testing, (b) capping the CD34 cell dose, (c) treosulfan for selected indications (MDS/CMML), (d) letermovir for CMV prophylaxis, (e) reducing ATG dose for GVHD prophylaxis, and (f) focus on diet, nutrition, and exercise. We conclude that alloHCT should be offered to select medically eligible elderly patients, but there is a special need to minimize the risks that are age-related.

**Reference**

1. Al-Shaibani, E.; Chen, S.; Chen, C.; Pasic, I.; Michelis, F.V.; Lam, W.; Law, A.; Novitzky-Basso, I.; Gerbitz, A.; Kim, D.D.; et al. Impact of age on hospitalization and outcomes post allogeneic hematopoietic cell transplantation outcome, a single center experience. *Ann. Hematol.* **2023**, *102*, 917–926. https://doi.org/10.1007/s00277-023-05135-3.

## Abstract 43 (Poster): Using Baseline Colonization of Antimicrobial Resistant Organisms in Hematopoietic Cell Transplant Patients to Assess Subsequent Post-Transplant Infections

Tamara Leite ^1^, Nicole Janusz ^2,3^, Leanne Mortimer ^4^, Amanda Carrol ^1^, Natasha Kekre ^1,2,3^, Michael Kennah ^2,3^, Nadia Sant ^1,2,3,4^, Austin Yan ^2,3^, Jaxon Senechal ^3^, *Derek MacFadden ^1,2,3,4^, and *C. Arianne Buchan ^1,2,3^

*Co-PIs

^1^  The Ottawa Hospital Research Institute, Ottawa, ON, Canada
^2^  The Ottawa Hospital, Ottawa, ON, Canada
^3^  Department of Medicine, University of Ottawa, Ottawa, ON, Canada
^4^  The Eastern Ontario Laboratory Association, Ottawa, ON, Canada

**Background:** Infection is one of the leading causes of death post hematopoietic cell transplant (HCT), with most pathogens originating from the patient’s own flora. Knowledge of a patient’s colonizing organisms could be used to guide antibiotic prophylaxis and treatment.

**Purpose:** To establish feasibility of identifying baseline colonization of *Clostridioides difficile* (*C. difficile*) and antimicrobial resistant organisms (AROs), including methicillin-resistant *S. aureus* (MRSA), vancomycin-resistant enterococci (VRE), extended-spectrum beta-lactamase producing Enterobacterales (ESBL), and fluoroquinolone-resistant Enterobacterales (FQRE), to assess the association between pre-transplant colonization and subsequent infection post-transplant.

**Methods:** This prospective cohort study followed 60 HCT patients (34 autologous and 26 allogenic) for three months. Stool samples were collected pre-transplant, to establish participants’ baseline colonization, and twice weekly for up to 21 days post-transplant. Clinical data including infectious complications, neutropenia, disease relapse, and mortality was collected via chart review. Positive culture results along with clinical history were used to identify infectious episodes, categorized by pathogen/ARO type.

**Results:** Patient infection incidence was captured in Table 1. Baseline colonization with *C. difficile* and/or AROs was identified in 27% (16/60) of patients, with one patient testing positive for both *C. difficile* and an ARO. 10% (6/60) of the study cohort were colonized with *C. difficile*. 18% (11/60) of patients were colonized with an ARO, including VRE (4/11), ESBL and FQRE (4/11), and FQRE alone (3/11). In addition, 16 patients were found to be colonized with other Gram-negative organisms, the majority of which harboured inducible beta-lactamases (AMP-C). Of the 8 patients colonized with ESBL or FQRE, one (12.5%) developed a bloodstream infection (BSI) with ESBL *K. pneumonaie* bacteremia. The median length of neutropenia was 11 days (Interquartile range 7–32), with 72% (43/60) of patients developing febrile neutropenia.

47% (28/60) of patients experienced at least one culture-positive infection within the first three months post-HCT (Table 1). BSIs accounted for 29% (15/52) of all infections. A total of 4 patients were diagnosed with resistant Gram-negative bacteremia, of which 3 were ESBL *K. pneumonaie. C. difficile* infections (CDI) occurred in 17% of the study cohort (10/60), with two individuals having recurrent CDIs, and is discussed separately.

**Conclusions:** This comprehensive analysis of infectious episodes post-HCT highlights the significant burden of infections in this subset of highly immunocompromised patients. Our study demonstrates feasibility of establishing pre-transplant baseline colonization and identification of resistant organisms that may inform downstream infection risk. Larger future studies are needed to evaluate use of colonization data for prediction of post-transplant infection and the role of targeted prophylaxis or treatment based on patient’s flora.

## Abstract 44 (Oral): COVID-19 Vaccine Immunogenicity in Patients Who Have Undergone Hematopoietic Cell Transplant: A Prospective Real World Observational Multi-Site Canadian Study (Award Recipient—Clinical Trials/Observations)

Sita Bhella ^1^, Abi Vijenthira ^1^, Michael Sebag ^2^, Peng Wang ^3^, Allison M. Wilkin ^4^, Katrina Huenkin ^1^, Curtis Cooper ^4,5^, Marc Andre Langlois ^4,6^, and C. Arianne Buchan ^4,5^

^1^  Division of Medical Oncology and Hematology, Princess Margaret Cancer Centre, University of Toronto, Toronto, ON, Canada
^2^  Division of Hematology, McGill University Health Centre, Montreal, QC, Canada
^3^  Division of Hematology, University of Alberta, Edmonton, AB, Canada
^4^  Ottawa Hospital Research Institute, Ottawa, ON, Canada
^5^  Division of Infectious Diseases, The Ottawa Hospital, Ottawa, ON, Canada
^6^  Department of Medicine, University of Ottawa, Ottawa, ON, Canada

**Background:** Immune response to vaccines, including COVID-19 vaccines, is diminished in immunocompromised populations and thus, an emphasis has been placed on ensuring these patients are adequately immunized and receive booster doses of vaccines. As SARS-CoV-2 persists, there is a need to design optimal recommendations for COVID-19 primary immunization and booster-dose schedules for patients who have undergone hematopoietic cell transplant (HCT) and cellular therapies.

**Purpose:** To quantify the humoral immune response engendered by SARS-CoV-2 vaccination in patients with hematologic malignancies who have undergone HCT or cellular therapy.

**Methods:** This prospective study enrolled 944 patients with hematologic malignancies between August 2021 and January 2023 from 11 sites in Canada. 789 pts were eligible for analysis including 111 who underwent autologous stem cell transplant (ASCT), 87 who underwent allogeneic stem cell transplant (alloHCT) and 21 who were treated with cellular therapy. Participants were followed longitudinally with study questionnaires on clinical outcomes and adverse events following immunizations, and blood samples (finger-prick dried blood spot cards) were collected for detection of antibodies surrounding vaccine doses. Samples were processed via high throughput ELISA assay to detect serum antibodies against nucleocapsid (N) and spike (S) proteins.

**Results:** We determined humoral immune response prior to, and within 7 to 42 days, of last dose of SARS-CoV-2 vaccination in patients with history of ASCT and alloHCT for treatment of hematologic malignancy (*n* = 198). At time of study entry, 56% of alloHCT pts and 77% of ASCT had received 2–3 vaccine doses.

Post dose 3 the proportion of the overall cohort (*n* = 280), alloHCT (*n* = 26) and ASCT (*n* = 47) who demonstrated a positive anti-N antibody was 6.1%, 3.8%, and 6.4%, respectively. Post dose 3 the proportion of the overall cohort (*n* = 280), alloHCT (*n* = 26) and ASCT (*n* = 47) who demonstrated a positive anti-S antibody was 70.4%, 88.5% and 83.0%.

Post dose 5 the proportion of the overall cohort (*n* = 156), alloHCT (*n* = 18) and ASCT (*n* = 28) who demonstrated positive anti-N was 12.8%, 22.2% and 14.3%. Post dose 5, the proportion of the overall cohort (*n* = 156), alloHCT (*n* = 18) and ASCT (*n* = 28) who demonstrated positive anti-S was 91.7%, 100% and 100%. The proportion of patients with anti-N and anti-C seropositivity between October 2021 and February 2023 is shown in Figure 1. 

The number who experienced a self-reported COVID-19 infection at any time during the study was 243 (33%) for the entire cohort, 23 (28%) for alloSCT, 25 (24%) for ASCT.

**Conclusions:** This prospective cohort study showed that humoral immune response improved with subsequent doses of COVID-19 vaccines. Booster doses play a role in increasing antibody response after a primary series in patients with hematologic malignancy treated with transplant or cellular therapy. Further analyses are pending to explore predictors of response, humoral immunity over time since treatment, correlates of antibody response with neutralizing antibody levels, and comparisons to cellular immunity.

## Abstract 45 (Poster): Different Metabolomic Profiles in Adult Compared to Pediatric Chronic Graft-Versus-Host Disease (Award Recipient—Clinical Trials/Observations)

Fabiola Wu Wu ^1,3^, Tashi Rastogi ^1^, Bernard Ng ^3^, Liam Johnston ^2^, Sayeh Abdossamadi ^1^, Amina Karimina ^1^, Madeline Lauener ^1^, Elena Ostroumov ^1^, Barnaby Malong ^1^, Dong Jun Zheng ^1^ and Kirk R. Schultz ^1^

^1^  Michael Cuccione Childhood Cancer Research Program, British Columbia Children’s Hospital Research Institute, Vancouver, BC, Canada
^2^  Department of Statistics, Centre for Molecular Medicine and Therapeutics, British Columbia Children’s Hospital, University of British Columbia, Vancouver, BC, Canada
^3^  Department of Pathology and Laboratory Medicine, University of British Columbia, Vancouver, BC, Canada

**Background:** Chronic graft-versus-host disease (cGvHD) is the leading cause of morbidity following allogenic hematopoietic stem cell transplant (HSCT). Previously [1], we found biological differences associated with cGvHD in a large pediatric study, ABLE1.0 (PBMTC1202) and validated these patterns using archival samples from a Children’s Oncology Group trial, ASCT0031. In two pediatric cGvHD studies, we demonstrated significant elevation of α-ketoglutarate, kynurenine, glutamic acid and decreased C8.

**Purpose:** To compare metabolomic cGvHD profiles in a separate adult cohort compared to previous metabolomic changes seen in children.

**Methods:** One hundred twenty-one patients were enrolled in an adult cGvHD biomarker study. This study included 20 patients with cGvHD onset between 100–365 days compared to 101 patients with no cGvHD, with samples obtained at 3, 6, and 12 months post-HSCT. Plasma was separated from whole blood and examined using direct injection mass spectrometry with reverse-phase LC-MS/MS for ~142 metabolites. Differences in metabolite levels between cGvHD and non-cGvHD patients were compared using multiple regression and considered significant if a metabolite met all 3 of the following criteria: (1) *p*-value < 0.05; (2) effect ratio of ≥1.3 or ≤0.75; and (3) receiver operating characteristic (ROC) area under the curve (AUC) ≥ 0.60.

**Results:** In a mixed analysis including all cGvHD onset compared to those that did not develop cGvHD, we found a significant decrease of trimethylamine N-oxide and hippuric acid at cGvHD onset (Table 1). Looking at the time dependence of metabolite changes, we found that trimethylamine N-oxide was significant early (3 and 12 months) while hippuric acid was only significant at 3 months. Interestingly, indole acetic acid associated with a late onset cGvHD (8–12 months matched to 12-month controls).

**Conclusions:** We were unable to see significant increases of metabolites associated with the onset of pediatric cGvHD. The adult cohort instead was characterized by alteration in intestinal microbiome-associated metabolites, including trimethylamine N-oxide and hippuric acid, in early-onset cGvHD and indole acetic acid in late-onset cGvHD. This suggests there may be different age-related metabolite changes seen in adult versus pediatric cGvHD and requires validation of separate cohorts.

**Reference**

1. Subburaj, D.; Ng, B.; Kariminia, A.; Abdossamadi, S.; Lauener, M.; Nemecek, E.R.; Rozmus, J.; Kharbanda, S.; Kitko, C.L.; Lewis, V.A.; et al. Metabolomic identification of α-ketoglutaric acid elevation in pediatric chronic graft-versus-host disease. *Blood* **2022**, *139*, 287–299. https://doi.org/10.1182/blood.2021013244.

## Abstract 46 (Poster): Stability Program Analysis for Cryopreserved Hematopoietic Progenitor Cell

Kelly Murphy ^1^, Denise Swaby ^1^, Nicholas Dibdin ^1^, Kathy Ganz ^1^, Matthew D. Seftel ^1,2^, and Jelena L. Holovati ^1,3^

^1^  Stem Cell Service, Canadian Blood Services, Ottawa, ON, Canada
^2^  Department of Medicine, University of British Columbia, Vancouver, BC, Canada
^3^  Department of Laboratory Medicine and Pathology, University of Alberta, Edmonton, AB, Canada

**Background:** A stability program is a requirement by FACT-JACIE for cryopreserved products to monitor hematopoietic progenitor cell (HPC) quality indicators, such as viability and potency. A pre-processing cell dose is commonly used when manufacturing includes cryopreservation, even though loss of some HPCs during freezing and thawing is assumed. Quality metric evidence may include clinical outcome metrics and some form of post-thaw testing of stored samples. In the autologous transplant setting, Canadian Blood Services (CBS) Edmonton has previously correlated timely engraftment to a minimum post-thaw CD34^+^ dose of ≥2 × 10^6^/kg or ≥60% recovery of pre-freeze CD34^+^ dose.

**Purpose:** Although not prescriptive in terms of evaluation approaches, HPC testing strategy, or sample size, the FACT-JACIE expectation is that a HPC stability testing program contributes to the evidence that the HPC manufacturing validated state is maintained throughout HPC processing and storage. The aim of this study is to present a retrospective data analysis approach for the autologous HPC long-term stability program at the CBS Edmonton stem cell lab.

**Methods:** Retrospective data was compiled to represent HPC cryopreservation each year from 2015 to 2020. Metrics included days to absolute neutrophil count and platelet (ANC/PLT) engraftment as well as post-thaw recoveries of viable CD34^+^ and colony forming unit-granulocyte/macrophage (CFU-GM) cells. Equality of variances between yearly data sets was tested using Levene’s test. A two-sample *t*-test was performed to compare 2021 stability data to our historical baseline data.

**Results:** Levene’s test showed that the population’s variances are statistically insignificant (F = 0.72935), indicating sufficient robustness to the homogeneity of variances assumption to proceed with the two-sample *t*-test analysis, which showed that while the 2021 group (*n* = 15) was not statistically different from the 2015–2020 baseline group (*n* = 115) when comparing % recovery post-thaw (CD34^+^/CFU-GM) or days to ANC engraftment (*p*-values 0.608092, 0.750164 and 0.437775, respectively), there was a statistically significant delay in days to platelet engraftment (23.00 ± 10.87 versus 20.42 ± 5.50, respectively, *p* < 0.05). Further investigation attributed this statistical difference to an outlier delayed platelet engraftment case of 51 days, in a lymphoma patient transplanted with CD34 viable cell dose of 5.30 × 10^6^/kg, with acceptable post-thaw testing and ANC engraftment.

**Conclusions:** Our lab has approached meeting FACT-JACIE requirements for a stability program that annually evaluates the quality of cryopreserved cellular therapy products by applying statistical analysis of post-thaw testing and time to engraftment as process monitoring metrics, demonstrating no trends in overall post-thaw recoveries and time to engraftment data that can be attributed to the manufacturing attributes of the stem cell product.

## Abstract 47 (Poster): Nucleated Cell Content and Plasma Reduction Practices in Cryopreserved Autologous Hematopoietic Progenitor Cell-Apheresis (HPC-A) Products

Kelly Murphy ^1^, Lisa Martin ^1^, Denise Swaby ^1^, Nicholas Dibdin ^1^, Kathy Ganz ^1^, Matthew D. Seftel ^1,2^, David S. Allan ^1,3^ and Jelena L. Holovati ^1,4^

^1^  Stem Cell Service, Canadian Blood Services, Ottawa, ON, Canada
^2^  Department of Medicine, University of British Columbia, Vancouver, BC, Canada
^3^  Department of Medicine, University of Ottawa, Ottawa, ON, Canada
^4^  Department of Laboratory Medicine and Pathology, University of Alberta, Edmonton, AB, Canada

**Background:** Canadian Blood Services (CBS) cryopreserves autologous hematopoietic progenitor cell-apheresis (HPC-A) products for four transplant programs in two cell processing facilities based in Edmonton, Alberta and Ottawa, Ontario. As part of an overall quality improvement initiative, CBS intends to standardize HPC cryopreservation at these two sites, including DMSO concentration and degree of plasma reduction. Some studies have suggested that high total nucleated cell (TNC) content in a cryopreserved HPC-A graft predicts adverse infusion reactions or delayed engraftment after autologous transplantation. While the Edmonton facility has a maximum TNC that can limit the degree of plasma reduction, the Ottawa facility has no such defined criteria.

**Purpose:** The aim of the study is to investigate the differences in HPC cryopreservation between the two CBS manufacturing programs, with a focus on the impact of high TNC in HPC-A products on relevant patient outcomes.

**Methods:** A retrospective cohort analysis of HPC product nucleated counts (white blood cell content per bag) for HPC-A collections that were infused at both CBS manufacturing sites between 1 April 2022 and 31 March 2023 was performed. Outcomes of interest were time to platelet (PLT) and absolute neutrophil count (ANC) engraftment as well as HPC-A infusion adverse reactions.

**Results:** A total of 761 HPC autologous units were distributed between 1 April 2022 and 31 March 2023 (Edmonton 434, Ottawa 327). Despite the manufacturing differences between sites, mean ANC and PLT time to engraftment uniformly met the expected local transplant centre timeline criteria. In Ottawa, between April and September 2023, 14 of 127 (11%) products exceeded Edmonton’s maximum TNC limit of 800 × 10^9^/L (947.2 ± 106.0 × 10^9^/L; range 802.1–1197.3 × 10^9^/L). These 14 units were volume reduced to two cryogenic bags and infused with no adverse reactions and acceptable engraftment results (ANC < 17 days, mean 12.5 ± 1.2 days; PLT < 35 days, mean 18.6 ± 3.3 days). In contrast, during the same time period, there was only one instance of high TNC HPC-A product in Edmonton (876 × 10^9^/L; volume reduced to 4 bags), with patient experiencing rigors and fever upon product infusion and delayed engraftment (ANC = 21 days, PLT > 30 days, compared to overall days to engraftment of 12.8 ± 1.9 and 24 ± 10.1).

**Conclusions:** We found that HPC manufacturing standardization should include consideration of TNC and granulocytes as a quality control acceptance criterion for HPC-A products. Introducing an upper limit for TNC as currently used in Edmonton could have a noticeable impact on transplantation practice in Ottawa. The risk of excess nucleated cells as compared to the risk of infusing excess volume by withholding plasma reduction needs to be further assessed.

## Abstract 48 (Poster): Choosing Wisely: Reducing Inappropriate Blood Work in Patients Undergoing Autologous Stem Cell Transplant on an Inpatient Transplant Unit (Award Recipient—Laboratory/Quality)

Madelaine Bohnert ^1,2^, Katlynn Schellenberger ^1,2^, Christine Luu ^1,3^, Victor Pope ^1,3^, Ashley E. Smith ^2^, Sarah Sayles ^2^, Alan Gob ^1,2,3,4^, and Uday Deotare ^1,2,3,4^

^1^  London Regional Cancer Program, London, ON, Canada
^2^  London Health Sciences Centre, London, ON, Canada
^3^  Department of Medicine, Division of Haematology, Western University, London, ON, Canada
^4^  Centre for Quality, Innovation and Safety, Western University, London, ON, Canada

**Background:** Inappropriate laboratory testing has significant negative impacts on patients and healthcare resources. When large blood volumes are drawn daily, a decline in hemoglobin of approximately 10 g/L had been observed. While laboratory testing is an essential component of providing comprehensive care in transplant patients, an estimated 28% of laboratory testing is ordered inappropriately. On our adult inpatient transplant unit, blood work is electronically ordered upon admission and is rarely reviewed for ongoing appropriateness due to competing tasks. We identified a possibility for decreasing blood draws in our autologous stem cell transplant (ASCT) patient population after developing and successfully implementing a bloodless transplant protocol for four Jehovah’s Witness patients undergoing ASCT.

**Purpose:** Our quality improvement project aimed to identify the root causes and to reduce inappropriate blood work in our inpatient ASCT population at London Health Sciences Centre (LHSC) by 30% by 1 May 2024.

**Methods:** We primarily followed the Model for Improvement QI Methodology and a modified version of the Model for Understanding Success in Quality Improvement. We identified patients who underwent ASCT from the LHSC database to collect baseline data on blood draw volumes and cost of tests during admissions. We determined average blood volume collected for patients, iatrogenic blood draw and cost. Average nursing time was determined via direct observation of blood collections. Additional electrolyte infusions and blood transfusions were also calculated. Finally, we completed a root cause analysis using an Ishikawa Fishbone Diagram (Figure 1).

**Results:** To establish baseline data, we identified 23 patients who underwent ASCT at LHSC in 2022. An average of 210 mL (range: 131–428) (Figure 2) of blood was drawn for daily blood work on an average inpatient stay of 19.4 days (range: 12–32). Additional tests were conducted on an average 18 times (range: 0–63) with an increased iatrogenic patient blood loss of 180 mL. Each blood draw required 6 min of nursing time to complete, not including time to interpret and act upon findings. Next, we developed change ideas using our baseline data. These were to decrease parameters for our inpatient ASCT electrolyte replacement protocol, decrease parameters for reassessing electrolytes after electrolyte replacement, by decreasing blood draws from daily to 3 times per week, and to eliminate blood draws from day +1 to day +5. We plan to pilot changes and refine interventions using Plan-Do-Study-Act (PDSA) cycles. This project is ongoing and will be updated at the time of the CTTC 2024 conference.

**Conclusions:** Many factors contribute to inappropriate blood draws, however, reducing unnecessary bloodwork in ASCT patients to decrease iatrogenic blood loss is possible using QI methodology. We will implement and refine our change ideas through PDSA cycles to reduce inappropriate blood work in inpatient ASCT patients.

## Abstract 49 (Oral): Graft Manipulation and Cryopreservation Effect on Different Graft Components and Its Potential Clinical Applications (Award Recipient—Laboratory/Quality)

Mohammed Kawari ^1,2^, Emily Fu ^1^, Shiyi Chen ^1^, Mileidys Alvarez ^1^, Jessica McLeod ^1^, Muhammad Badawi ^1^, Arpita Parikh ^1^, Rashied Kawshermolla ^1^, Racheljihye Kim ^1^, Bramdeo Motiram ^1^, Lynn Jean ^1^, Miyada Himmat ^1^, Lydia Morrison ^1^, Keanu Herzog ^1^, Madhavi Gerbitz ^3^, Kai Marks ^3^, Megan Nelles ^1^, Eunyoung Cho ^1^, Ahmed Najemeldin ^1^, Igor Novitzky-Basso ^1,2^, and Armin Gerbitz ^1,2,3^

^1^  University of Toronto, Toronto, ON, Canada
^2^  Princess Margaret Cancer Centre, Toronto, ON, Canada
^3^  Philip Orsino Cell Processing Laboratory, Toronto, ON, Canada

**Background and Purpose:** The primary active component within an allogeneic stem cell graft (aSCG) is the CD34^+^/CD38^−^ hematopoietic stem cell. Viability of the stem cells is crucial and impacted by prolonged *ex vivo* time, cell density in the bag, and later on, cryopreservation. However, success of allogeneic stem cell transplantation (aSCT) also relies on the presence of functional T cells to facilitate engraftment and ensure graft versus leukemia effects. Although fresh stem cell grafts are commonly used, the COVID-19 pandemic prompted a substantial shift towards cryopreservation as a risk-mitigation strategy for uncertain supply chains.

**Methods:** Between March 2020 and December 2022, a prospective analysis of the cellular components of all aSCG on arrival at the institution and after cryopreservation was conducted. We also gathered data on *ex vivo* time (time from the end of apheresis to infusion or cryopreservation) and cell concentration in the apheresis bag. Viability analysis was conducted on various cell populations, including hematopoietic progenitors (CD34), monocytes (CD14), T-cells (CD3, CD4, and CD8), B-cells (CD19), and NK-cells (CD56). A total of 425 grafts from matched related, haploidentical, and matched or mismatched unrelated donors were analyzed. 305 grafts were infused fresh, while 120 were cryopreserved.

**Results:** The median *ex vivo* time for freshly infused grafts was 26.7 hours (h) (*n* = 305, range 2.2–64.5), and for cryopreserved grafts was 33 h (*n* = 120, range 1.6–87.6, *p* = 0.43, Table 1 and Table 2). Upon arrival, cell viability was minimally affected by cell concentration or *ex vivo* time. However, cryopreservation had notable negative impact on viability of various cell types independent of cell concentration and *ex vivo* time. While CD34^+^ cells exhibited the highest viability at 87%, CD3^+^ cells were the most sensitive, with 68.2% viability. Unrelated donor cells had the lowest viability, possibly due to longer *ex vivo* time. *Ex vivo* time and cell concentration did not significantly impact CD34 viability (*p* = 0.24 and *p* = 0.21, respectively), however a correlation was found between graft cell concentration, *ex vivo* time, and viability of CD3, CD4, and CD8.

**Conclusions:** The study findings reveal that peripheral blood graft components are influenced by travel time, cell concentration, and cryopreservation, with variable impacts on different cell types. CD34 cells exhibit higher resilience, whereas T cells are notably affected. Extended *ex vivo* time and high cell concentration disproportionately diminish T-cell viability when grafts are cryopreserved. The observed adverse effects on T-cell viability could explain the reported increased incidence of poor graft function and delayed engraftment, as recipients of frozen grafts with long *ex vivo* time receive substantially fewer T cells. Further investigation aims to correlate these findings with clinical outcomes and explore post-transplant immune reconstitution in affected cell types is required.

## Abstract 50 (Poster): Paradigm 3: An Electric Solution to Document Control Non-Compliance

Taylor Penner-Zuk, Edwin Brindle, and Kylie Lepic

Hamilton Health Science, Juravinski Hospital and Cancer Centre, Hamilton, ON, Canada

**Background and Purpose:** The Cellular Therapy and Transplantation (CTT) program at Hamilton Health Science (HHS) Juravinski Hospital and Cancer Centre recognized an urgent need to replace its document management system. Various factors contributed to this including: increased non-conformances and audit citation, program expansion (increased number of cellular therapy procedures and products, geographical footprint and staffing) and rise in controlled documents required. The goal of the project was to select a suitable software vendor (Paradigm 3 (P3)) and evaluate effectiveness of the software compared to the current paper system.

**Methods and Results:** Comparative analysis highlighted several benefits of P3. The enhanced capability of P3 outweighed the capital outlay for the document control functions and P3 modules were designed for additional quality management functions such as deviation management, internal audits, and staff training. P3 met FDA 21CFR part 11 for electronic signature requirements, process flow and robust controls required by FACT (Foundation for the Accreditation of Cellular Therapy) and Health Canada. Two tools were used in the comparative analysis: A time and motion study and a qualitative user survey. Initial time and motion study highlighted significant bottlenecks with workflow and distribution. Manual paper process took approximately 4034 min per document from drafting to release. The elimination of the paper processing saw an overall time savings of 55%. P3 post-implementation saw a 99% increase in time savings during the document approval process alone.

The qualitative user survey revealed mixed results. Of the 22 respondents, 18 successfully completed the survey and accessed documents through Paradigm. Of the 18 respondents that completed the survey, 44% found Paradigm difficult to navigate when compared to the HHS Policy Library (previous electronic document retrieval tool) and 83% had moderate difficulty finding desired documents using the search function. When asked about ease of completing ‘read & sign’ document training, results were evenly distributed among the available choices, with ‘somewhat difficult to navigate’, ‘somewhat easy to navigate’ and ‘extremely easy to navigate’ all yielding 22%.

**Conclusions:** P3 has improved the document control process in regard to timesaving, but there is a staff learning curve. Increased training of P3 is required in order to garner continued staff support and use. Future projects will include the implementation of the improvement and training module to track and trend deviations, automate audit reporting, equipment management and staff yearly training and competency.

## Abstract 51 (Poster): Complex Medical Hematology Nurse Practitioner (NP) Fellowship

Kari Kolm

Juravinski Hospital and Cancer Centre, Hamilton, ON, Canada

Graduate level Nurse Practitioner education is not specific to malignant hematology and most new NPs do not find themselves adequately prepared to work in the highly demanding, fast paced hematology environment. The Complex Malignant Hematology Nurse Practitioner Fellowship at Juravinski Hospital and Cancer Centre is the first in kind Canadian fellowship to prepare Nurse Practitioners to competently and confidently work in malignant hematology. Recruitment and retention of nurse practitioners can be partly linked to being adequately trained and able to fulfill the responsibilities and domains of their role. This fellowship aims to do this through mandatory and elective clinical rotations, education, leadership opportunities as well as leading a research or continuous quality improvement (CQI) project. Research is supported through a linkage with the Faculty of Nursing at McMaster University. This presentation will discuss how this innovative fellowship was created, the basic tenants of the fellowship and the evaluative components of this new fellowship.

## Abstract 52 (Oral): Obesity Dosing of High Dose Melphalan in Patients with Multiple Myeloma Undergoing Autologous Hematopoietic Cell Transplant (Award Recipient—Pharmacy, Nursing, and Other Transplant Support)

Morgan Hopkins ^1^, Deanna Caldwell ^1^, Kate Kelly ^1^, Adrienne Fulford ^1,2^, Anargyros Xenocostas ^1,2,3^, and Uday Deotare ^1,2,3^

^1^  London Health Sciences Centre, London, ON, Canada
^2^  Blood and Marrow Transplant Program, London Health Sciences Centre, London, ON, Canada
^3^  Division of Hematology, Schulich School of Medicine and Dentistry, Western University, London, ON, Canada

**Background:** The standard of care for transplant-eligible multiple myeloma (MM) involves high-dose melphalan chemotherapy followed by autologous hematopoietic stem cell transplant (ASCT). In 2014, the American Society for Blood and Marrow Transplantation recommended using actual body weight-based melphalan dosing regardless of patient weight. The recommendation was based on variable program practices; however there remains a paucity of evidence for melphalan dosing in obese ASCT patients to date.

**Purpose:** London Health Sciences Centre has historically dosed melphalan on adjusted body weight for patients greater than 120% of their ideal body weight. This study aimed to determine if 40% adjusted body weight-based melphalan dosing impacts efficacy and safety outcomes in our obese, MM patients undergoing ASCT. The primary objective assessed the difference in progression-free survival (PFS) at 2 years between obese and non-obese patient groups. Time to engraftment and safety outcomes including length of stay, infection rates, and transfusion requirements were secondary objectives.

**Methods:** A retrospective chart review was completed for MM patients undergoing ASCT from 1 January 2018 to 31 May 2021. Obese and non-obese patients who received melphalan chemotherapy prior to autologous transplant were included. Obese patients were defined as being greater than 120% above ideal body weight and having received 40% adjusted body-weight-based melphalan dosing. Data regarding transplant, including safety outcomes, and relapse within 2 years post-ASCT were collected.

**Results:** We included 73 patients in this study, 52 obese and 21 non-obese, with similar baseline characteristics except for actual body weight (*p* < 0.001). At 2 years post ASCT, 10 (19%) obese patients and 5 (24%) non-obese patients experienced MM progression (*p* = 0.59) (Figure 1). Time to neutrophil engraftment [14.5 and 13.3 days, (*p* = 0.37)] and platelet engraftment [13.4 and 13.5 days (*p* = 0.71)] were also similar between obese and non-obese groups. No statistically significant differences in safety were observed including: length of stay (*p* = 0.08), infection rates (*p* = 0.33), rates of upper and lower gastro-intestinal mucositis (*p* = 0.5) and transfusion requirements [packed red blood cells (*p* = 0.74) and platelets (*p* > 0.99)] (Figure 2).

**Conclusions:** The use of 40% adjusted body-weight-based melphalan dosing in obese patients resulted in similar PFS at two years when compared to non-obese, actual body-weight-based patients. This retrospective study is the first published data set using a specific weight adjustment (40%) showing similar outcomes using adjusted body weight-based melphalan dosing in obese ASCT patients.

## Abstract 53 (Poster): Implementation of a Social Work-Led Advance Care Planning Clinic for CAR T-Cell Therapy Patients (Award Recipient—Pharmacy, Nursing, and Other Transplant Support)

Breffni Louise Hannon ^1^, Warren Lewin ^2,3^, Janet Papadakos ^1^, Tina Papadakos ^1^, Eden Klein ^1^, Claudia Abreu- Costa ^4^, Christine Chen ^3,5^, and Sita D. Bhella ^3,4^

^1^  Princess Margaret Cancer Centre, Toronto, ON, Canada
^2^  University Health Network, Toronto, ON, Canada,
^3^  Department of Medicine, University of Toronto, Toronto, ON, Canada
^4^  Medical Oncology and Hematology, Princess Margaret Cancer Centre, Toronto, ON, Canada
^5^  Princess Margaret Hospital, Toronto, ON, Canada

**Background:** Chimeric antigen receptor (CAR) T-cell therapies offer encouraging results for a subset of patients with advanced hematological malignancies; 5-year progression free survival for patients who undergo CAR T-cell therapy as third line therapy is approximately 31% [1]. Despite this, advance care planning (ACP) discussions with patients prior to treatment are not routinely offered. ACP discussions allow patients an opportunity to reflect on their wishes and values, and to let them share what matters to them, as well as let their healthcare team know what kind of health and personal care they wish to receive now and in the future as their health changes.

**Purpose:** To demonstrate the feasibility of implementing a social work-led ACP clinic for patients embarking on CAR T-cell therapy at the Princess Margaret Cancer Centre (PM), in Toronto, Canada.

**Methods:** An interdisciplinary team comprising a social worker, hematologists, palliative care physicians, a hematology nurse coordinator, and cancer care education specialists reviewed the ACP literature and informed the content of a dedicated ACP clinic.

Patients enrolled in the CAR T-cell therapy program at PM were invited to meet one-on-one with a hematology social worker. The meeting comprised a guided interview where ACP was explained and explored across key domains of illness understanding: exploring goals and values, and planning for the future. The consultation was documented in the patients’ electronic medical record.

**Results:** Of the 37 patients approached since June 2023, 34 patients completed an ACP consultation. All participating patients identified their preferred substitute decision-maker (SDM). Three of 34 (9%) were interested in a palliative care consultation. Code status documentation at time of admission was: full (23, 68%), no order (11, 32%) and unknown/not applicable (3, 9%). Ten consecutive patients evaluated for CAR T-cell therapy from 2023 prior to the launch of the ACP clinic were reviewed, which demonstrated that all had a SDM identified, and code status documentation was: full (3, 30%) and no order (7, 70%). None had palliative care involved in their care at time of CAR T-cell admission. Key learnings from the clinic to date include the importance of a dedicated team member with whom patients can reflect on prognostic uncertainty as well as discuss their goals and values, providing practical support around legal and financial planning for the future, and opportunities for healthcare navigation including introducing the concept of integrated palliative care alongside, rather than instead of, CAR T-cell therapy.

**Conclusions:** A structured social work-led ACP clinic for patients embarking on CAR T-cell therapy is feasible and provides valuable support. Future studies should explore the impact of the clinic on patient outcomes and aggressiveness of care at the end-of-life; as well as different models for integrating ACP discussions into routine care for patients with advanced hematological malignancies. Expansion of the clinic for other advanced malignancies can be considered.

**Reference**

1. Chong, E.A.; Ruella, M.; Schuster, S.J. Five-Year Outcomes for Refractory B-Cell Lymphomas with CAR T-Cell Therapy. *N. Engl. J. Med*. **2021**, *384*, 673–674. https://doi.org/10.1056/nejmc2030164.

## Figures and Tables

**Figure 1:**
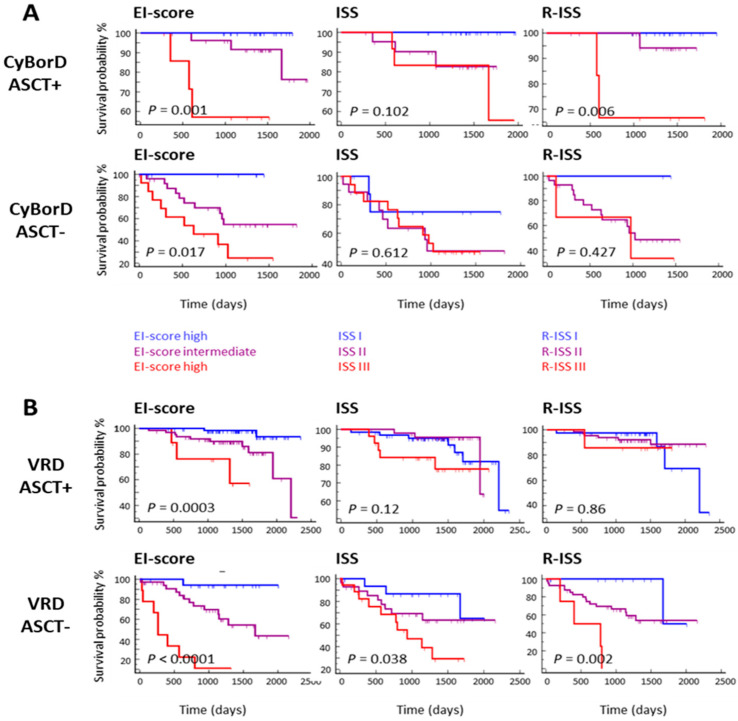
Graphical presentations of Kaplan Meier analysis considering only Multiple Myeloma Research Foundation CoMMpass patients who (**A**). received CyBorD as induction regimen with or without ASCT or (**B**). received VRD as induction regimen with or without ASCT.

**Figure 1:**
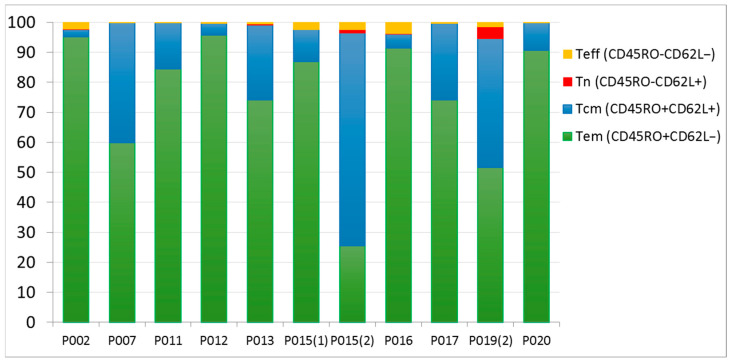
CD8^+^ T cell memory profiles.

**Figure 2:**
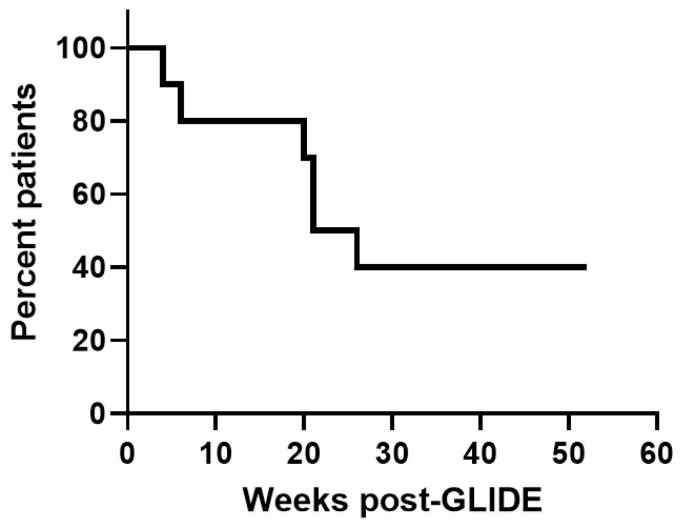
Overall survival.

**Figure 1:**
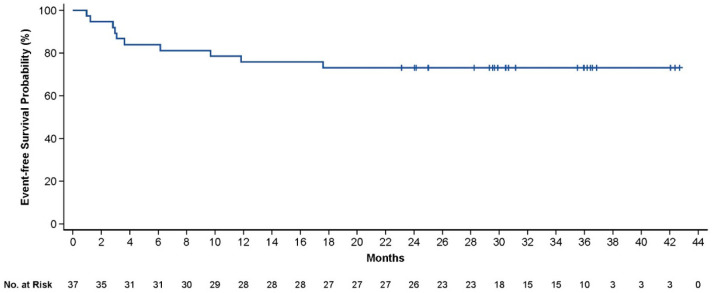
Event-free survival (EFS). The 36-month EFS rate (95% CI) was 73.0% (55.6–84.4).

**Figure 2:**
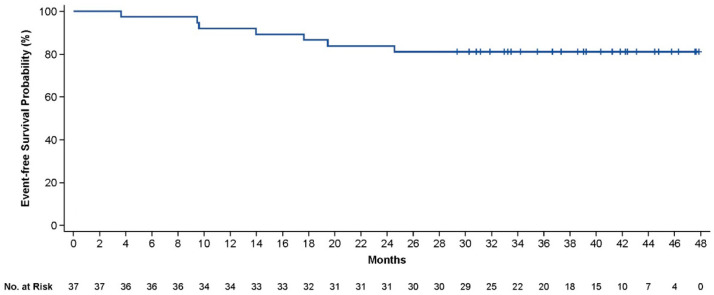
Overall survival (OS). The 36-month OS rate (95% CI) was 81.1% (64.4–90.5).

**Figure 1:**
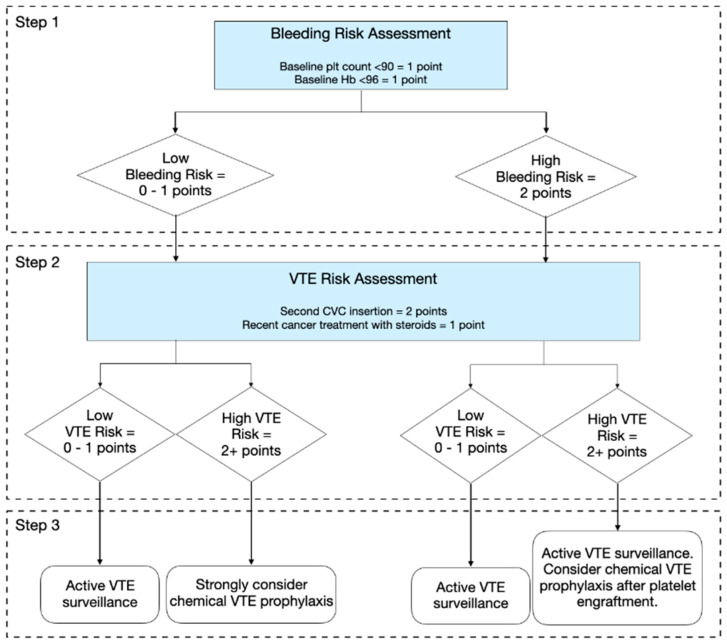
Venous thromboembolism and bleeding in marrow transplant (VBMT) risk assessment model (RAM) flow diagram. The RAM combines bleeding and VTE risk assessment models to be used in the immediate 90-day post-transplant period. Step 1 includes risk stratification of patients based on bleeding risk. Step 2 includes risk stratification of patients based on VTE risk. Step 3 includes the clinic action to take.

**Figure 1:**
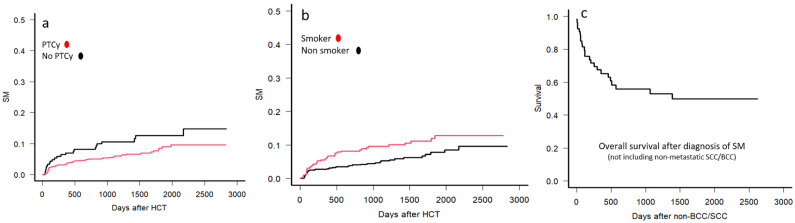
(**a**) Cumulative incidence of secondary malignancies (SM) for patients receiving versus not receiving post-transplant cyclophosphamide. (**b**) Cumulative incidence of SM for smokers versus non-smokers. (**c**) Overall survival of patients after diagnosis of SM.

**Figure 1:**
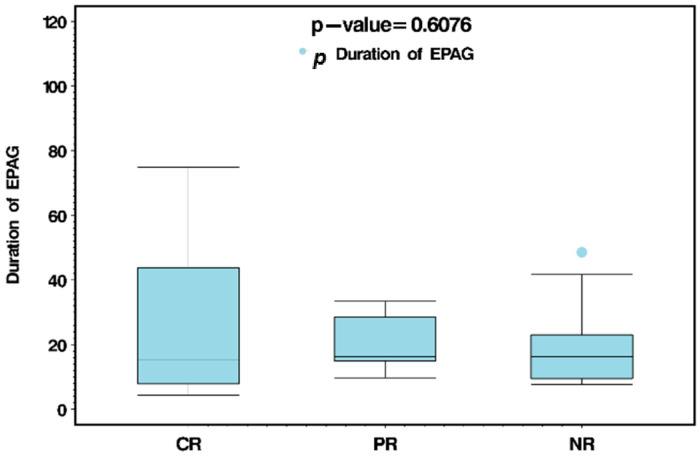
Boxplot of duration of EPAG in weeks, by EPAG response.

**Figure 1:**
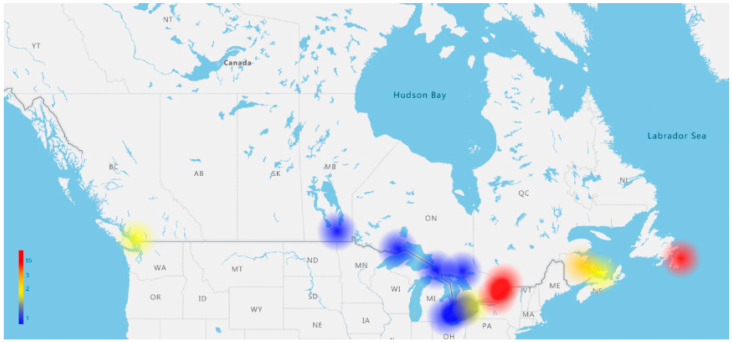
Heat map of Canada-wide centres referring patients to The Ottawa Hospital for CAR-T therapy. Red shades indicate regions with a higher number of centres referring patients for CAR-T therapy, while blue shades represent areas with fewer referral centres. Yellow and orange shades denote regions falling in-between, indicating varying degrees of centre density in those areas.

**Figure 1:**
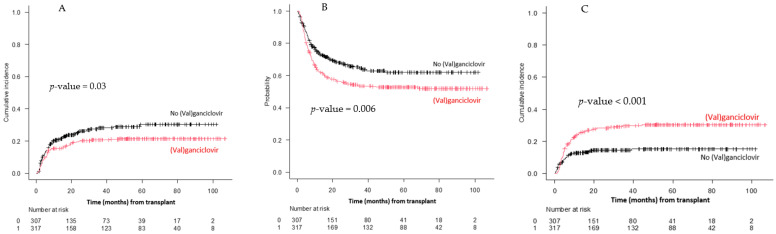
(**A**): Cumulative incidence of relapse (CIR), (**B**): Overall survival (OS), (**C**): Non-relapse mortality (NRM).

**Figure 1:**
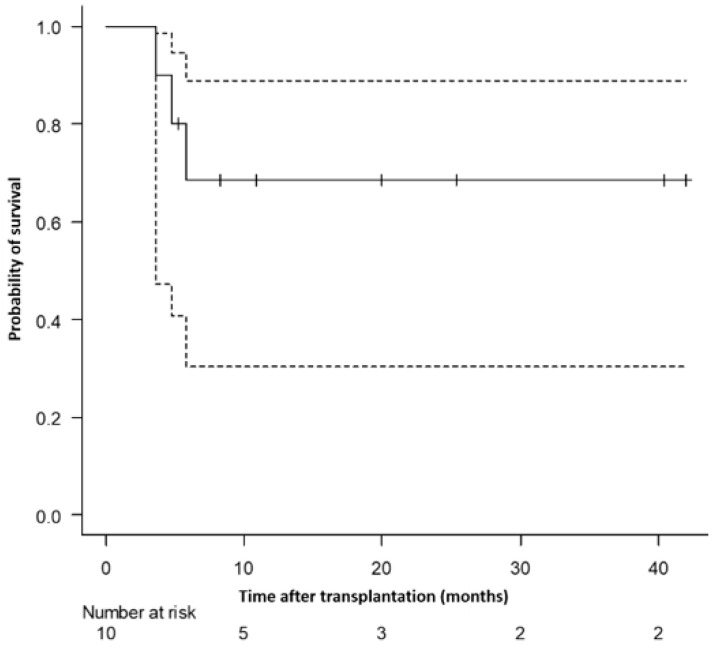
Overall survival. Dotted lines indicate upper and lower limits of probability.

**Figure 1:**
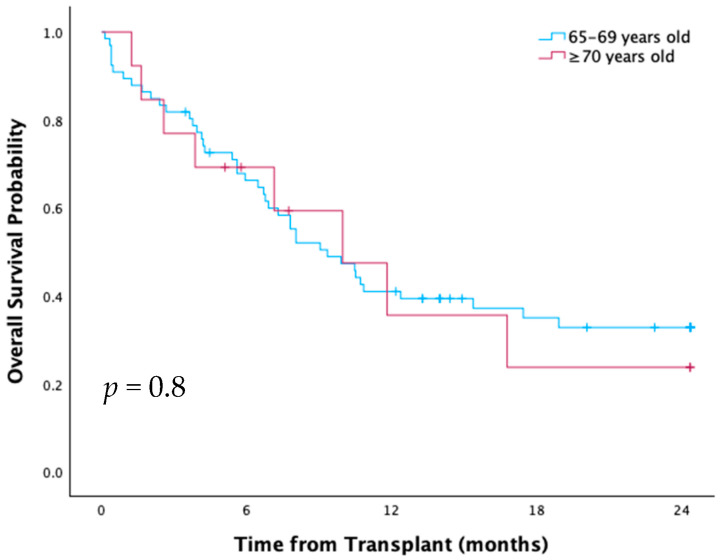
Overall Survival from Transplant Date Compared by Age.

**Figure 2:**
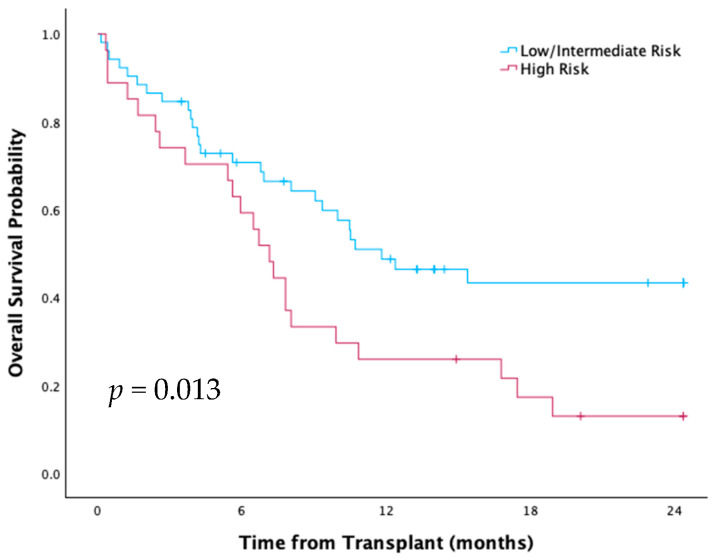
Overall Survival from Transplant Date Compared by HCT-CI Risk.

**Figure 1:**
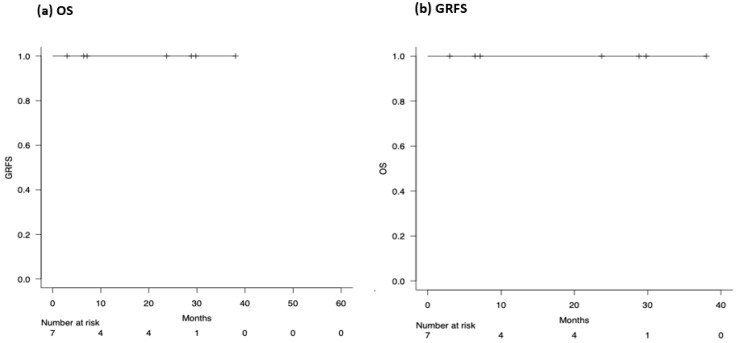
(**a**) Overall Survival (OS), (**b**) Graft-versus-host disease free, relapse-free survival (GRFS).

**Figure 1:**
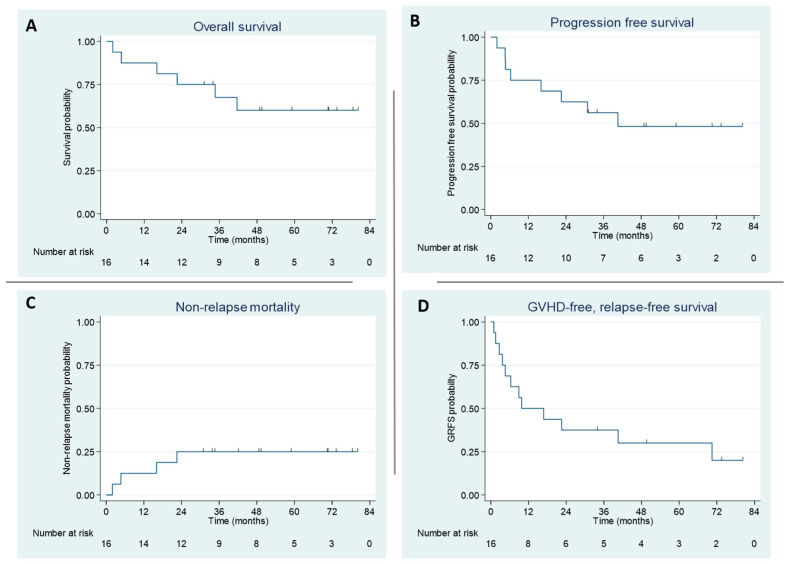
Outcomes of alloSCT in HL patients. (**A**). Overall survival (**B**). Progression-free survival. (**C**). Non-relapse mortality (**D**). GVHD-free, relapse-free survival.

**Figure 1:**
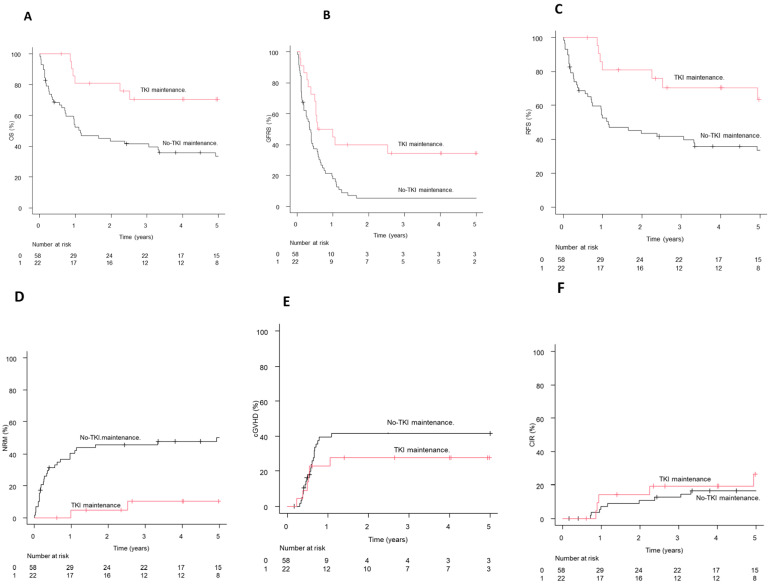
(**A**): overall survival (OS), (**B**): graft-versus-host disease and relapse-free survival (GFRS), (**C**): relapse-free survival (RFS), (**D**): non-relapse mortality (NRM), (**E**): chronic graft-versus-host disease (cGVHD), (**F**): cumulative incidence of relapse (CIR).

**Figure 1:**
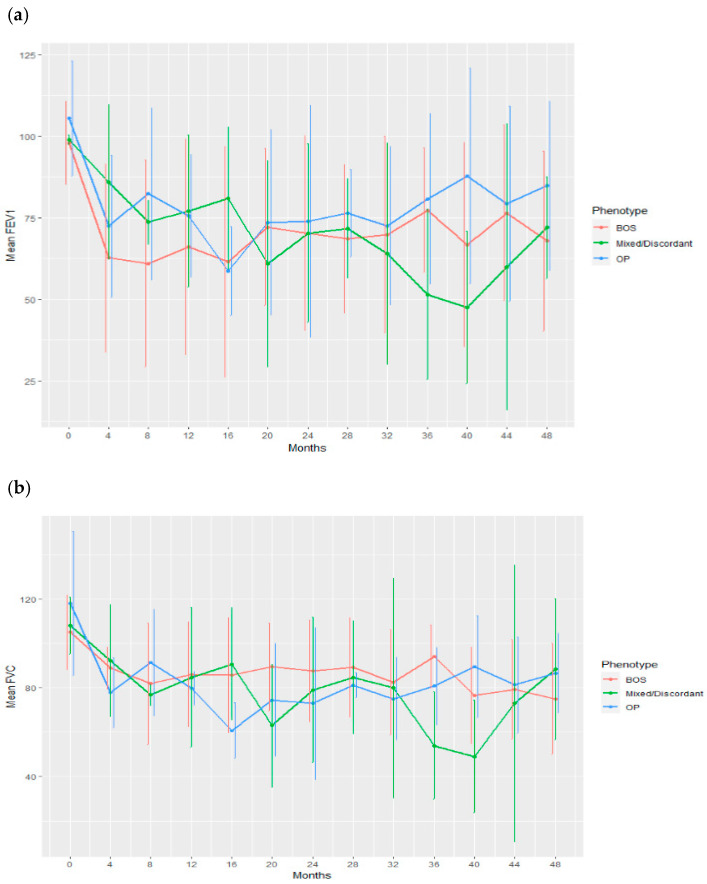
Mean FEV1 (**a**) and mean FVC (**b**) over time for patients with pulmonary GVHD with various phenotypes, namely bronchiolitis obliterans (BOS; red), mixed/discordant radiological picture (green) and with organizing pneumonia (OP, blue).

**Figure 2:**
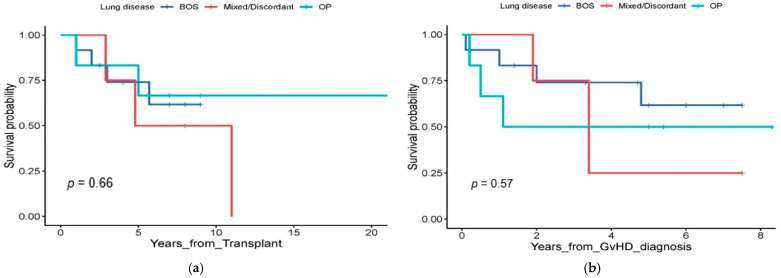
Survival Probability of time from allogeneic transplant (**a**), and time from pulmonary GVHD diagnosis (**b**), over time for patients with pulmonary GVHD with various phenotypes, namely bronchiolitis obliterans (BOS), mixed/discordant radiological picture and with organizing pneumonia (OP).

**Figure 1:**
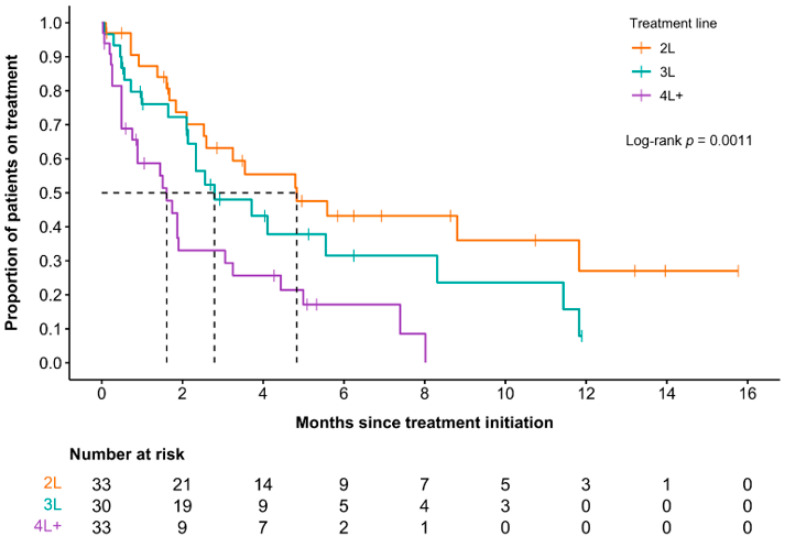
Treatment duration by line of tafasitamab use. The dashed lines indicate the timepoint at which 50% of patients were still on treatment.

**Figure 1:**
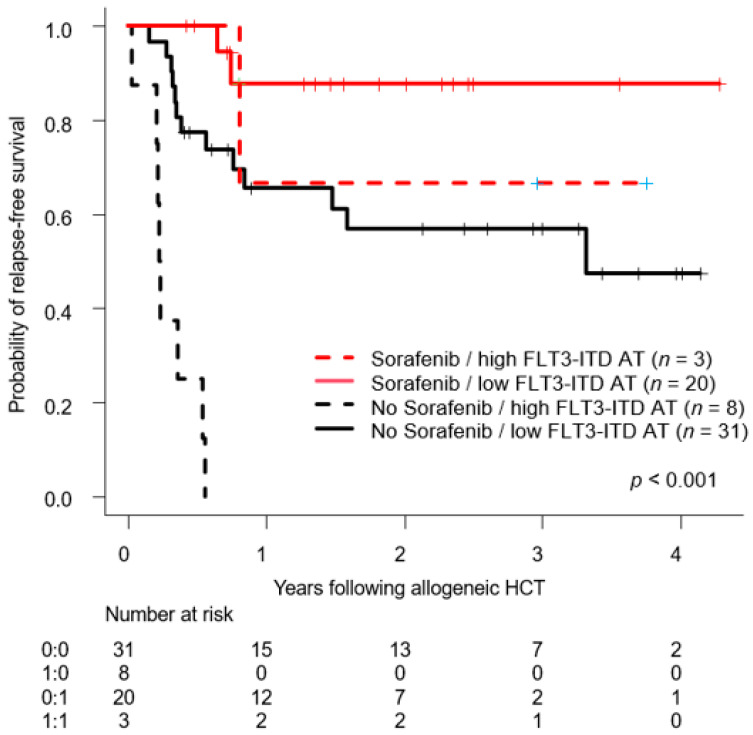
RFS for combined risk using sorafenib maintenance and FLT-ITD AF.

**Figure 1:**
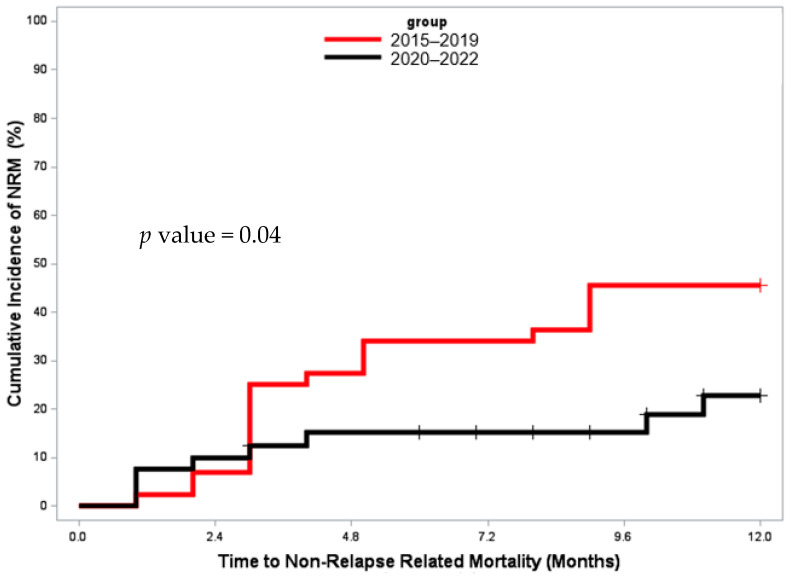
Non-relapse mortality (NRM) in patients ≥70, who received an allogeneic HCT in 2015–2019 vs. 2020–2022.

**Figure 1:**
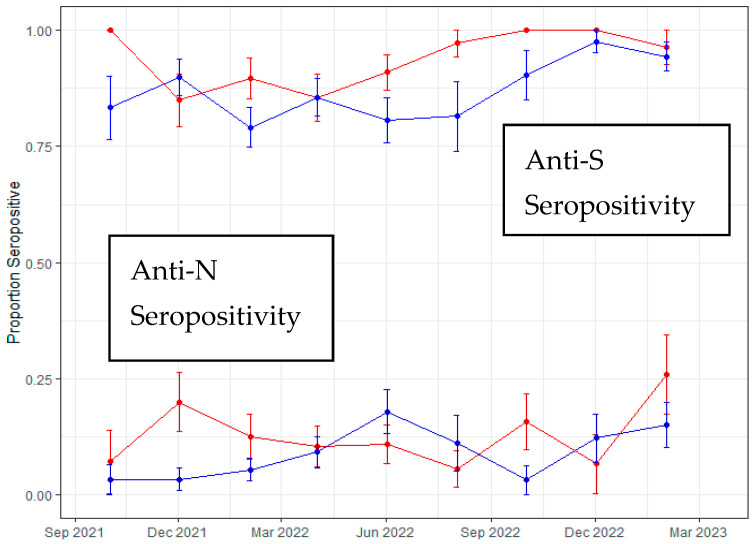
Proportion of cohort with anti-N and anti-S seropositivity over time, by subgroup. Red = AlloSCT, Blue = ASCT.

**Figure 1:**
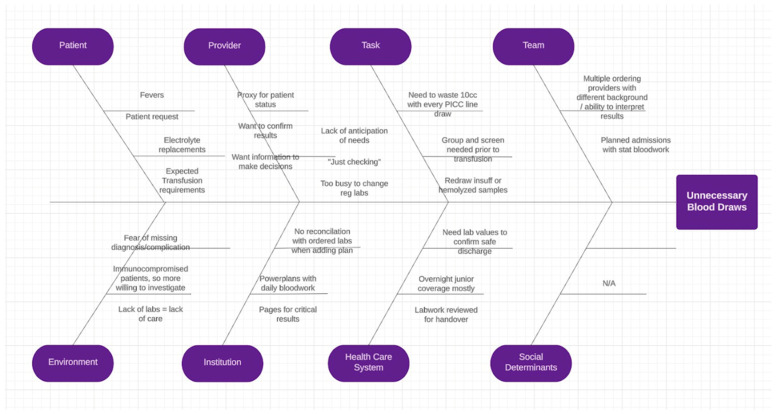
Ishikawa Fishbone Diagram showing the root cause analysis completed.

**Figure 2:**
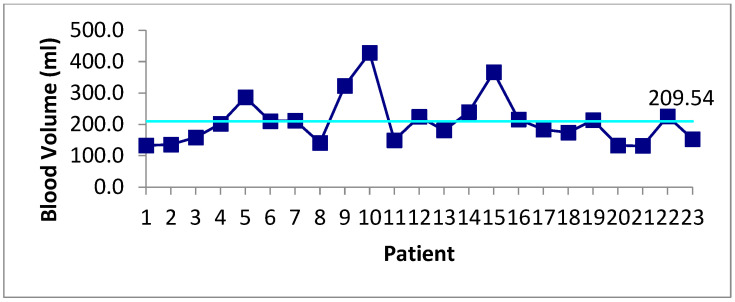
Average daily blood volume drawn for each of 23 ASCT patients. The light blue line indicates the overall average of 209.54 mL.

**Figure 1:**
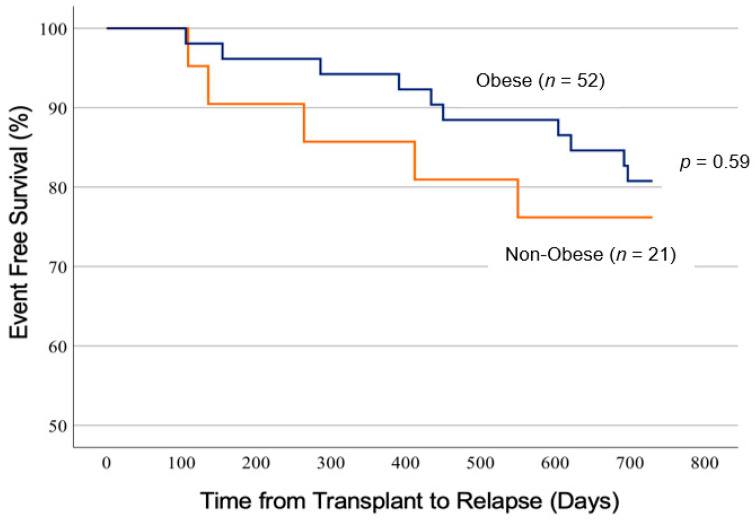
Progression-free survival (PFS) at 2 years in obese (blue) and non-obese (orange) groups.

**Figure 2:**
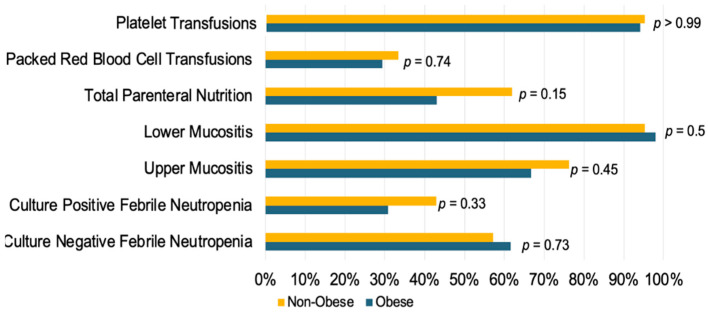
Interventions and side effects in obese and non-obese groups, presented as a percentage of patients who received an intervention or experienced a side effect.

**Table 1:** Secondary malignancies post allogeneic stem cell transplant.

Type of Malignancy	*n*	Months to SM (Median, IQR)	Treatment for SM		
			Surgery	Chemotherapy/ Immunotherapy	Palliation
Hematological malignancies	39	12 (3.1–30.4)			
t-AML	4	15.9 (12.1–27.1)	0	1	3
Multiple Myeloma	2	21.9 (15.7–29.2)	0	2	0
SMM	3	30.5 (17.8–46.6)	0	0	0
T-ALL	1	22.5	0	1	0
PTLD	29	3.2 (2.4–6.4)	0	24	0
Non-hematological malignancies	34	21.9 (11.6–40.7)	28	6	2
Skin Cancers	20	23.2 (12.4–40.5)			
BCC	6	29.2 (16.2–43.8)	6	0	0
SCC	13	22.2 (12.3–36.8)	12	1	0
Melanoma	1	34.3	1	0	0
Renal cell carcinoma	2	5.5 (4.3–7)	1	1	0
Lung cancer	2	42 (34.8–49.8)	1	2	0
Carcinoma endometrium	3	30.9 (22.8–42.5)	2	2	0
CUP	1	27.1	0	0	1
Thymoma	1	21.9	1	0	0
Thyroid cancer	1	47.2	1	0	0
Carcinoma prostate	3	44.5 (34.8–56.8)	3	0	0
Tonsillar carcinoma	1	45.9	0	0	1
Total	73	19.3 (3.1–30.5)	28	34	5

BCC: Basal cell carcinoma; CUP: carcinoma of unknown primary; IQR: interquartile range; PTLD: Post-transplant lymphoproliferative disorder; SCC: squamous cell carcinoma; SMM: Smoldering multiple myeloma; t-AML: therapy related acute myeloid leukemia; T-ALL: T-cell acute lymphoblastic leukemia.

**Table 1:** Patient characteristics and pre- and post-EPAG cost.

	Total (*n* = 39)
Gender, *n* (%)	
Male	27 (69.23%)
Female	12 (30.77%)
Primary disease, *n* (%)	
Myeloid	29 (74.36%)
Non-Lymphoid	10 (25.64%)
Donor, *n* (%)	
MUD	26 (66.67%)
MRD	5 (12.82%)
Haplo	8 (20.51%)
Conditioning, *n* (%)	
MAC	15 (38.46%)
RIC	24 (61.54%)
ABO mismatch, *n* (%)	
Match	18 (46.15%)
Major	9 (23.08%)
Minor	10 (25.64%)
Bi-directional	2 (5.13%)
EPAG indication, *n* (%)	
PGF	35 (89.74%)
Isolated Thrombocytopenia	4 (10.26%)
Concomitant CMV, *n* (%)	
Yes	13 (33.33%)
No	26 (66.67%)
Concomitant GvHD, *n* (%)	
Yes	17 (43.59%)
No	22 (56.41%)
Platelet count at EPAG start, *n* (%)	
<10	17(43.59%)
>20	22 (56.41%)
Weekly transfusions pre-EPAG	
Median (Range)	1.56 (0.06–3.99)
Weekly transfusion post-EPAG	
Median (Range)	0.24 (0.00–7.07)
Total cost pre-EPAG (CAD)	
Median (Range)	75,412.0 (1424.0–217,078.0)
Total actual cost post-EPAG (CAD)	
Median (Range)	50,344.0 (4544.031–7258.0)
Total cost post-EPAG (if early tapered)	
Median (Range)	49,657.0 (4544.0–317,258.0)

**Table 1:** Patient characteristics and multivariable analysis.

	All Patients (624)	(Val)Ganciclovir Exposed (317)	Control (307)	*p*-Value
Age at allo-HCT (median, range)				
	58 (46–65)	58 (46–65)	58 (46–65)	
Age > 60 years (n, %)	320	174 (54.8)	146 (47.6)	0.30
Male (*n*, %)	321 (51.4)	159 (50.2)	162 (52.8)	0.73
Diagnosis (*n*, %)				
AML	491 (78.7)	252 (79.5)	239 (77.9)	0.90
MDS	133 (21.3)	65 (20.5)	68 (22.1)	0.70
KPS < 90 (*n*, %)	117 (18.8)	62 (19.6)	55 (17.9)	0.68
Disease Risk Index (*n*, %)				
High–Very high	124 (19.9)	74 (23.3)	50 (16.2)	0.07
HCT CI (median, range)	2 (1–3)	2 (1–3)	2 (1–3)	
Donor: (*n*, %)				
Matched related	152 (24.4)	70 (22.1)	82 (26.7)	0.32
Matched unrelated	240 (38.5)	126 (39.8)	114 (37.1)	0.70
Mismatched unrelated	75 (12.1)	51 (16.1)	24 (7.8)	0.004
Haploidentical	105 (16.8)	70 (22.1)	35 (11.4)	0.002
Conditioning intensity: (*n*, %)				
Myeloablative	166 (26.6)	63 (19.9)	103 (33.6)	0.003
Reduced intensity	458 (73.4)	254 (80.1)	204 (66.4)	0.13
ATG for GVHD prophylaxis (*n*, %)	500 (80.1)	257 (81.1)	243 (79.2)	0.85
GVHD: (*n*, %)				
Acute GVHD	327 (52.4)	169 (53.3)	158 (51.4)	0.83
Grade II–IV aGVHD	52 (8.3)	37 (11.7)	15 (4.9)	0.005
Chronic GVHD	180 (28.9)	91 (28.7)	89 (28.9)	1.0
Moderate–severe cGVHD	92 (14.7)	56 (17.7)	36 (11.8))	0.07
**Multivariable analysis**				
	**Hazard Ratio**		**95% Confidence Interval**	***p*- ** **value**
Myeloablative conditioning	0.46		0.29–0.71	<0.0001
Mismatched Unrelated Donor	1.71		1.09–2.66	0.01
Exposure to (val)ganciclovir	0.65		0.46–0.90	0.01

Abbreviations: AML: Acute Myeloid Leukemia; ATG: Antithymocyte Globulin; KPS: Kanofsky Performance Status; MDS: Myelodysplastic Syndrome; GVHD: Graft-versus-host disease.

**Table 1:** Baseline characteristics and details of salvage HCT.

Case	Age (Years)	Sex	Disease	Donor 1st HCT	CD34 1st HCT (×10^6^/kg)	Conditioning Regimen 1st HCT	Conditioning Regimen Intensity	GvHD Prophylaxis 1st HCT	Dose of ATG (mg/kg)	Graft Failure	Donor 2nd HCT	CD34 2nd HCT (×10^6^/kg)	GvHD Prophylaxis 2nd HCT	Time Between 1st and 2nd HCT (Days)	ANC Engraftment 2nd HCT (Days)	Pit Engraftment 2nd HCT (Days)	Outcome	Cause of Death
1	75	M	MF	MUD	9.67	Flu Bu2 T200cGy	RIC	ATG-CsA-Mtx	2	Primary	MUD	5.17	CsA-MMF	55	13	18	Dead	Sepsis with multi-organ failure
2	66	F	T-AML	MUD	5.21	Flu Bu2 T200cGy	RIC	ATG-CsA-Mtx	2	Primary	MUD	10.10	CsA-Mtx	69	18	21	Alive	
3	57	M	MF	MUD	4.01	Flu Bu3	MAC	ATG-PTCy-CsA	2	Primary	MSD	10.30	CsA-MMF	43	21	15	Alive	
4	39	F	ATLL	MMUD (DQB1)	5.92	Etoposide-TBI1200cGy	MAC	ATG-PTCy-CsA	4.5	Primary	Haplo	8.36	CsA-Mtx	112	45	Not engrafted	Dead	Sepsis with multi-organ failure
5	34	M	AML	Haplo	5.59	Flu Bu4	MAC	ATG-PTCy-CsA	4.5	Secondary	Haplo *	5.18	CsA-Mtx	82	21	21	Alive	
6	59	M	MF	Haplo	4.77	Flu Bu3	MAC	ATG-PTCy-CsA	4.5	Primary	Haplo	6.06	PTCy-CsA	50	23	30	Alive	
7	56	M	MF	Haplo	5.14	Flu Bu3	MAC	ATG-PTCy-CsA	4.5	Primary	Haplo *	8.13	CsA-MMF	61	14	19	Alive	
8	63	F	MF	MMUD (DQB1)	8.30	Flu Bu2 T200cGy	RIC	ATG-PTCy-CsA	4.5	Secondary	MMUD *	12.13	CsA	107	16	10	Alive	
9	62	M	MF	MUD	4.14	Flu Bu2 T200cGy	RIC	ATG-PTCy-CsA	2	Primary	MUD	5.63	CsA-Mtx	84	48	Not engrafted	Dead	Sepsis with multi-organ failure
10	71	M	T-AML	MUD	5.66	Flu Bu2 T200cGy	RIC	ATG-PTCy-CsA	2	Primary	MUD	8.13	CsA-MMF	65	21	21	Alive	

Abbreviations: HCT: Hematopoietic cell transplant; MF: Myelofibrosis; AML: Acute myeloid leukemia; T-AML: therapy-related acute myeloid leukemia; ATLL: Adult T-cell lymphoma/leukemia; MUD: Matched unrelated donor; MRD: Matched related donor; MMUD: Mismatched unrelated donor; Haplo: Haploidentical; Flu: Fludarabine; Bu: Busulfan; ATG: Anti-thymocyte globulin; CsA: Cyclosponrine A; Mtx: Methotrexate; MMF: Mycophenolate mofetil; *: Same as 1st HCT donor; RIC: Reduced intensity conditioning; MAC: Myeloablative conditioning.

**Table 1:** Baseline clinical and disease characteristics in patients over 65 years old undergoing HCT.

Baseline Characteristics	*N* (%)
Age	
65–69	66 (84%)
≥70	13 (16%)
Karnofsky Performance Status	
90–100%	69 (87%)
60–80%	10 (13%)
HCT-CI	
Low Risk (0)	23 (29%)
Intermediate Risk (1–2)	29 (37%)
High Risk (3–6)	27 (34%)
Diagnosis	
Acute Myeloid Leukemia	40 (51%)
Myelodysplastic Syndrome	18 (23%)
Myeloproliferative Neoplasm	11 (14%)
Non-Hodgkin Lymphoma	5 (6%)
Acute Lymphoblastic Leukemia	3 (4%)
Chronic Lymphocytic Leukemia	2 (2%)
Disease Risk Index	
Intermediate	24 (30%)
High	50 (63%)
Not Available	5 (6%)
Donor Type	
Matched Unrelated Donor	32 (41%)
Matched Related Donor	30 (38%)
Mismatched Unrelated Donor	12 (15%)
Mismatched Related Donor	3 (4%)
Haploidentical Donor	2 (2%)
Year of Transplant	
2012–2017	29 (37%)
2018–2023	50 (63%)

**Table 1:** Baseline characteristics (*n* = 39).

Characteristics	Value
Age (median, range), years	58 (42–63.5)
Male	19 (48.7%)
Indication for alloHCT	
Acute Myeloid Leukemia	23 (58.9%)
Acute Lymphoblastic Leukemia	6 (15.4%)
Myelodysplastic Syndrome	2 (5.1%)
Lymphoma	4 (10.3%)
Others	4 (10.3%)
CMV serology	
Recipient CMV IgG positive	37 (94.9%)
Donor CMV IgG negative	21 (53.8%)
Conditioning Regimen	
Myeloablative	19 (48.7%)
Reduced Intensity	20 (51.3%)
Graft-versus-host disease prophylaxis	
ATG based	32 (82%)
Dual T cell depletion (ATG + PTCy)	27 (69.2%)
Donor	
Matched Related	8 (20.5%)
Matched Unrelated	19 (48.7%)
Mismatched Unrelated	6 (15.4%)
Haploidentical	12 (30.8%)
Stem cell source	
Peripheral blood	37 (94.9%)
Bone marrow	2 (5.1%)
Acute GVHD at the time of Letermovir initiation	
All grades	18 (46.1%)
Grade III/IV	0
CMV viral load (IU/mL) triggering pre-emptive treatment (median, range)	707 (316–1400)
Pre-emptive treatment for CMV before Letermovir initiation	
Valganciclovir	36 (92.3%)
Ganciclovir	6 (15.4%)
Foscarnet	2 (5.1%)
Duration of Letermovir as secondary prophylaxis (median, range), days	77 (46–90)
Reason for Letermovir discontinuation	
Planned completion of secondary prophylaxis	30 (76.9%)
CMV infection requiring treatment	1 (2.6%)
Death	7 (17.9%)
Missing data	1 (2.6%)
Time from HSCT to starting Letermovir as secondary prophylaxis (median, range), days	47 (41–56)
Breakthrough CMV infections during Letermovir use as secondary prophylaxis	1 (2.6%)
CMV infections after discontinuation of Letermovir as secondary prophylaxis	4 (10.3%)
Follow up after stopping Letermovir secondary prophylaxis (median, range), days	287 (29–757)
Relapse	8 (20.5%)
Death	15 (38.5%)

**Table 1:** Patient demographic and clinical characteristics.

Variable	*N* (%) or Median (Range)
Age	30 (20–48)
Donor age (median)	28 (17–46)
Gender:	
Male	6 (85.7%)
Female	1 (14.3%)
ABO blood type compatibility:	
Matched	5 (71.4%)
Minor mismatch	1 (14.3%)
Major mismatch	1 (14.3%)
Sickle cell subtype:	
HbSS	6 (85.7%)
HbSC	1 (14.3%)
Transplant indications:	
Recurrent VOC	6 (85.7%)
ACS	5 (71.4%)
Stroke	1 (14.3%)
Priapism	2 (21.6%)
Patient ethnicity:	
Black–Africa	3 (42.8%)
Black–Caribbean	3 (42.8%)
Middle East–Arab	1 (14.3%)
KPS:	
>90%	7 (100%)
Stem cell collection days:	
1 day	5 (71.4%)
2 days	2 (21.6%)
CD34 dose	8.9 (7.81–11.27)
Female donor to male recipient	6 (85.7%)
CMV mismatch	3 (42.8%)
Positive EBV IgG	7 (100%)
Median HCT-Cl	3 (0–5)
Median hospitalization days	33 (27–41)
Follow up (months)	23.7 (3–38.03)

**Table 2:** Chimerism follow up.

	T-Cell Lineage Day 30	Myeloid Lineage Day 30	T-Cell Lineage Day 60	Myeloid Lineage Day 60
1	7	95.6	6.5	97.7
2	66.9	97.7	32.9	96.2
3	40.4	99.9	5.1	98.5
4	19.2	99.1	4.2	99
5	inconclusive	96.8	33.2	86
6	84.2	97.9	55.9	96.9
7	11.5	98.6	-	-

**Table 1:** Patients’ characteristics.

Variables	TKI Maintenance *N* = 22	No TKI-Maintenance *N* = 58	*p*
Median age (range)	54 (19–63)	44 (19–65)	0.2
Gender, *n* (%)			0.09
Female	14 (63.6%)	24 (41.4%)	
Male	8 (36.4%)	34 (58.6%)	
Disease status, *n* (%)			0.04
CR1	15 (68.2%)	52 (89.7%)	
CR2	7 (31.8%)	6 (10.3%)	
BCR/ABL pre-HCT, *n* (%)			0.57
MMR	18 (81.8)	43 (74.1)	
No MMR	4 (18.2)	15 (25.9)	
Conditioning regimen, *n* (%)			0.09
MAC	13 (59.1%)	46 (79.3%)	
RIC	9 (40.9%)	12 (20.7%)	
GVHD prophylaxis, *n* (%)			0.005
Dual T-cell depletion	8 (36.4)	5 (8.6)	
Others	14 (63.6)	53 (91.4)	
PTCy based GVHD, *n* (%)			<0.001
Yes	10 (45.5)	5 (8.6)	
No	12 (54.5)	53 (91.4)	
Donor type, *n* (%)			0.17
MRD	9 (40.9)	27 (46.6)	
MUD	10 (45.5)	23 (39.7)	
MMUD	0	6 (10.3)	
Stem sources			0.49
PBSC	20 (90.9)	48 (82.8)	
BMSC	2 (9.1)	10 (17.2)	
ABL Kinase mutation			0.03
Positive	5 (22.7)	3 (5.2)	
Negative	17 (77.3)	55 (94.8)	
OS			0.02
Alive	14 (63.6%)	19 (32.8%)	
Death	8 (36.4%)	39 (67.2%)	
NRM			0.005
Alive	19 (86.4%)	28 (48.3)	
Death	3 (13.6%)	30 (51.7%)	
RFS			0.04
No	13 (59.1%)	18 (31%)	
Yes	9 (40.9%)	40 (69%)	
Relapse			0.49
No	16 (72.7)	48 (82.8)	
Yes	6 (27.3)	10 (17.2)	
Chronic GVHD			0.79
Yes	7 (31.8)	22 (37.9)	
No	15 (68.2)	36 (62.1)	
Acute GVHD			0.79
Yes	14 (63.6)	39 (67.2)	
No	8 (36.4)	19 (32.8)	

Abbreviation: BM: bone marrow, CR: complete remission, HCT: allogenic hematopoietic cell transplantation, MAC: myeloablative conditioning, MMR: Major molecular response, MRD: matched related donor, MUD: matched unrelated donor, MMUD: mismatched unrelated donor, NRM: non-relapse mortality, OS: overall survival, PBSC: peripheral blood stem cell transplant, PTCy: post-transplant cyclophosphamide, RFS: relapse-free survival, RIC: reduced-intensity conditioning.

**Table 1:** Cox regression analysis of overall and progression-free survival with repeated measures of patients undergoing CAR-T therapy at Princess Margaret Cancer Centre.

	Univariable Cox Regression				Multivariable Cox Regression			
	Overall Survival		Progression Free Survival		Overall Survival		Progression Free Survival	
	HR (95% CI)	*p*	HR (95% CI)	*p*	HR (95% CI)	*p*	HR (95% CI)	*p*
Clinical Frailty Scale	1.61 (1.28, 2.02)	<0.001	1.38 (1.14, 1.67)	0.001				
Clinical Frailty Scale (Categorical)		0.002		0.008		0.021		0.028
1–3	Reference		Reference		Reference		Reference	
>3	3.32 (1.54, 7.16)		2.34 (1.25, 4.40)		2.41 (1.14, 5.07)		2.39 (1.10, 5.18)	
Grip strength (kg)	0.99 (0.94, 1.03)	0.516	0.99 (0.96, 1.04)	0.862				
Gait Speed (m/s)	0.18 (0.03, 1.08)	0.060	0.26 (0.05, 1.24)	0.092				
Gait Speed (m/s) (Categorical)		0.002		0.021		0.093		0.7333
≤0.80	Reference		Reference		Reference		Reference	
>0.80	0.30 (0.14, 0.63)		0.40 (0.18, 0.87)		0.47		0.85 (0.34, 2.16)	
LDH	1.004 (1.002, 1.006)	<0.001	1.003 (1.001, 1.004)	0.003				
CRP	1.004 (1.002, 1.006)	<0.001						
VES-13	1.31 (1.08, 1.58)	0.005	1.22 (1.03, 1.45)	0.018				
VES-13 (Categorical)		0.006		0.011		0.020		0.052
<3	Reference		Reference		Reference		Reference	
≥3	4.62 (1.56, 13.6)		3.46 (1.34, 8.95)		3.57 (1.22, 10.5)		2.63 (0.99, 6.96)	
ECOG		0.056		0.075				
0/1	Reference		Reference					
2/3	3.06 (0.97, 9.66)		2.45 (0.91, 6.57)					

**Table 1:** Patient treatment summary.

Patient	Auto-SCT	Allo-SCT	Acute GVHD	Chronic GVHD	Treatment	Timing of Brentuximab and PD1 Inhibitors Prior to Allo-SCT
1	April 2016 DOR: 2 months	May 2021 DOR: 12 months OS: 12 months	skin		Brentuximab and Cyclophosphamide	Brentuximab April 2017 (48 months) Pembrolizumab August 2019 (21 months) Nivolumab December 2020 (6 months)
2	March 2015 DOR: 21 months	May 2021 DOR: 11 months OS: ongoing		Liver grade 1	Everolimus	Pembrolizumab November 2017 (43 months) Nivolumab March 2018 (38 months)
3	July 2018 DOR: 17 months	September 2021 DOR: 17 months OS: ongoing	Skin and GI		Radiation and Everolimus	Brentuximab February 2020 (14 months) Pembrolizumab October 2020 (11 months)
4	July 2019 DOR: 2 months	September 2021 DOR: 16 months OS: 24 months				Nivolumab September 2020 (12 months)
5	Not done	November 2021 DOR: 8 months OS: 8 months				Brentuximab June 2020 (17 months) Pembrolizumab September 2020 (14 months)

**Table 1:** Characteristics of GO and control patients.

	GO	Control
Mean age, years (range)	51 (19–71)	52 (23–71)
Male, number (percent)	10 (63)	10 (63)
Duration of follow-up, months (range)	12.8 (2–44)	39.8 (9–95)
AML risk category		
Favorable	8	8
Mutated NPM1	5	3
inv (16)	0	2
t (8; 21)	3	3
Intermediate	8	8
Unfavorable	0	0

**Table 2:** Outcomes after induction of GO versus control patients.

Parameter	GO	Control	Statistics
LOS after induction, days	37.9	37.8	HR 0.879 (95% CI 0.4343 to 1.777)
Induction to ANC > 1.0, days	32.4	30.5	HR 0.789 (95% CI 0.39 to 1.59)
Induction to Plt recovery > 50, days	26.8	27	HR 0.575 (95% CI 0.277 to 1.195)
Induction to Plt recovery > 100, days	28.5	30.8	HR 0.692 (95% CI 0.330 to 1.453)
CR, percent	88	94	OR 0.47 (95% CI 0.04 to 5.74)
Relapse, percent	19	19	OR 1.00 (95% CI 0.17 to 5.90)
Time of CR to relapse, days	312.7	356.0	HR 1.647 (95% CI 0.316 to 8.579)
Liver toxicity, percent	19	25	OR 0.69 (95% CI 0.13 to 3.75)
SCT, percent	25.0	37.5	OR 0.67 (95% CI 0.16 to 2.82)
Time of CR to SCT, days	329.8	200.0	HR 0.524 (95% CI 0.150 to 1.825)
Survival, percent	87.5	75.0	HR 0.50 (95% CI 0.102 to 2.502)

**Table 1:** Responses to axatilimab in fibrosis-dominant organs.

% (*n*/*N*)	0.3 mg/kg Q2W (*n* = 80)	1 mg/kg Q2W (*n* = 81)	3 mg/kg Q4W (*n* = 80)	Overall (*N* = 241)
Esophagus response *	78 (18/23)	61 (11/18)	60 (12/20)	67 (41/61)
Joints/fascia response *	76 (42/55)	63 (35/56)	57 (29/51)	65 (106/162)
P-ROM ^†^	55 (27/49)	36 (18/50)	42 (18/43)	44 (63/142)
Lung response *	47 (15/32)	34 (14/41)	37 (13/35)	39 (42/108)
Skin response *	27 (17/64)	11 (7/63)	23 (15/66)	20 (39/193)
Skin/joint tightening 1-point improvement ^‡^	66 (35/53)	56 (31/55)	60 (34/57)	61 (100/165)
Skin/joint tightening 2-point improvement ^‡^	47 (25/53)	35 (19/55)	37 (21/57)	39 (65/165)
Sclerotic skin BSA reduction ^§^	44 (19/43)	34 (15/44)	60 (28/47)	46 (62/134)
Thickened skin (mLSS) reported improvement ^¶^	73 (33/45)	77 (33/43)	68 (36/53)	72 (102/141)

BSA, body surface area; mLSS, modified Lee Symptom Score; P-ROM, photographic range of motion; Q2W, every 2 weeks; Q4W, every 4 weeks. Denominator is the number of patients with an assessment at baseline. * Per 2014 National Institutes of Health chronic graft-versus-host disease consensus guidelines. ^†^ Defined as ≥2-point improvement in P-ROM score (range, 0–25). ^‡^ Improvement in severity of skin and/or joint tightening (clinician assessment; range, 0–10). ^§^ Reduction in BSA involvement by sclerotic skin (any amount, regardless of skin response). ^¶^ Defined as ≥1-point improvement in mLSS thickened skin subdomain (range, 0–4).

**Table 1:** Patient and transplant variables, and outcomes at one year.

	Total (*N* = 84)	Cohort A (2015–2019) (*N* = 44)	Cohort B (2020–2022) (*N* = 40)	*p*-Value
Age at BMT				
Median (range)	71 (70–76)	71 (70–76)	71 (70–75)	0.47
Gender, *n* (%)				
Female	30 (35.71%)	14 (31.82%)	16 (40%)	
Male	54 (64.29%)	30 (68.18%)	24 (60%)	
Diagnosis, *n* (%)				
AML	46 (54.76%)	28 (63.64%)	18 (45.00%)	
MDS/MDS-MPN/CMML	27 (32.14%)	12 (27.27%)	15 (37.50%)	
MF/ET/MPN-unclassifiable	7 (8.33%)	3 (6.82%)	4 (10.00%)	
t-ALL/TPALL	2 (2.38%)	1 (2.27%)	1 (2.50%)	
Others	2 (2.38%)	0 (0.00%)	2 (5.00%)	
CD34 cell count/kg, *n* (%)				
Median (range)	6.9 (2.2–20.1)	7.4 (2.2–20.1)	6.6 (3.6–10.2)	0.026
Conditioning regimen, *n* (%)				
Flu(4)-Bu(2)-TBI(200)	73 (86.90%)	44 (100%)	29(72.5%)	<0.001
Flu-Treo	10	0	10 (25%)	
Melphalan 140 mg/m^2^	1	0	1 (2.5%)	
GVHD prophylaxis, *n* (%)				
ATG(2)-PTCy-CSA	32 (38.55%)	12 (27.27%)	20 (51.28%)	0.002
ATG(4.5)-PTCy-CSA	35 (42.17%)	24 (54.55%)	11 (28.21%)	
ATG(2)-CSA-MTX	3 (3.61%)	0 (0%)	3 (7.69%)	
ATG(4.5)-CSA-MTX	7 (8.43%)	6 (13.64%)	1 (2.56%)	
Other	6 (7.22%)	2 (4.54%)	4 (10.25)	
KPS, *n* (%)				
70–80	17 (20.24%)	10 (22.73%)	7 (17.50%)	0.55
90–100	67 (79.76%)	34 (77.2%)	33 (82.50%)	
Cause of death, *n* (%)				
Relapse	16 (34.04%)	9 (28.13%)	7 (46.67%)	
Infection	18 (38.30%)	15 (46.88%)	3 (20%)	
GVHD	6 (12.77%)	6 (18.75%)	0 (0.00%)	
Other	7 (14.89%)	2 (6.25%)	5 (33.33%)	
OS, (%)				
1-year	53%	45%	62%	0.10
EFS, (%)				
1-year	45%	37%	58%	0.07
NRM, (%)				
1-year	35%	45%	23%	0.04

Flu = fludarabine, Bu = Busulfan, Treo = treosulfan, PTCy = post-transplant cyclophosphamide, CSA = cyclosporine, MTX = methotrexate.

**Table 1:** Incidence of positive culture infections in HCT patients three months post-transplant.

Infection Type	Episodes, *n* (% of 52)	Individuals, *n* (% of 60)
Viral Infections	23 (44)	19 (32)
Cytomegalovirus (CMV)	10	10
Rhinovirus	5	5
BK Virus	4	3
Coronavirus (various strains)	4	3
Bacterial Bloodstream Infections	12 (23)	9 (15)
Staphylococcus epidermidis (non-S. aureus)	5	5
ESBL Klebsiella pneumoniae	3	3
Enterococcus faecium	1	1
Enterococcus faecalis	1	1
Vancomycin-resistant Enterococcus (VRE)	1	1
Stenotrophomonas maltophilia	1	1
Fungal Bloodstream Infections	3 (6)	2 (3)
Rhodotorula mucilaginosa	3	2
Other Infections	2 (4)	2 (3)
Escherichia coli (urine)	1	1
Rhizomucor (respiratory)	1	1
*Clostridioides difficile* (*C.difficile*)	12 (23)	10 (10)

**Table 1:** Summary of key metabolites elevated or depressed at cGvHD onset and critical statistical values showing significant differences between control and pathological groups.

Metabolite	Type of Metabolite	Effect Ratio	*p*-Value	AUC
Mixed analysis—onset versus no cGvHD only				
Trimethylamine N-oxide	Amine oxide	0.58	0.002	0.74
Hippuric Acid	Carboxylic acid	0.62	0.02	0.71
Fixed analysis—3 months (onset versus no cGvHD only)				
Trimethylamine N-oxide	Amine oxide	0.35	0.02	0.74
Hippuric Acid	Carboxylic acid	0.52	0.05	0.74
Fixed analysis—6 month (onset versus no cGvHD only)				
Trimethylamine N-oxide	Amine oxide	0.61	0.02	0.73
Fixed analysis—12 months (onset versus no cGvHD only)				
Indole acetic acid	Monocarboxylic acid	1.4	0.001	0.77

**Table 1:** Viability.

Cell Type	Haploidentical Fresh (*N* = 87)	Matched Related Donor Fresh (*N* = 94)	Unrelated Donor Fresh (*N* = 244)	Haploidentical Thawed (*N* = 18)	Matched Related Donor Thawed (*N* = 9)	Unrelated Donor Thawed (*N* = 93)	Overall Fresh Graft (*N* = 425)	Overall Thawed Graft (*N* = 120)
CD34	100% (93–100)	100% (95–100)	99% (80–100)	86% (55–92)	87% (76–93)	88% (20–97)	99% (80–100)	87% (20–97)
CD3	99.9% (99–100)	100% (99.7–100)	98.3% (86–100)	74.4% (53.3–92.2)	73.9% (59–91)	66.4% (27.7–92.2)	99.7% (86–100)	68.2% (27.7–92.2)
CD4	100% (99.6–100)	100% (99.8–100)	98% (84–100)	70.4% (39.8–91.1)	72.9% (51.3–99.9)	59.6% (21.9–99.9)	99.6% (84–100)	62% (21.9–99.9)
CD8	99.9% (99–100)	99.9% (99–100)	99% (85.7–100)	82.9% (66.2–96.6)	82.9% (77.1–99.9)	74.4% (34.7–99.9)	99.7% (85.7–100)	78% (34.7–99.9)
CD56	100% (99.2–100)	100% (99.5–100)	99.7% (86–100)	95.2% (83.9–97.8)	94.8% (82–96.8)	94.4% (43.7–98.3)	99.9% (86–100)	94.6% (43.7–98.3)
CD19	99.9% (97.6–100)	99.9% (98.4–100)	99.8% (94–100)	96.4% (89.2–97.8)	96% (93.4–98.2)	94.8% (52–99.1)	99.9% (94–100)	95.8% (52–99.1)
CD14	99.9% (93.7–100)	99.9% (98.8–100)	99.5% (93–100)	94.1% (83.2–98.4)	93.5% (87.3–98.4)	95% (61.4–99.1)	99.7% (93–100)	95% (61.4–99.1)

**Table 2:** *Ex vivo* time, donor age and numbers of donor types that were fresh or cryopreserved.

Characteristic	Fresh	Thawed	*p*-Value
*Ex vivo* Time in hours	26.7 (2.2–64.5)	33 (1.6–87.6)	0.43
Donor Age in years	34 (13–72)	28 (18–65)	<0.001
Donor type			
Haploidentical	69 (22.6%)	18 (15%)	
Matched Related	85 (27.8%)	9 (7.5%)	
Match Unrelated	151 (49.5%)	93 (77.5%)	

## Data Availability

The raw data supporting the conclusions of each abstract will be made available by the authors on request.

